# Comparative Evaluation of ChatGPT, DeepSeek, and Physician-Generated Acupuncture Treatment Protocols Across Chinese and English Language Settings: Cross-Sectional Evaluation Study

**DOI:** 10.2196/95851

**Published:** 2026-10-08

**Authors:** Xin Liu, Zining Guo

**Affiliations:** 1 Thomas College of Business & Economics University of North Carolina at Pembroke Pembroke, NC United States; 2 Northeast Agricultural University Harbin, Heilongjiang China

**Keywords:** integrative medicine, traditional Chinese medicine, TCM, acupuncture, ChatGPT, DeepSeek, AI, acupoint selection, language corpora, large language model

## Abstract

**Background:**

Traditional Chinese medicine (TCM) has received increasing attention in evidence-based medicine. However, its largely experience-based and practice-oriented nature has slowed modernization and standardization. Advances in AI provide new opportunities for integrating AI with TCM.

**Objective:**

This study evaluates the application of large language models (LLMs), including ChatGPT (OpenAI) and DeepSeek, in generating acupuncture treatment protocols. We compare the validity and clinical effectiveness of physician-generated protocols with those produced by LLMs, examine differences in outputs across language corpora (Chinese and English), and establish a standardized assessment framework for systematically assessing the effectiveness of acupuncture point selection. Ultimately, this research aims to support the standardization of acupoint selection and promote greater consistency, reliability, and evidence-based development in acupuncture practice.

**Methods:**

Qualified acupuncture treatment cases published in *Acupuncture in Medicine* were identified and translated from English into Chinese to create a bilingual case dataset. Standardized case prompts were independently input into DeepSeek and ChatGPT to generate acupuncture treatment protocols. Ten senior TCM physicians evaluated the AI-generated and physician-generated protocols using 7 criteria: local point selection, distal point selection, syndrome-based point selection, meridian-tracing point selection, neuroanatomical point selection, core acupoint selection, and synergistic effects. Repeated-measures ANOVA with sphericity assessment was used to examine differences across LLMs, language corpora, and protocol groups.

**Results:**

Blinded expert evaluations revealed significant differences among the 5 acupuncture groups for local point selection (*P*=.006), syndrome-based point selection (*P*=.005), neuroanatomical point selection (*P*<.001), and core acupoints coverage principle (*P*=.03). For local point selection, DeepSeek Chinese output received higher scores than the other evaluated outputs, while ChatGPT English output scored higher than ChatGPT Chinese output. For syndrome-based point selection, the journal physician-generated protocols received lower scores than the DeepSeek Chinese, DeepSeek English, and ChatGPT Chinese outputs. For neuroanatomical point selection, DeepSeek English output achieved the highest scores among the 5 groups. For core acupoints coverage, the journal physician-generated protocols received lower scores than the DeepSeek Chinese, DeepSeek English, and ChatGPT English outputs. No significant differences were observed among the 5 groups for distal point selection, meridian-tracing point selection, or therapeutic synergy. Overall, DeepSeek performed better in the Chinese-language setting, whereas ChatGPT performed better in the English-language setting.

**Conclusions:**

AI has demonstrated substantial progress in encoding explicit acupuncture knowledge and performing systematic acupoint selection. However, challenges remain in individualized treatment and consistency across linguistic contexts. Enhancing bilingual or multilingual training corpora and developing syndrome-specific reasoning modules may improve the clinical applicability of AI-assisted TCM systems.

## Introduction

### Background

Although the modern medical system demonstrates considerable advancement in emergency care and technical interventions, it continues to encounter major challenges in the management of chronic pain, functional disorders, and psychosomatic conditions [1]. This gap in comprehensive care has heightened scholarly and clinical interest in complementary and alternative medicine (CAM) and traditional medicine (TM), with particular attention to traditional Chinese medicine (TCM). Among the diverse modalities within TCM, acupuncture has achieved broad international recognition for its therapeutic efficacy in pain management [2], mental health disorders [3], and other clinical conditions. In the United States, where health care costs remain exceptionally high—per capita medical expenditure constitutes 17.6% of GDP [4]—acupuncture presents notable advantages. Its minimal invasiveness (which limits adverse effects) [5], strong clinical effectiveness, and substantially lower costs compared with pharmacological or surgical treatments [6] underscore its potential as a viable and cost-effective therapeutic option.

However, the development of acupuncture treatment still faces several critical obstacles. The diagnostic and therapeutic processes of acupuncture typically rely on highly experienced practitioners [7]. Acupuncture point selection is also largely experience-driven, and the therapeutic effectiveness of specific point combinations remains difficult to assess using quantitative measures. Recently, generative AI tools such as ChatGPT and DeepSeek have begun to be applied in acupuncture diagnosis and treatment [8]. However, research has shown that AI models trained primarily in English perform effectively on tasks tested in English yet display significant limitations in cross-lingual transfer [9]. This finding underscores the importance of the training language environment, which substantially influences the effectiveness of AI-assisted acupoint selection in medical cases.

### TM and Acupuncture

TM and CAM are increasingly integrated into modern health care. In 2018, the World Health Organization (WHO) announced the inclusion of a “Traditional Medicine Chapter” in *ICD-11* (*International Classification of Diseases, 11th Revision*), formally launched in 2022 [10]. Population-based surveys further demonstrate the growing use of TM, particularly for pain management [11]. Since 2020, the US Centers for Medicare & Medicaid Services has provided coverage for acupuncture treatment for chronic lower back pain [12,13]. Among various forms of traditional Chinese interventions, acupuncture currently possesses the most substantial evidence in mainstream medical journals.

The WHO defines acupuncture as “the insertion of needles into humans or animals for remedial purposes” [14]. Evidence supports its use for chronic diseases, pain, radiotherapy- and chemotherapy-induced nausea and vomiting, as well as menopausal hot flashes [15-18]. A large meta-analysis based on individual patient data characterized its therapeutic effects on various types of chronic pain as “small to moderate” [19]. Similarly, a coherent review concluded that acupuncture could reduce the frequency of headache attacks in patients with episodic migraine [16]. Acupuncture has also been incorporated into military medicine, including battlefield acupuncture (BFA) for pain management [20].

Acupuncture is rooted in TCM theory, which emphasizes the identification of a patient’s Zheng and individualized treatment [14]. Zheng (an abstract pattern of pathological disharmony) represents a comprehensive description of the cause, location, nature, and development of illness at a particular stage [21]. In clinical practice, Zheng can further stratify patients when integrated with biomedical diagnosis, potentially improving treatment efficacy [22].

TCM treatment follows the principle of syndrome differentiation and treatment: clinicians first collect and interpret information through the Four Diagnostic Examinations—inspection, listening and smelling, inquiry, and palpation—to determine the patient’s Zheng (pattern), and then formulate treatment accordingly [14,23]. This differs fundamentally from Western medicine, which primarily classifies diseases using pathophysiology, biomarkers, imaging, microbiology, and standardized diagnostic criteria and selects treatments based on established evidence [24]. TCM’s emphasis on individualized treatment according to syndrome, constitution, age, gender, and lifestyle creates inherent challenges for quantification and standardization [25].

Several efforts have sought to improve the reproducibility and standardization of acupuncture research and practice. The WHO has published standards addressing acupuncture point locations, practice, and training. However, these resources mainly focus on nomenclature and trial reporting rather than establishing a validated quantitative standard for evaluating the appropriateness of point selection. Research has demonstrated substantial heterogeneity in syndrome differentiation and acupoint selection, as well as variation in practitioner agreement and reliability [26,27]. Therefore, acupuncture point selection remains strongly dependent on clinicians’ experience and judgment, highlighting the need for objective and standardized evaluation methods.

### AI Applications in Acupuncture

AI, particularly generative AI and large language models (LLMs), is rapidly transforming health care [28,29]. Applications include automatic prescreening and medical imaging, clinical documentation, disease prevention, and clinical decision support [30-33]. Although AI can improve diagnostic accuracy and optimize resource usage, its effectiveness varies substantially by implementation context [34].

LLMs have demonstrated promising capabilities in medical knowledge and clinical reasoning. ChatGPT has performed near or above passing standards on portions of the USMLE (United States Medical Licensing Examination) and has been evaluated for clinical question answering, document assistance, and patient information provision [35]. It has also shown potential in complex treatment planning and diagnostic tasks [36,37]. Similarly, DeepSeek models have demonstrated promise in clinical diagnosis, decision support, and multidisciplinary medical applications [38-40].

However, AI performance varies across languages and domains. Multilingual LLMs generally perform better in English than in low-resource languages, with reported performance gaps up to 25% [41]. A similar pattern is observed in TCM. Research indicates that domain specialization has a substantial impact on TCM performance: models trained specifically on TCM data significantly outperform general models [42]. Models pretrained on large Chinese corpora or high-quality TCM datasets, such as DeepSeek and PULSE, demonstrate superior capabilities in understanding TCM theories, classical texts, and clinical diagnosis and decision-making compared with international models [42]. Moreover, TCM literature often uses classical Chinese with complex and sometimes obscure logical relationships, making it difficult to interpret without specialized knowledge [43]. English translations of TCM terms have received limited attention in both foreign language and translation studies.

### Research Objectives

Our study aims to establish a standardized assessment framework for acupuncture therapy that enables systematic assessment of the therapeutic effectiveness of acupuncture point selection. Using this framework, we evaluate the performance of ChatGPT and DeepSeek in generating acupuncture treatment protocols based on real clinical cases published in an academic acupuncture journal in both Chinese and English, thereby examining the influence of language on AI-generated recommendations. We also compare AI-generated protocols with physician-developed protocols reported in these clinical cases. Through this comparison, we evaluate the potential of generative AI to support and enhance the effectiveness of acupuncture point selection and treatment planning.

## Methods

### Study Design

Our study used a mixed-methods approach to systematically evaluate the potential application of AI in TCM acupuncture diagnosis and treatment.

### Construct Assessment Criteria

At the outset, an extensive literature review was conducted to identify clinical criteria for acupoint selection. A targeted review of English and Chinese publications (1990-2025) was performed using PubMed, Google Scholar, and Web of Science. Search terms included “TCM,” “acupuncture,” “acupoint selection,” “acupuncture principles,” “syndrome differentiation,” “meridian,” “neuroanatomical acupuncture,” “clinical decision-making,” and “acupuncture education.” Initially, 112 papers were identified. Titles and abstracts were screened for relevance to this study’s scope, resulting in the exclusion of 79 papers because they were outside the scope, provided insufficient information related to this review’s main themes, or were duplicates. The remaining 33 papers were retained for full-text examination. Based on the synthesized literature, 8 preliminary evaluation criteria were established: local point selection, distal point selection, syndrome-based point selection, meridian-tracing point selection, neuroanatomical point selection, core acupoint coverage rate, acupoint synergistic effect, and acupoint antagonistic effect. To evaluate their validity and refine the preliminary criteria, 3 senior TCM physicians with 17, 25, and 32 years of acupuncture experience from a grade A tertiary TCM hospital in east-central China were recruited voluntarily. In June 2025, together with the 3 physicians, we reviewed the literature retrieved from the database search, including classical texts, research papers, medical guidelines, and textbooks. We also incorporated the physicians’ clinical experience to validate the initial set of acupoint selection criteria. After multiple rounds of discussions, we revised the initial criteria and removed the acupoint antagonistic effect from the final evaluation framework due to insufficient evidence (details of the assessment criteria will be discussed later in the Medical Expert Assessment section).

### Identify Qualified Acupuncture Medical Case Reports

Subsequently, a systematic literature review was conducted in a core medical journal, *Acupuncture in Medicine,* to identify qualified acupuncture case studies. The following exclusion criteria were applied: (1) absence of detailed case reports, (2) unspecified acupuncture point selection, and (3) use of nonneedling therapies (eg, cupping or moxibustion) [44]. *Acupuncture in Medicine* is a bimonthly peer-reviewed journal. As each issue typically contains 6-10 research papers, we manually screened all published papers (2023-June 2025) and assessed their eligibility according to the predefined exclusion criteria. Fourteen eligible case studies were identified, translated from English into Chinese (see Multimedia Appendix 1), and underwent rigorous bilingual review by 3 researchers, followed by verification by 2 medical professionals to ensure terminological precision and linguistic accuracy.

### Use ChatGPT and DeepSeek to Generate Acupoints Based on Journal Cases

Finally, to generate diagnostic and therapeutic plans, the 14 qualified cases (in both the original English and translated Chinese versions) were input into DeepSeek (DeepSeek-V3) and ChatGPT (GPT-5) in July 2025. For medical expert review, all AI outputs and the case reports were subsequently translated into Chinese. We selected ChatGPT and DeepSeek as the AI platforms for this study because ChatGPT is the most widely used AI assistant worldwide and demonstrates strong performance across a broad range of tasks, including brainstorming, writing, and analytical reasoning [45]. Its GPT-5 model is further designed to support domain-specific applications and complex problem-solving tasks [46]. In contrast, DeepSeek-V3 is a leading reasoning-focused open-source AI model developed in China. It uses a high-performing mixture-of-experts architecture with documented technical specifications, including 671 billion total parameters, 37 billion activated parameters per token, and training on 14.8 trillion high-quality tokens [47,48]. As TCM knowledge is deeply rooted in Chinese terminology, classical medical literature, and syndrome differentiation, comparing ChatGPT and DeepSeek provides an opportunity to examine whether differences in model architecture and linguistic environment influence acupuncture protocol generation.

Figures displaying the case prompts and the corresponding outputs from 2 AI models (ChatGPT-5 and DeepSeek-V3) for acupoint selection are provided in Multimedia Appendix 2. The full contents generated by DeepSeek and ChatGPT in different language contexts are presented in Multimedia Appendix 3.

### Medical Expert Assessment

In the medical expert review process, after familiarizing themselves with the 7 acupoint selection criteria, 9 senior TCM physicians—led by another senior physician with more than 30 years of clinical experience who was also 1 of the 3 physicians who initially validated the assessment framework—conducted a blind evaluation of the AI-generated outputs alongside 14 original case reports published in *Acupuncture in Medicine*. The 9 TCM physicians were different from the other 2 framework validators and were voluntarily recruited from the same hospital.

Using the 7 acupoint selection criteria, the experts independently evaluated 5 groups of treatment protocols for each case: the ChatGPT-generated Chinese output, the ChatGPT-generated English output, the DeepSeek-generated Chinese output, the DeepSeek-generated English output, and the journal physician-generated protocols (JCASE). The grading was conducted between July and August 2025. To avoid potential confounding effects associated with using the same account, the English and Chinese case inputs for both ChatGPT and DeepSeek were submitted under different usernames.

We ensured that all evaluating physicians had a consistent understanding of the 7 acupoint selection criteria. The clinical criteria for acupoint selection are presented in sections ranging from Local Point Selection to Acupoint Synergistic Effect.

### Local Point Selection

Local acupoint selection is one of the oldest and most intuitive strategies in acupuncture. Both classical literature and clinical practice have long emphasized the principle of selecting the acupoint where it hurts [49], that is, the point corresponding to the site of pain. Modern acupuncture research similarly identifies pressure points, trigger points, or acupoints adjacent to the lesion as the basis for local acupoint selection. For example, ST35 is commonly selected for the treatment of knee pain [50]. Therefore, consistent with traditional and contemporary clinical acupuncture practice, local point selection refers to the selection of acupoints located at or near the affected anatomical region, symptom site, or lesion location [10,27].

### Distal Point Selection

The technique of distant acupoint selection is grounded in meridian and tendon theories, which emphasize principles such as treating nearby areas by selecting distant points and reinforcing distant areas to affect nearby regions [51]. Traditional medical manuals and clinical schools have long highlighted the practical use of distant-point methods to regulate nearby areas, particularly through the selection of acupoints located along the same meridian. For example, LI4 (Hegu) is frequently used for the treatment of toothache [52]. Therefore, distal point selection refers to the selection of acupoints located away from the affected area but therapeutically connected through meridian theory and established acupuncture practice [14,27].

### Syndrome-Based Point Selection

The essence of syndrome differentiation in TCM resides in the dynamic integration and interpretation of symptoms to arrive at dialectical diagnostic conclusions [16]. This process is grounded in the four diagnostic methods—inspection, auscultation or olfaction, inquiry, and palpation—through which clinical information is systematically collected. By filtering, categorizing, and associating these symptoms within the theoretical frameworks of yin-yang (a TCM conceptual framework used to describe complementary and dynamically interacting aspects of physiological function) [53], the zang-fu organs (a collective term for the internal organs of human beings) [53], and the meridian system, practitioners can identify underlying pathophysiological patterns and formulate individualized treatment strategies [54], for example, LV3 for liver Qi (vital energy) stagnation [53]. Therefore, syndrome-based point selection refers to acupoint selection based on TCM syndrome differentiation, including the interpretation of symptoms, disease location, disease nature, and overall Zheng or pattern [21,23].

### Meridian-Tracing Point Selection

Meridians are a system of conduits through which Qi (vital energy) and blood circulate, linking the zang-fu organs, viscera, extremities, and superficial tissues, thereby integrating the body into an organic whole [10]. Over the years, the concept of meridians has been supported by researchers and clinicians through physical, chemical, and biological experimental evidence [55]. For example, GB20 (Fengchi) is commonly used to treat headaches along the gallbladder meridian. Therefore, meridian-tracing point selection refers to selecting acupoints according to the pathway of the involved meridian and its relationship with the affected region or organ system. It is also consistent with the WHO description of meridians and acupoint nomenclature [10,14].

### Neuroanatomical Point Selection

Aligning acupoints with anatomical and neurological knowledge involves selecting points based on the underlying peripheral nerves, dermatomes, muscles, or neuromuscular connections [56]. For example, GB30 (Huantiao) is commonly chosen for lower limb pain syndromes corresponding to the sciatic nerve root. Therefore, neuroanatomical point selection refers to selecting acupoints according to relevant biomedical structures, including peripheral nerves, dermatomes, muscles, or neuromuscular pathways [57,58].

### Core Acupoint Coverage Rate

Core acupoints are primary points that have been clinically demonstrated to exert central therapeutic effects for specific conditions, such as HT7 for insomnia and ST36 for immune modulation. This step is particularly important, as TCM practitioners may develop distinct treatment protocols influenced by their individual clinical experience. Nevertheless, there is notable convergence around 5 to 6 core acupoints that consistently appear across most protocols [59]. Therefore, core acupoint coverage refers to the extent to which the selected protocol includes commonly recognized primary acupoints for the corresponding condition, reflecting the importance of identifying frequently used and clinically central acupoints in acupuncture treatment patterns [60]. In our study, *Acupuncture and Moxibustion* [61] was used as the standard reference for identifying core acupoints.

### Acupoint Synergistic Effect

The acupoint synergistic effect refers to the phenomenon in which a combination of two or more acupoints produces a therapeutic outcome that exceeds the sum of the effects achieved by each acupoint individually [62]. Therefore, acupoint synergistic effect refers to the extent to which the selected acupoints form a coherent therapeutic combination rather than functioning as isolated points; this criterion is supported by studies showing that acupoint combinations may produce synergistic therapeutic effects and are central to acupuncture prescription design [60,63].

### Acupoint Antagonistic Effect

The antagonistic effect refers to a phenomenon in which one substance reduces or blocks the effect of another, commonly observed in pharmacology when an antagonist inhibits the action of an agonist [64]. However, the STRICTA (Standards for Reporting Interventions in Clinical Trials of Acupuncture) guidelines and the WHO acupuncture benchmark, while providing detailed reporting standards for point location and rationale, make no reference to “antagonistic effects.” This omission reflects the fact that the concept is not well established in evidence-based acupuncture research [10,27]. Most classical literature and modern clinical studies instead emphasize the synergistic effect between acupoints, such as the combination of Hegu (LI4) and Taichong (LR3) [65]. In contrast, reports of systematic antagonistic effects are virtually nonexistent. Therefore, given the lack of supporting evidence and the absence of authoritative standards, we ultimately removed this criterion from our assessment framework.

These 7 assessment criteria formed a standardized assessment framework for evaluating the acupoint selection protocols in our study. Ten physicians independently completed the assessments using paper-based evaluation forms incorporating the 7-criterion assessment framework. The corresponding statistical analysis results are presented in the Inferential Statistics Results subsection of the following Results section.

For each of the 14 acupuncture cases, the acupoint selection protocols generated by the AI platforms, together with the corresponding acupoint prescription reported in the original case (5 groups in total), were independently evaluated using a 5-point Likert scale based on 7 predefined assessment criteria. A sample evaluation question is as follows: (1) To what extent do the selected acupoints comply with the local point selection principle? Responses were rated on a five-point Likert scale (1-5), which was treated as interval data for the purpose of calculating mean scores and conducting statistical analyses: 5—to a very large extent, 4—to a large extent, 3—to a moderate extent, 2—to a small extent, and 1—to a very small extent. The remaining 6 items followed the same structure and evaluated the extent to which the selected acupoints conformed to the distal point selection principle, syndrome-based point selection principle, meridian-tracing point selection principle, neuroanatomical point selection principle, core acupoint coverage principle, and acupoint synergistic effects principle.

### Data Analysis

We conducted descriptive statistical analyses to assess content consistency. First, we examined whether ChatGPT or DeepSeek generated identical outputs when the same medical case was entered in different languages (Chinese and English) using different user accounts. Next, 2 independent input trials were conducted for each platform using the same cases in the same languages. The number of acupuncture points generated and the degree of overlap between the outputs were then evaluated. Content consistency is a critical metric for assessing the reproducibility of AI-generated outputs. If the same instructions produce different acupoint protocols under identical conditions (same platform, language, and case), it indicates variability in the AI’s responses, which may undermine the reliability and clinical interpretability of its recommendations.

Lastly, we conducted inferential analyses based on the expert evaluation data. Ten medical experts independently assessed the acupoint protocols generated by the AI platforms, as well as the original case-reported protocols, across 14 medical cases using 7 predefined evaluation criteria. As the same 10 raters, blinded to the source of each protocol, evaluated all 5 protocol groups [66], a repeated-measures ANOVA was considered appropriate to compare ratings across the 5 protocol groups. This repeated-measures design controls for individual differences among raters, such as a tendency to assign consistently higher or lower ratings, thereby increasing statistical power. For statistical analysis, case-level ratings were aggregated by calculating mean scores across the 14 cases for each of the 5 protocol groups.

To conduct the repeated-measures ANOVA analysis, we first assessed the normality of the differences between protocol groups (ChatGPT Chinese, ChatGPT English, DeepSeek Chinese, DeepSeek English, and JCASE) by examining the Shapiro-Wilk *P* values for each difference variable. Most *P* values were greater than .05, indicating that the normality assumption for the differences could be reasonably met. Before conducting pairwise comparisons for each criterion, the Mauchly test of sphericity was performed to verify that the assumption of sphericity was met. All *P* values were greater than .05, indicating that the variances of the differences could be assumed equal.

### Ethical Considerations

This study received approval from the Institutional Review Board at Northeast Agricultural University (#IRB-2502-0714). The research was conducted in accordance with established ethical principles for human participant research. All participants provided informed written consent and were informed of their right to withdraw from this study at any time. Participation was voluntary, and no financial or other incentives were provided. Strict measures were taken to protect the privacy and confidentiality of the collected data. Specifically, all study data were deidentified. Completed paper-based evaluation forms and transcribed electronic study data were securely stored, with access restricted to authorized members of the research team. All original case reports from *Acupuncture in Medicine* were fully anonymized before publication; no direct contact with human patients occurred, and no personally identifiable patient information was accessed, collected, or stored during this research.

## Results

### Assessment of Outcome Consistency for Different Language Inputs

We analyzed the number of acupuncture points generated by ChatGPT for 14 cases in both Chinese- and English-language environments, and for overlapping points between the two, calculating the average values for these 3 measures separately. On average, ChatGPT generated 10.71 acupuncture points in the English environment and 11.50 points in the Chinese environment, with an average of 4.57 overlapping points between the two.

For DeepSeek, we adopted the same approach. On average, DeepSeek generated 11.57 acupuncture points in the English environment and 10.43 points in the Chinese environment, with an average of 6.21 overlapping points between the two. Visualizations of the English and Chinese outputs, along with overlapping acupoints across 14 cases for ChatGPT and DeepSeek, are presented in Figures 1 and 2.

**Figure 1:**
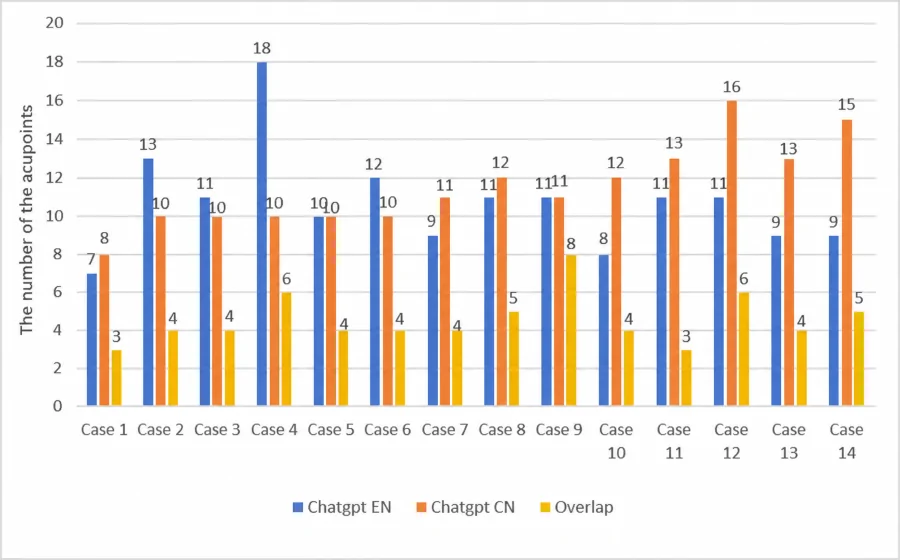
The number of acupoints generated by ChatGPT in different language environments. CN: Chinese; EN: English.

**Figure 2:**
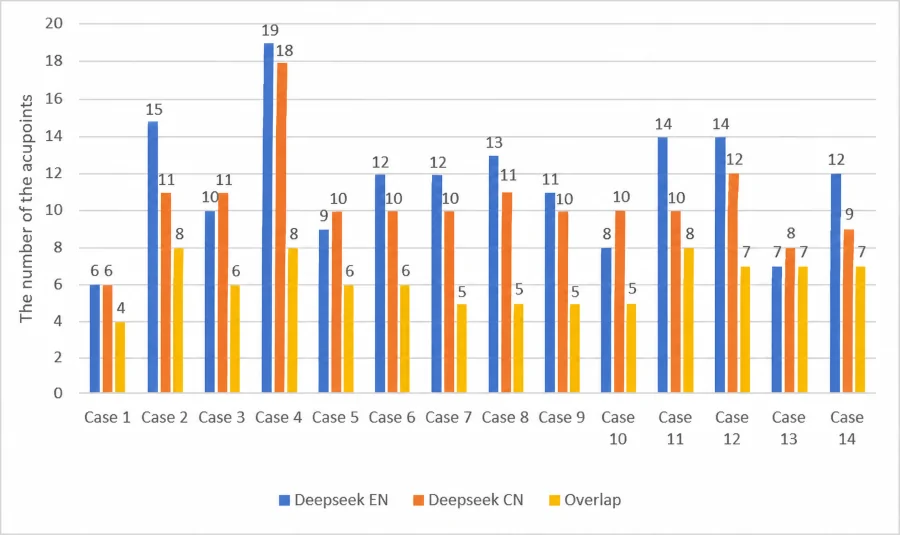
The number of acupoints generated by DeepSeek in different language environments. CN: Chinese; EN: English.

### Assessment of Outcome Consistency for Repeated Inputs

By evaluating overlaps across repeated inputs, we can determine whether the AI provided stable and consistent guidance, an essential requirement for trustworthy integration into medical decision-making.

Figures 3-6 present visualizations of the rounds 1 and 2 acupoint outputs and the overlapping acupoints between the 2 rounds across all 14 cases for ChatGPT English, ChatGPT Chinese, DeepSeek English, and DeepSeek Chinese.

**Figure 3:**
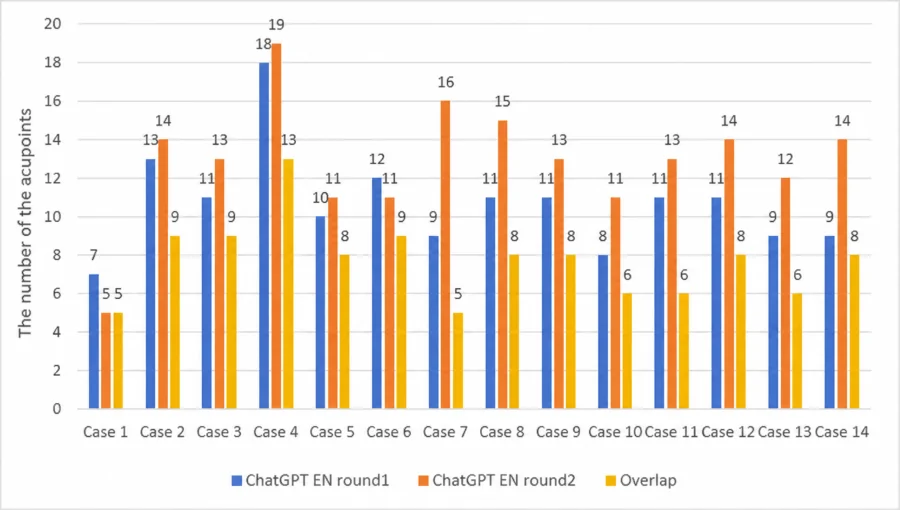
The number of acupuncture points generated by ChatGPT after 2 submissions of instructions using different accounts in English. CN: Chinese; EN: English.

**Figure 4:**
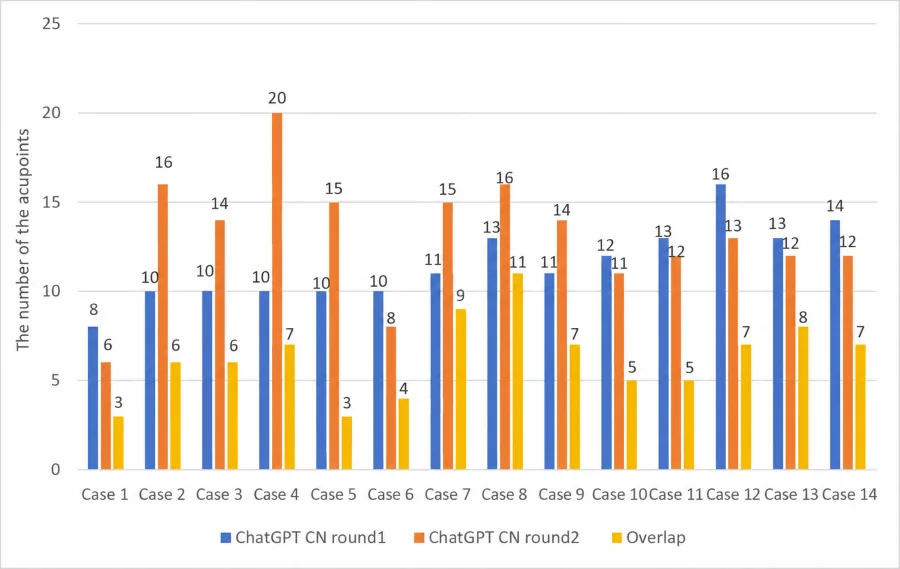
The number of acupuncture points generated by ChatGPT after 2 submissions of instructions using different accounts in Chinese. CN: Chinese; EN: English.

**Figure 5:**
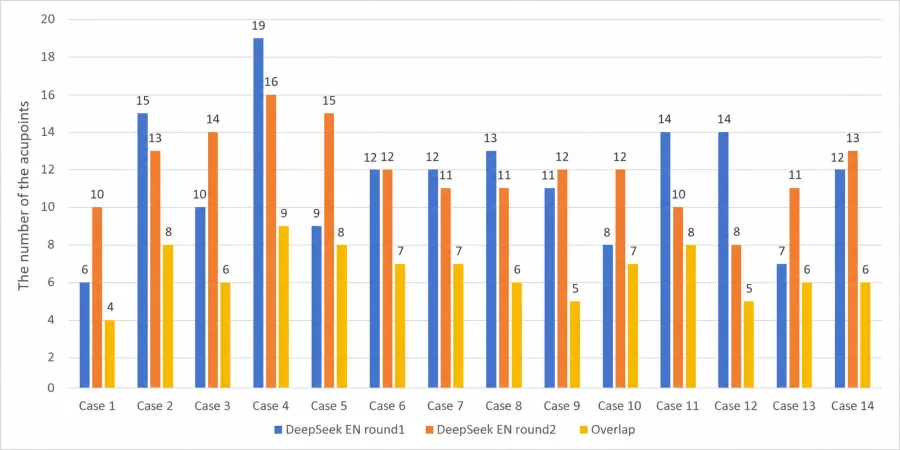
The number of acupuncture points generated by DeepSeek after 2 submissions of instructions using different accounts in English. CN: Chinese; EN: English.

**Figure 6:**
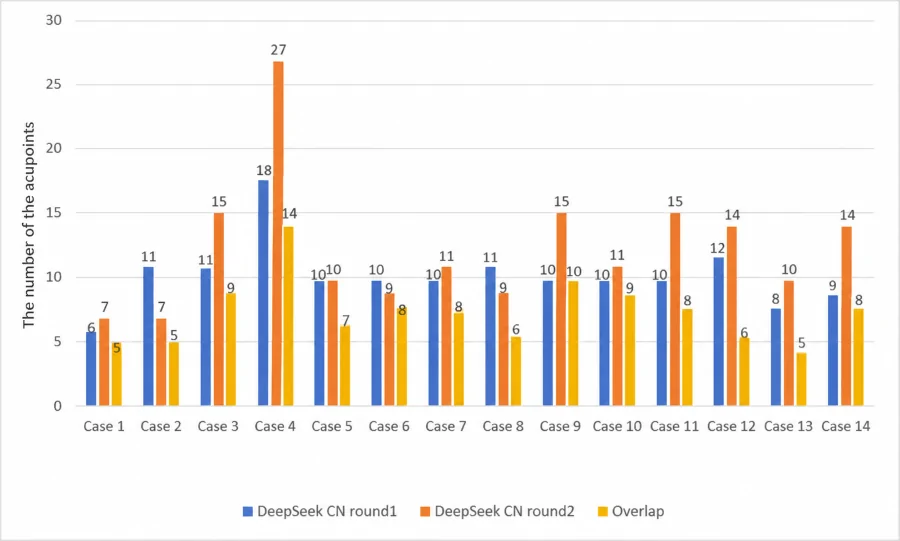
The number of acupuncture points generated by DeepSeek after 2 submissions of instructions using different accounts in Chinese. CN: Chinese; EN: English.

Based on 2 independent input trials across the 14 cases, ChatGPT English generated an average of 10.71 and 12.93 acupuncture points in rounds 1 and 2, respectively, with 7.71 overlapping points between rounds. Corresponding values were 11.50, 13.14, and 6.29 for ChatGPT Chinese; 11.57, 12.00, and 6.57 for DeepSeek English; and 10.43, 12.43, and 7.71 for DeepSeek Chinese. Overall, the findings indicate that all models showed moderate variability in acupuncture point generation and overlap, with differences observed across language settings. These results suggest that model type and language context influence the consistency of AI-generated acupuncture point selection.

### Inferential Statistics Results

We averaged the scores across the 14 cases and computed the mean and SD for each criterion across the 5 protocol groups (Table 1 and Figure 7).

**Table 1:** Mean and SD for each evaluation criterion across the 5 groups.

Criteria and group^a^		Score, mean (SD)	Rater, n
(1) To what extent do the selected acupoints comply with the local point selection principle?			
	Group 1	3.793 (0.488)	10
	Group 2	3.779 (0.566)	10
	Group 3	3.950 (0.524)	10
	Group 4	3.593 (0.544)	10
	Group 5	3.636 (0.504)	10
(2) To what extent do the selected acupoints comply with the distal point selection principle?			
	Group 1	3.171 (0.316)	10
	Group 2	3.257 (0.422)	10
	Group 3	3.257 (0.316)	10
	Group 4	3.371 (0.275)	10
	Group 5	3.121 (0.422)	10
(3) To what extent do the selected acupoints comply with the syndrome-based point selection principle?			
	Group 1	3.221 (0.557)	10
	Group 2	3.314 (0.524)	10
	Group 3	3.336 (0.474)	10
	Group 4	3.436 (0.494)	10
	Group 5	3.021 (0.618)	10
(4) To what extent do the selected acupoints comply with the meridian-tracing point selection principle?			
	Group 1	3.429 (0.480)	10
	Group 2	3.486 (0.398)	10
	Group 3	3.336 (0.549)	10
	Group 4	3.429 (0.415)	10
	Group 5	3.343 (0.490)	10
(5) To what extent do the selected acupoints comply with the neuroanatomical point selection principle?			
	Group 1	3.143 (0.552)	10
	Group 2	3.371 (0.616)	10
	Group 3	3.264 (0.635)	10
	Group 4	3.200 (0.558)	10
	Group 5	3.036 (0.591)	10
(6) To what extent are the core acupoints covered by the selected acupoints?			
	Group 1	3.364 (0.539)	10
	Group 2	3.479 (0.518)	10
	Group 3	3.386 (0.492)	10
	Group 4	3.314 (0.549)	10
	Group 5	3.179 (0.499)	10
(7) To what extent do the selected acupoints demonstrate synergistic effects?			
	Group 1	3.121 (0.612)	10
	Group 2	3.229 (0.658)	10
	Group 3	3.250 (0.568)	10
	Group 4	3.129 (0.567)	10
	Group 5	3.021 (0.545)	10

^a^Group 1: ChatGPT English; group 2: DeepSeek English; group 3: DeepSeek Chinese; group 4: ChatGPT Chinese; and group 5: journal physician-generated protocols.

**Figure 7:**
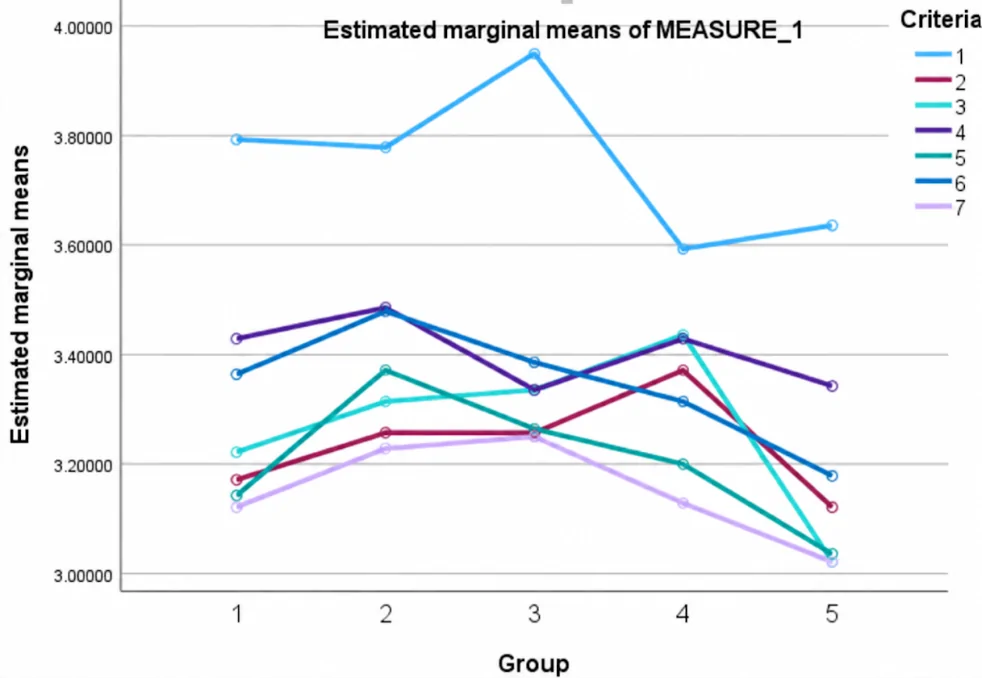
Estimated marginal means for each evaluation criterion across the 5 protocol groups. Group 1: ChatGPT English; group 2: DeepSeek English; group 3: DeepSeek Chinese; group 4: ChatGPT Chinese; and group 5: JCASE. JCASE: journal physician-generated protocols.

### Extent to Which the Selected Acupoints Comply With the Local Point Selection Principle

The repeated-measures ANOVA showed a significant effect of group on the ratings (*F*_4,36_=4.262, *P*=.006, partial η²=.321), indicating that the within-subject effects were statistically significant and there was a significant difference among the 5 groups’ mean ratings. All the post hoc pairwise comparisons were conducted using estimated marginal means with the least significant difference procedure. As shown in Table 2, group 1 (ChatGPT English) has significantly lower ratings than group 3 (DeepSeek Chinese) but higher than group 4 (ChatGPT Chinese). Group 3 (DeepSeek Chinese) has significantly higher ratings than groups 4 (ChatGPT Chinese) and 5 (JCASE). Due to page limitations, only outcomes showing significant relationships are presented in the main text; complete results are provided in Multimedia Appendix 4.

**Table 2:** Pairwise comparisons among 5 groups based on the local point selection principle^a^.

(I) group and (J) group		Mean difference (I-J)	SE	*P* value^b^	95% CI for difference^b^	
					Lower bound	Upper bound
1 CTEN^c^						
	3 DSCN^d^	–0.157^e^	0.056	.02	–0.284	–0.030
	4 CTCN^f^	0.200^e^	0.072	.02	0.037	0.363
3 DSCN						
	4 CTCN	0.357^e^	0.066	<.001	0.209	0.506
	5 JCASE^g^	0.314^e^	0.099	.01	0.089	0.539

^a^Based on estimated marginal means.

^b^Adjustment for multiple comparisons: least significant difference (equivalent to no adjustments).

^c^CTEN: ChatGPT English.

^d^DSCN: DeepSeek Chinese.

^e^The mean difference is statistically significant at the *P*=.05 level.

^f^CTCN: ChatGPT Chinese.

^g^JCASE: journal physician-generated protocols.

### Extent to Which the Selected Acupoints Comply With the Distal Point Selection

The repeated-measures ANOVA did not show a significant effect of group on the ratings (*F*_4,36_=2.039, *P*=.11, partial η²=0.185), indicating that the within-subject effects were not statistically significant and there was not a significant difference among the 5 groups’ mean ratings. Consequently, pairwise comparisons were not conducted. These results suggest that the AI-generated outputs and the JCASE did not differ significantly in terms of compliance with the distal point selection principle.

### Extent to Which the Selected Acupoints Comply With the Syndrome-Based Point Selection Principle

The repeated-measures ANOVA revealed a significant effect of group (*F*_4,36_=4.503, *P*=.005, partial η²=0.333), indicating that the within-subject effects were statistically significant and there was a significant difference among the 5 groups’ mean ratings. As shown in Table 3, group 2 (DeepSeek English), group 3 (DeepSeek Chinese), and group 4 (ChatGPT Chinese) are all significantly higher than group 5 (JCASE).

**Table 3:** Pairwise comparisons among 5 groups based on the syndrome-based point selection principle^a^.

(I) group	(J) group	Mean difference (I-J)	SE	*P* value^b^	95% CI for difference^b^	
					Lower bound	Upper bound
2 DSEN^c^	5 JCASE^d^	0.293^e^	0.124	.04	0.011	0.574
3 DSCN^f^	5 JCASE	0.314^e^	0.118	.026	0.047	0.582
4 CTCN^g^	5 JCASE	0.414^e^	0.123	.008	0.136	0.693

^a^Based on estimated marginal means.

^b^Adjustment for multiple comparisons: least significant difference (equivalent to no adjustments).

^c^DSEN: DeepSeek English.

^d^JCASE: journal physician-generated protocols.

^e^The mean difference is statistically significant at the *P*=.05 level.

^f^DSCN: DeepSeek Chinese.

^g^CTCN: ChatGPT Chinese.

### Extent to Which the Selected Acupoints Comply With the Meridian-Tracing Point Selection Principle

The repeated-measures ANOVA did not show a significant effect of group on the ratings (*F*_4,36_=1.213, *P*=.32, partial η²=0.119), indicating that the within-subject effects were not statistically significant and that there were no significant differences among the mean ratings of the 5 groups. Consequently, pairwise comparisons were not conducted. These results suggest that the AI-generated outputs and the JCASE did not differ significantly in terms of compliance with the meridian-tracing point selection principle.

### Extent to Which the Selected Acupoints Comply With the Neuroanatomical Point Selection Principle

The repeated-measures ANOVA revealed a significant effect of group (*F*_4,36_=6.649, *P*<.001, partial η²=0.425), indicating that the within-subject effects were statistically significant and there was a significant difference among the 5 groups’ mean ratings. As shown in Table 4, group 2 (DeepSeek English) is significantly higher than group 1 (ChatGPT English). Group 2 (DeepSeek English) is significantly higher than group 3 (DeepSeek Chinese), group 4 (ChatGPT Chinese), and group 5 (JCASE).

**Table 4:** Pairwise comparisons among 5 groups based on the neuroanatomical point selection principle^a^.

(I) group and (J) group		Mean difference (I-J)	SE	*P* value^b^	95% CI for difference^b^	
					Lower bound	Upper bound
1 CTEN^c^						
	2 DSEN^d^	–0.229^e^	0.042	<.001	–0.324	–0.133
2 DSEN						
	3 DSCN^f^	0.107^e^	0.044	.038	0.007	0.207
	4 CTCN^g^	0.171^e^	0.039	.002	0.084	0.259
	5 JCASE^h^	0.336^e^	0.078	.002	0.16	0.511

^a^Based on estimated marginal means.

^b^Adjustment for multiple comparisons: least significant difference (equivalent to no adjustments).

^c^CTEN: ChatGPT English.

^d^DSEN: DeepSeek English.

^e^The mean difference is statistically significant at the *P*=.05 level.

^f^DSCN: DeepSeek Chinese.

^g^CTCN: ChatGPT Chinese.

^h^JCASE: journal physician-generated protocols.

### Extent to Which the Selected Acupoints Covered the Core Acupoints

The repeated-measures ANOVA revealed a significant effect of group (*F*_4,36_=2.941, *P*=.03, partial η²=0.246), indicating that the within-subject effects were statistically significant and there was a significant difference among the 5 groups’ mean ratings. As shown in Table 5, group 1 (ChatGPT English), group 2 (DeepSeek English), and group 3 (DeepSeek Chinese) are all significantly higher than group 5 (JCASE).

**Table 5:** Pairwise comparisons among 5 groups based on the core points selection principle^a^.

(I) group	(J) group	Mean difference (I-J)	SE	*P* value^b^	95% CI for difference^b^	
					Lower bound	Upper bound
1 CTEN^c^	5 JCASE^d^	0.186^e^	0.065	.019	0.039	0.333
2 DSEN^f^	5 JCASE	0.300^e^	0.080	.005	0.118	0.482
3 DSCN^g^	5 JCASE	0.207^e^	0.079	.028	0.028	0.387

^a^Based on estimated marginal means.

^b^Adjustment for multiple comparisons: least significant difference (equivalent to no adjustments).

^c^CTEN: ChatGPT English.

^d^JCASE: journal physician-generated protocols.

^e^The mean difference is statistically significant at the *P*=.05 level.

^f^DSEN: DeepSeek English.

^g^DSCN: DeepSeek Chinese.

### Extent to Which the Selected Acupoints Demonstrate Synergistic Effects

The repeated-measures ANOVA did not show a significant effect of group on the ratings (*F*_4,36_=1.710, *P*=.17, partial η²=0.160), indicating that the within-subject effects were not statistically significant and that there were no significant differences among the mean ratings of the 5 groups. Consequently, pairwise comparisons were not conducted. These results suggest that the AI-generated outputs and the JCASE did not differ significantly with respect to the acupoint synergistic effects principle.

To summarize, regarding the local point selection principle, blinded expert evaluations showed that the DeepSeek Chinese output achieved significantly higher scores than the ChatGPT Chinese output (mean difference=0.357, *P*<.001), the ChatGPT English output (mean difference=0.157, *P*=.02), and the JCASE (mean difference=0.314, *P*=.01). ChatGPT English output performed better than the ChatGPT Chinese output (mean difference=0.200, *P*=.02). For the syndrome-based point selection principle, the JCASE scores were significantly lower compared with the DeepSeek Chinese output (mean difference=0.314, *P*=.026), DeepSeek English output (mean difference=0.293, *P*=.04), and ChatGPT Chinese output (mean difference=0.414, *P*=.008). In terms of the neuroanatomical point selection principle, the DeepSeek English output outperformed the ChatGPT English output (mean difference=0.229, *P*<.001), the ChatGPT Chinese output (mean difference=0.171, *P*=.002), the DeepSeek Chinese output (mean difference=0.107, *P*=.038), and the JCASE (mean difference=0.336, *P*=.002). For the core acupoint coverage principle, the JCASE scored significantly lower than the DeepSeek Chinese output (mean difference=0.207, *P*=.028), the DeepSeek English output (mean difference=0.300, *P*=.005), and the ChatGPT English output (mean difference=0.186, *P*=.019).

## Discussion

### Summary

Comparison of the analysis results indicates that DeepSeek generates more effective treatment protocols in the Chinese environment, whereas ChatGPT demonstrates superior performance in the English environment. This discrepancy reflects the language-specific strengths of the 2 AI models. Notably, 92% of the data used to train ChatGPT were in English [67], which likely contributes to its stronger performance in English-language tasks. In contrast, DeepSeek was trained on high-quality Chinese and English corpora, with a more pronounced ability to understand and generate Chinese content. These findings highlight the importance of considering the native language of AI models when applying them to cross-linguistic tasks, such as acupuncture point selection, as language proficiency may influence the accuracy and clinical relevance of generated treatment protocols.

We can infer that ChatGPT predominantly uses English-language data during training, whereas DeepSeek relies more heavily on Chinese-language data. Although AI tools have achieved human-level performance across various tasks, such outcomes are generally contingent upon the training and testing being conducted within the same linguistic context. For example, studies have shown that AI models extensively trained in English environments perform well on many tasks when evaluated in English, but exhibit significant limitations in cross-lingual transfer [9]. This linguistic dependency helps explain why medical treatment protocols generated by the 2 AI models perform better in their respective native-language environments.

It is noteworthy that, according to the *Neuroanatomical Point Selection* assessment criterion, the protocols generated by DeepSeek in the English environment outperform those produced by ChatGPT in both Chinese and English environments. This may be attributable to the substantial English-language anatomical content that DeepSeek likely encountered during training. Evidence from prior studies indicates that language models pretrained on domain-specific corpora, such as those in biomedicine and anatomy, demonstrate significantly improved performance in related tasks. For example, BioBERT shows that pretraining on biomedical corpora can substantially enhance BERT’s (Bidirectional Encoder Representations From Transformers) performance in tasks such as named entity recognition, relation extraction, and question answering [68]. Furthermore, cross-linguistic evaluations consistently reveal that models perform best in the language and domain with the most abundant training data, with English biomedical corpora being particularly extensive [69]. These findings suggest that DeepSeek may have been extensively exposed to English medical and anatomical materials during pretraining or fine-tuning, which could explain its precise use of anatomical terminology in generated English content.

The research results indicate that acupuncture treatment protocols formulated by clinical doctors received the lowest scores. This outcome likely reflects a mismatch between the implicit, experience-driven reasoning methods used in clinical practice and the explicit, recordable evidence emphasized by the 7-criterion assessment framework. As Michael Polanyi famously proposed, “We know more than we can express,” highlighting that tacit knowledge is perceivable and applicable yet cannot be fully articulated or codified [70]. In contrast, explicit knowledge can be conveyed through formal and systematic language [71]. Previous studies have demonstrated that tacit knowledge plays a critical role in the cognitive processes of medical professionals, yet much of this knowledge is difficult to capture fully in written medical records or procedural guidelines [72]. For instance, experienced doctors frequently rely on reasoning that is context-dependent and experiential, such as detecting acupoint reactions during palpation or integrating an overall syndrome impression during the Four Diagnostic Examinations—forms of on-site, implicit knowledge that are not easily codified.

This tacit knowledge is often not fully captured in medical records. In contrast, LLMs and AI tools tend to generate “textbook-like” prescriptions. They can integrate common patterns from numerous sources and express their reasoning in explicit, standardized language, which aligns closely with the evaluation criteria. Empirical studies have shown that LLMs can encode and reproduce clinical knowledge after appropriate training [72]. This helps explain why AI-generated protocols score higher on evaluation criteria that emphasize explicitness. Recent research in knowledge management and natural language processing has also highlighted that AI technologies can facilitate the externalization of professional tacit knowledge—converting experiential, practice-based insights into codifiable and shareable outputs [73]. It is important to emphasize that these findings do not suggest AI is superior to doctors in clinical judgment. Rather, they highlight AI’s practical advantage in externalizing implicit knowledge, enhancing documentation quality, and promoting repeatability and cross-language communication. In other words, AI can help transform the tacit reasoning of acupuncture practitioners into a visible and verifiable format, enabling acupuncture to better align with modern medical standards and standardization requirements.

As TCM emphasizes individualized treatment, syndrome-based point selection is widely regarded as the core approach in acupuncture therapy. Additionally, some studies have shown that for pain management, local point selection is used more frequently than other acupoint selection methods [74]. The comparative results indicated that AI-generated acupuncture protocols achieved higher scores in local point selection but relatively lower scores in syndrome-based point selection. Compared with the comprehensive considerations required for syndrome-based point selection, local acupuncture points follow a more explicit logic: “where the disease is, the point should be selected.” This straightforward rule is easier to standardize, record, and replicate, making it more aligned with explicit knowledge. As AI tools are particularly adept at encoding and reproducing explicit knowledge, the generated protocols correspond more closely with the local point selection method.

The discrepancy between ChatGPT- and DeepSeek-generated treatment protocols reflects the language-specific strengths of the 2 AI models. One possible explanation for these performance differences is variation in how TCM knowledge is represented across Chinese and English training corpora. TCM concepts such as Zheng, meridian theory, and syndrome-based treatment are more explicitly and precisely expressed in Chinese-language sources [42,43], whereas English biomedical resources rely more on standardized terminological systems and structured vocabularies [75]. Together, these factors suggest that performance differences may reflect differences in both language exposure and the underlying organization of domain-specific medical knowledge across languages.

### Theoretical Implication

First, our study clarifies and implements 7 independent criteria for acupoint selection, thereby advancing the development of TCM theory concretely. It transforms previously scattered clinical experiences into a repeatable, multidimensional 7-criterion assessment framework applicable across different cases and raters. This formalization not only complements existing standards for acupuncture trial reporting—which emphasize transparency and theoretical justification of interventions [27]—but also addresses an important methodological gap. Specifically, it provides a measurement tool focused on the reasoning behind acupoint selection, rather than merely documenting operational details. By doing so, this standard addresses the WHO’s ongoing efforts to standardize acupuncture point selection, enabling the previously experience-dependent selection process to be quantitatively evaluated. In summary, the scale represents a step toward integrating TCM diagnostic thinking with evidence-based evaluation, offering a framework for the quantification of clinical decisions in acupuncture.

Second, this research represents methodological innovation by providing a pathway to transform the implicit knowledge in acupuncture into explicit, assessable forms and conduct systematic evaluations. This study converts practitioners’ empirical judgments into explicit standards that can be scored and compared, making previously tacit insights visible to peers, educators, and researchers. The ability to externalize and quantify these judgments not only strengthens the theoretical foundation of TCM but also provides a reusable framework for identifying areas of agreement or divergence among expert practice, AI-generated outputs, and literature-based norms. By operationalizing acupoint selection into a repeatable, multidimensional evaluation criterion, this research enables certain knowledge traditionally implicit in acupuncture practice to be quantified and subjected to rigorous comparative analysis, thereby making a significant contribution to the broader understanding and modernization of TCM theory.

### Practical Implication

First, our study found that, under certain circumstances, ChatGPT and DeepSeek performed less effectively than other alternatives, whereas the physician-generated protocols reported in the journal cases provided an important clinical benchmark. For instance, under the local point selection principle and neuroanatomical point selection principle, ChatGPT performed less effectively in the Chinese-language setting than in the English language setting and was also outperformed by DeepSeek in both language settings. This highlights a key limitation of current LLMs: their performance is heavily influenced by the language composition of their training data. Models trained predominantly in one language may not generalize effectively to other linguistic contexts, particularly for specialized domains such as TCM. These findings underscore the importance for AI developers to strengthen multilingual training and incorporate diverse, domain-specific corpora to improve cross-linguistic performance. In practice, this also suggests that when deploying AI tools in clinical or research settings, careful consideration must be given to the model’s language environment and domain expertise. Otherwise, the outputs may be less reliable or accurate, potentially limiting their utility for nonnative language users. Moreover, this observation points to a broader challenge in AI development: balancing generalizability with domain and language specialization, particularly in knowledge-intensive fields such as medicine.

Second, the research results suggest that to enhance AI-assisted acupuncture planning, future models should be trained not only on standard acupuncture prescription data but also on a Chinese medical diagnostic corpus incorporating the four diagnostic methods. Existing studies indicate that AI applications based on the four diagnostic methods are active and rapidly evolving. For instance, recent bibliometric analyses show a significant growth trend in AI research focused on the four diagnostic methods, particularly in tongue image classification and symptom differentiation [76,77]. By deliberately integrating diagnostic knowledge from these areas during training, AI models could generate outputs that more closely align with the traditional treatment logic of acupuncture, thereby improving both accuracy and clinical relevance. Another study proposes a framework that integrates multimodal data inputs—such as tongue images, facial skin color, pulse signals, and patient questionnaires—through a feature fusion layer to map these diagnostic inputs to disease types [78]. If AI models are fine-tuned on such multimodal datasets, they could more accurately replicate the reasoning process of TCM, capture the implicit clinical judgments of practitioners, and translate them into clear, explicit recommendations. These enhancements would allow AI-assisted acupuncture point selection to better align with the expectations of clinical doctors and improve the credibility and applicability of the method in real-world clinical settings.

Third, a key direction for AI-assisted acupuncture is achieving a balance between personalized treatment plans and generalized treatment protocols. Core acupoints can be identified through big data mining, representing the most commonly effective points for specific conditions, while auxiliary acupoints can be selected based on individual patient symptoms to reflect syndrome-based diagnosis in TCM. This approach is analogous to the use of modifiers in *ICD* (*International Classification of Diseases*) coding, where the core diagnosis is supplemented with patient-specific details to guide individualized care. By integrating both generalized knowledge and individualized diagnostic information, AI can replicate the implicit reasoning of clinical practitioners, generate explicit recommendations, and produce treatment plans that are both evidence-based and tailored to the unique presentation of each patient. This strategy not only enhances clinical relevance and credibility but also promotes the standardization and modernization of TCM practices.

Fourth, our proposed acupoint selection assessment framework has potential clinical utility in both TCM education and clinical practice. In TCM education, the framework could serve as a structured training tool for students and trainees to systematically assess the clinical appropriateness, completeness, and consistency of acupoint-selection protocols, whether developed by AI systems or by human practitioners. This may help trainees develop critical evaluation skills and a more systematic approach to assessing acupuncture treatment protocols. In clinical practice, the framework could facilitate more consistent assessment of treatment plans and provide a common basis for comparing AI-generated recommendations with physician-developed prescriptions. In the future, the framework could be incorporated into electronic health record–based clinical decision-support workflows to support the structured evaluation and documentation of acupuncture treatment protocols. Such applications would require further validation in real-world clinical settings and should complement, rather than replace, professional clinical judgment.

### Limitations and Future Research

This study has limitations. The number of clinical cases included was relatively small. A limited sample size reduces the statistical power of the findings and makes it difficult to generalize the results to diverse patient populations and disease types. In acupuncture research, small samples are particularly problematic due to substantial variability in syndrome differentiation and treatment strategies. However, a similar sample size was used in the medical education research [44]. Additionally, all clinical cases were selected from a single academic acupuncture journal. Although the cases were peer-reviewed, they may not fully represent the diversity of acupuncture practices, patient populations, and clinical conditions across different sources. Consequently, the generalizability of the findings should be interpreted with caution. Nevertheless, this sampling approach is consistent with prior medical education research that also relied on a single journal [44]. Future studies should incorporate cases from multiple journals to improve external validity.

Another potential limitation of this study is that it focused on the clinical appropriateness of generated acupuncture protocols rather than the underlying reasoning mechanisms of the AI models. Consequently, this study does not fully separate the effects of language environment and language generation capabilities from clinical reasoning processes. Future research could use methodologies specifically designed to distinguish linguistic proficiency from diagnostic and therapeutic decision-making performance.

### Conclusions

This study has established criteria for evaluating the effectiveness of acupuncture point selection. These criteria transform previously implicit clinical reasoning into quantifiable comparison indicators, enabling direct comparisons between real-world acupuncture treatment plans and AI-generated outputs. Our findings indicate that generative AI has demonstrated promising capability in acupuncture point selection, particularly in learning and reproducing explicit knowledge. However, AI limitations remain in handling more nuanced knowledge, such as treatment methods that require tailoring to individual cases, and in cross-linguistic consistency. These results further suggest that model performance varies across linguistic settings, highlighting the importance of language context in acupuncture protocol generation. This study also suggests that generative AI may contribute to knowledge standardization, education, and dissemination in TCM while emphasizing the need for further collaboration between TCM specialists and AI developers to explore domain-adapted, TCM-focused AI systems and modules. Future work should also consider cross-linguistic training strategies and the development of bilingual or multilingual corpora to improve LLM robustness across language settings.

## Data Availability

All protocols were evaluated by blinded domain experts, and the corresponding scores are provided in Multimedia Appendix 5.

## Appendix

Multimedia Appendix 1 Fourteen cases in English and Chinese.

Multimedia Appendix 2 Sample screenshots of the results generated by the 2 types of AI in different language environments.

Multimedia Appendix 3 The complete contents generated by AI.

Multimedia Appendix 4 Complete statistical table.

Multimedia Appendix 5 Expert assessment score.

## Abbreviations

BERT: Bidirectional Encoder Representations From Transformers
BFA: battlefield acupuncture
CAM: complementary and alternative medicine
ICD: International Classification of Diseases
ICD-11: International Classification of Diseases, 11th Revision
JCASE: journal physician-generated protocols
LLM: large language model
STRICTA: Standards for Reporting Interventions in Clinical Trials of Acupuncture
TCM: traditional Chinese medicine
TM: traditional medicine
USMLE: United States Medical Licensing Examination
WHO: World Health Organization
