# Supplementary material for: Comparative Evaluation of ChatGPT, DeepSeek, and Physician-Generated Acupuncture Treatment Protocols Across Chinese and English Language Settings: Cross-Sectional Evaluation Study
Source: JMIR Form Res. 2026 Oct 8;10:e95851. doi: 10.2196/95851 (PMC13691461; doi:10.2196/95851)
Supplement: Supplementary file 1 [file formative_v10i1e95851_app1.docx]

English Input

1

Please suggest the acupuncture points for the following case presentation, "On 24 September 2022, a 23-year-old male patient visited the Acupuncture and Massage Department of Affiliated Sport Hospital of Chengdu Sport University, complaining of discomfort and pain in both lower limbs for the preceding 2 years. The symptoms were significantly worse at night and relieved by moving and walking. Based on diagnostic criteria, the doctor in the Acupuncture and Massage Department diagnosed the patient with RLS. Due to concerns about potential adverse effects of medication and the patient’s physical condition, the patient was unwilling to receive medication. Therefore, at the suggestion of the doctor, the patient underwent acupuncture treatment."

2

Please suggest the acupuncture points for the following case presentation, "In February 2023, a 79-year-old female patient came to our hospital complaining of neck and shoulder pain and an unstable gait. She reported pain in her neck and shoulder, numbness and pain in her left upper limb, a tendency to fall while walking and weakness in both lower limbs. She described feeling like she was ‘walking on cotton’. The physical examination revealed stiffness in the neck muscles, tenderness, a positive neck compression test and weakened biceps and triceps reflexes on the left side with positive Hoffmann’s and Babinski’s signs. Computed tomography (CT) and magnetic resonance imaging (MRI) of the cervical spine showed calcification of the posterior longitudinal ligament at the posterior margins of the cervical vertebrae at C4, C5 and C6, leading to compression of the dural sac and the left C6 nerve root, with disc protrusion between the cervical vertebrae at C5 and C6. The patient was diagnosed with C-OPLL and surgical intervention was recommended. Despite this, the patient strongly declined surgery and opted instead for acupuncture treatment at our hospital."

3

Please suggest the acupuncture points for the following case presentation, "In 2022, a 66-year-old female patient presented with complaints of generalized muscle weakness and soreness for over 10 years, aggravated by severe tearing pain in the limbs for the past 3 months. The patient was diagnosed with PM after a relevant clinical examination, with her serum creatine kinase (CK) levels peaking at around 900 U/L. She had received treatment with medications including prednisone and celecoxib, which had improved her condition to some extent; however, she often relapsed. Three months prior, after catching a cold, the patient had again experienced muscle weakness throughout her body, accompanied by intense tearing pain in the limbs (especially the lower limbs). Due to her advanced age and fear of adverse drug reactions, the patient no longer wanted to undergo medical treatment. Therefore, upon the doctor’s recommendation, she chose a trial of acupuncture to potentially alleviate her muscle-related symptoms."

4

Please suggest the acupuncture points for the following case presentation, "A 57-year-old man was admitted to the hospital with limb weakness and gait instability symptoms that had persisted for 78 days. Physical examination showed that he had concealed aspiration, a weakened gag reflex, bilateral arm and leg weakness (graded as 5−on the left and 4+ on the right), prominent ataxia, disordered proprioception of the right upper and lower limbs, and mild dysarthria. His Fugl-Meyer assessment (FMA) scale, Barthel index (BI) and mini-mental state examination (MMSE) scores were 96, 80 and 26, respectively. His Kubota’s water swallow test was judged as profile III out of V (score = 4 out of 8), meaning that the patient was able to drink 30 mL of warm water within 5–10 s but with choking (defined as elicitation of a cough). A T2-weighted MRI showed high signal intensity of the bilateral cerebral peduncules, pons and bilateral thalami, as well as a positive ‘Mickey Mouse ear sign’. Magnetic resonance angiography (MRA) showed severe stenosis/occlusion of the bilateral intracranial vertebral artery (VA) and inferior basilar artery (IBA), and cerebroangiography confirmed severe BA stenosis and VA occlusion."

5

Please suggest the acupuncture points for the following case presentation, "A 42-year-old male patient suddenly developed left limb weakness accompanied by blurred vision in both eyes in August 2022 and was admitted to the Shaw Hospital. He was diagnosed with posterior circulation cerebral infarction and was discharged after treatment with dipyridamole to improve circulation, atorvastatin to reduce blood lipids, and other treatments to stabilize plaques and facilitate simple limb rehabilitation training. However, 1 month later, he felt that his symptoms of blurred vision and reduced visual fields had not improved and may even have worsened. He visited the ophthalmology department of the Second Affiliated Hospital of Zhejiang University and was diagnosed with homonymous hemianopia after a visual field examination. The doctor told him that there was no specific medication that could significantly improve this symptom and suggested that he undergo rehabilitation training. On 13 September 2022, he came to our hospital for acupuncture treatment and was diagnosed with post-stroke hemianopia."

6

Please suggest the acupuncture points for the following case presentation, "A 23-year-old Japanese male presented to Tokyo Ariake University of Medical and Health Sciences acupuncture clinic. He was complaining of pain in the right temporomandibular area and difficulty in opening his mouth for the past 2 months. His pain intensity and frequency had been gradually increasing, especially when he opened his mouth. The pain was exacerbated with eating and talking. The right temporalis and masseter muscles and the trapezius and splenius capitis muscles on both sides were tender to palpation."

7

Please suggest the acupuncture points for the following case presentation, "A 38-year-old man with AGA was managed as an outpatient. His chief complaint was hair loss for >3 years. He had been receiving treatment of AGA for >3 years since being diagnosed with AGA at the age of 35. During that time he had been going to a community hospital where finasteride tablets had been prescribed, but had not been effective. His manifestations included hair loss, fine hair (that tended to get flat and greasy), an itchy scalp and fatigue."

8

Please suggest the acupuncture points for the following case presentation, "A 33-year-old man with AGA was also managed as an outpatient. His chief complaint was hair loss for >2 years. He had been diagnosed with AGA at the age of 31 years but had never received treatment. There was a marked increase in hair loss on the top of his head, which had been getting worse day by day for the preceding 2 years. His manifestations included hair loss, itchy scalp, fatigue and anorexia."

9

Please suggest the acupuncture points for the following case presentation, "A 51-year-old woman who loves to climb was diagnosed with PFA in 2022. The patient had been complaining of left knee joint pain (mainly lateral left knee and subpatellar pain) and limited joint mobility for 1 year, which was obvious when squatting and going up and down stairs. The patellofemoral abrasion test was positive and there was knee space tenderness. She had been taking oral non-steroidal anti-inflammatory drugs and, while her symptoms sometimes improved, they often recurred. In June 2022, she underwent a magnetic resonance imaging (MRI) scan that revealed cartilage damage/degeneration in the left PFJ, subchondral bone marrow edema (BME), thickening of the synovial membrane and joint effusion of the left knee, and slight swelling of the surrounding soft tissues. Over the past year, left knee joint pain had impaired her normal life."

10

Please suggest the acupuncture points for the following case presentation, "On 5 March 2023, a 25-year-old female patient visited the Osteoarthropathy Department of the Affiliated Sport Hospital of Chengdu Sport University, complaining of recurrent instability of the left ankle joint for 1 year. Based on diagnostic criteria, the doctor in the Osteoarthropathy Department diagnosed the patient with FAI. As the patient did not have the time to undergo systematic rehabilitation, they chose to undergo acupuncture treatment at the suggestion of the doctor.“

11

Please suggest the acupuncture points for the following case presentation, "The day after testing positive for COVID-19, a 28-year-old female was suddenly unable to hear anything in her right ear. Her acute loss of hearing was accompanied by tinnitus (sounding like a cicada) in the same ear. She sought medical help at the local hospital and was diagnosed with SSNHL and tinnitus. After receiving oral methylprednisolone for 9 days (30 mg/day for the first 3 days, 20 mg/day for the next 3 days, followed by 10 mg/day for the last 3 days) and four tympanic injection of dexamethasone, no significant treatment effect was experienced. This came as a huge shock to her, especially as on discharge she was counseled that there was only a faint chance of her hearing improving. Thereafter, she was introduced to our hospital for acupuncture. Her pure tone audiometry of her right ear exhibited significant impairment upon admission.”

12

Please suggest the acupuncture points for the following case presentation, "A 68-year-old male patient with a previous history of hypertension presented on 27 May 2021 with a chief complaint of epigastric discomfort. Needle aspiration biopsy of the liver revealed a hypofractionated adenocarcinoma of pancreatic origin. A seven-cycle chemotherapy regimen of albumin paclitaxel combined with gemcitabine was started on 28 May 2021, with the last chemotherapy session taking place on 12 October 2021. Weakness was observed after chemotherapy, and blood work revealed a white blood cell count of 2.96 × 109/L and platelet count of 60 × 109/L, reflecting post-chemotherapy bone marrow suppression. The condition was suspected to have progressed following an increase in epigastric pain. Positron emission tomography computed tomography in January 2022 showed abnormal concentration of the radioactive isotope in the pancreas, suggesting increased local metabolism consistent with malignancy. Similar findings were observed in the liver, peritoneum, mesentery, pelvic fascia and terminal rectum, suggesting metastasis. Targeted anti-tumor therapy (erlotinib hydrochloride tablets 0.15 g daily combined with tegafur capsules 60 mg twice daily D1–D14) was commenced on 31 January 2022. He continued treatment for epigastric pain from 22 February 2022 to 24 April 2022. The patient’s last stage of survival (i.e. final hospitalization period from 1 to 24 April 2022) was dominated by cancer pain, which interfered with sleep and food intake. Strong opioids were considered according to the three-step analgesic rule.1 He received a continuous intravenous infusion of morphine hydrochloride (20 mg/h) as well as morphine sulfate extended-release tablets every 12 h and subcutaneous morphine hydrochloride injections (10 mg each) as required, according to the pain score. At the request of the patient’s family, acupuncture was used to try and alleviate the patient’s nausea, vomiting and other side effects of morphine.“

13

Please suggest the acupuncture points for the following case presentation, "Between 1 April 2021 and 31 March 2022, five patients with PPUI were treated at the First Teaching Hospital of Tianjin University of Traditional Chinese Medicine. The patients were aged 67.8 ± 5.54 years (mean ± SD) and had undergone a radical prostatectomy between 11 June 2018 and 15 January 2021. All these patients presented with a chief complaint of spontaneous leakage of urine during activity, laughing, sneezing or coughing for >12 months. Compared with 3 months following the surgery, the patient’s self-reported symptoms had improved, but they were all still experiencing problems with urinary leakage. Due to their advanced age and associated medical comorbidities, these patients had declined more invasive treatments.”

14

Please suggest the acupuncture points for the following case presentation, "In February 2023, a 38-year-old female patient presented at the First Affiliated Hospital of Henan University of Traditional Chinese Medicine. For the preceding 6 years, she had been experiencing recurrent episodes of rotational vertigo accompanied by symptoms such as nausea, photophobia and phonophobia lasting for several hours. In addition, she reported experiencing migrainous headaches in the right temporal region over the preceding year, although the severity of the headaches was not significant. It is worth noting that the vertigo and headaches seldom occurred simultaneously. The patient received a diagnosis of VM. She had been taking sodium valproate for a period of 6 months, which had resulted in a decrease in the frequency of vertigo episodes but was also accompanied by a weight gain of 8 kg. Six months prior to her visit, the patient had discontinued sodium valproate and had subsequently experienced frequent vertigo episodes, with approximately 7–8 episodes occurring each month. Seeking acupuncture treatment, she presented for medical care at our hospital.“

Chinese Input

1

请根据以下病例描述给出相应的针灸穴位建议：“2022年9月24日，一名23岁的男性患者就诊于成都体育学院附属体育医院针灸推拿科，主诉双下肢不适、疼痛2年，夜间症状明显加重，活动行走后症状缓解。针灸推拿科医师根据诊断标准，诊断患者为不宁腿综合征。由于患者担心药物可能产生的副作用及自身体质，不愿接受药物治疗，故在医师建议下接受针灸治疗。”

2

请根据以下病例描述给出相应的针灸穴位建议：“2023年2月，一位79岁的女性患者因颈肩疼痛和步态不稳来我院就诊。她主诉颈肩疼痛，左上肢麻木疼痛，行走时容易跌倒，双下肢无力。她描述感觉像“走在棉花上”。体格检查发现颈部肌肉僵硬、压痛，颈部压迫试验阳性，左侧二头肌和三头肌反射减弱，霍夫曼征和巴宾斯基征阳性。颈椎CT及MRI检查结果显示，C4、C5、C6颈椎后缘后纵韧带钙化，导致硬膜囊及左侧C6神经根受压，C5、C6颈椎间盘突出于C5、C6颈椎之间。患者被诊断为颈椎后纵韧带骨化症，并建议手术治疗。尽管如此，患者仍坚决拒绝手术，选择在我院接受针灸治疗。“

3

请根据以下病例描述给出相应的针灸穴位建议：“2022年，一名66岁的女性患者，主诉全身肌肉无力、酸痛十余年，近3个月来四肢剧烈撕裂痛加剧。经相关临床检查，患者被诊断为多发性骨髓瘤，血清肌酸激酶水平最高达到900 U/L左右。患者曾接受泼尼松、塞来昔布等药物治疗，病情有所改善，但经常复发。3个月前，患者感冒后再次出现全身肌肉无力，并伴有四肢（尤其是下肢）剧烈撕裂痛。由于年事已高，且担心药物不良反应，患者不愿再接受治疗。因此，在医生的建议下，她选择尝试针灸，以期缓解她的肌肉相关症状。”

4

请根据以下病例描述给出相应的针灸穴位建议：“患者，57岁，男性，因肢体无力、步态不稳症状持续了78天而入院。体格检查发现，患者有隐匿性误吸，咽反射减弱，双侧上肢和下肢无力（左侧5-，右侧4+），共济失调明显，右侧上下肢本体感觉障碍，轻度构音障碍。Fugl-Meyer功能评定量表评分为96分，Barthel指数（BI）评分为80分，简易精神状态检查表（MMSE）评分为26分。久保田水吞咽试验为Ⅲ级型（共V级型，得4分，共/8分），患者能在5～10秒内饮下30 mL温水，但有呛咳症状（定义为诱发咳嗽）。 MRI T2加权像显示双侧大脑脚、脑桥及双侧丘脑信号增强，并可见阳性“米老鼠耳征”。磁共振血管造影（MRA）显示双侧颅内椎动脉（VA）和下基底动脉（IBA）重度狭窄/闭塞，脑血管造影证实基底动脉BA重度狭窄及椎动脉VA闭塞。“

5

请根据以下病例描述给出相应的针灸穴位建议：“一名42岁的男性患者于2022年8月突发左侧肢体无力，伴双眼视物模糊，入住逸夫医院，诊断为后循环脑梗死，予双嘧达莫改善循环、阿托伐他汀降血脂等稳定斑块及进行简单的肢体康复训练等治疗后出院。但1个月后，患者感到视物模糊、视野缩小症状未见好转，甚至可能加重。就诊于浙江大学附属第二医院眼科，视野检查后诊断为同向性偏盲。医生告知，目前尚无特效药物能明显改善此症状，建议进行康复训练。2022年9月13日，患者来我院接受针灸治疗，诊断为中风后偏盲。“

6

请根据以下病例描述给出相应的针灸穴位建议：“一名23岁的日本男性患者到东京有明医科大学针灸诊所就诊。他主诉过去2个月内右侧颞下颌区域疼痛，并出现张口困难。疼痛强度和频率逐渐增加，尤其在张口时。进食和说话时疼痛加剧。右侧颞肌、咬肌以及两侧斜方肌和头夹肌触痛。”

7

请根据以下病例描述给出相应的针灸穴位建议：“一名38岁的雄激素性脱发男性患者于门诊就诊。他的主诉是脱发超过3年。自35岁确诊雄激素性脱发以来，他已接受治疗超过3年。在此期间，他一直在一家社区医院就诊，并在那里服用非那雄胺片，但效果不佳。他的症状包括脱发、头发细软（容易变得扁平油腻）、头皮发痒、以及疲劳。“

8

请根据以下病例描述给出相应的针灸穴位建议：“一名33岁的雄激素性脱发男性患者也作为门诊患者就诊。他的主诉是脱发超过2年。他于31岁时被诊断患有雄激素性脱发，但从未接受过治疗。在过去的2年里，他的头顶脱发明显增加，并且日益加重。他的临床表现包括脱发、头皮瘙痒、疲劳和厌食。“

9

请根据以下病例描述给出相应的针灸穴位建议：“一名51岁的女性，爱好爬山，于2022年被诊断患有PFA（髌骨关节综合征）。患者主诉左膝关节疼痛（主要为左膝外侧及髌骨下疼痛）及关节活动受限1年，以下蹲和上下楼梯时明显。髌股磨损试验阳性，膝关节间隙压痛。患者一直服用口服非甾体类抗炎药，症状有时有所改善，但经常复发。2022年6月，患者接受磁共振成像扫描，结果显示左侧PFJ髌骨关节软骨损伤/退变，软骨下骨髓水肿，左膝滑膜增厚及关节积液，周围软组织轻度肿胀。近一年来，左膝关节疼痛一直影响着患者的正常生活。”

10

请根据以下病例描述给出相应的针灸穴位建议：“2023年3月5日，一名25岁的女性患者就诊于成都体育学院附属体育医院骨关节病科，主诉左踝关节反复不稳定1年。骨关节病科医生根据诊断标准，诊断患者为关节功能障碍（FAI）。由于患者无时间进行系统康复治疗，在医生建议下选择针灸治疗。“

11

请根据以下病例描述给出相应的针灸穴位建议：“一名28岁的女性在新冠病毒检测呈阳性的第二天，突然右耳听不见任何声音。她的急性听力丧失伴有耳鸣（类似蝉鸣）。她在当地医院就诊，被诊断为突发性耳聋（SSNHL）。在接受9天口服甲泼尼龙（前3天30毫克/天，后3天20毫克/天，最后3天10毫克/天）和4次鼓膜注射地塞米松后，治疗效果不佳。这让她非常震惊，尤其是在出院时，她被告知听力恢复的可能性很小。此后，她被介绍到我院接受针灸治疗。入院时，她的右耳纯音听力检查结果显示明显受损。”

12

请根据以下病例描述给出相应的针灸穴位建议：“患者男，68 岁，既往有高血压病史，于 2021 年 5 月 27 日因上腹部不适主诉就诊。肝脏针吸活检显示胰腺低分化腺癌。2021 年 5 月 28 日开始白蛋白紫杉醇联合吉西他滨七周期化疗方案，末次化疗于 2021 年 10 月 12 日进行。化疗后患者出现乏力，血液检查显示白细胞计数为 2.96 × 109/L，血小板计数为 60 × 109/L，提示化疗后骨髓抑制。患者上腹部疼痛加剧，怀疑病情进展。2022 年 1 月正电子发射断层扫描 (PET) 显示胰腺放射性同位素浓度异常，提示局部代谢增加与恶性肿瘤相符。肝脏、腹膜、肠系膜、盆腔筋膜和末端直肠均观察到类似表现，提示有转移。患者于2022年1月31日开始接受靶向抗肿瘤治疗（盐酸厄洛替尼片0.15 g/d，联合替加氟胶囊60 mg/d，每日两次，第1至第14天）。患者于2022年2月22日至2022年4月24日继续治疗上腹部疼痛。患者生存期的最后阶段（即最终住院期，2022年4月1日至24日）以癌痛为主，影响睡眠和进食。根据三级镇痛规则，考虑使用强效阿片类药物。患者持续静脉输注盐酸吗啡（20 mg/h），每12小时服用一次硫酸吗啡缓释片，并根据疼痛评分按需皮下注射盐酸吗啡（每次10 mg）。应患者家属要求，采用针灸疗法，尝试缓解患者的恶心、呕吐及其他吗啡副作用。“

13

请根据以下病例描述给出相应的针灸穴位建议：“2021年4月1日至2022年3月31日，天津中医药大学第一附属医院收治了5例前列腺切除术后尿失禁患者。患者年龄67.8±5.54岁（平均值±标准差），于2018年6月11日至2021年1月15日期间接受了根治性前列腺切除术。所有患者均以活动、大笑、打喷嚏或咳嗽时自发性漏尿为主诉，且持续超过12个月。与术后3个月相比，患者自述症状有所改善，但仍然有漏尿问题。由于高龄及合并症，这些患者拒绝接受更具侵入性的治疗。”

14

请根据以下病例描述给出相应的针灸穴位建议：“2023年2月，一名38岁的女性患者就诊于河南中医学院第一附属医院。此前6年，患者反复发作旋转性眩晕，并伴有恶心、畏光、畏声等症状，每次持续数小时。此外，患者自述过去一年内出现右颞区偏头痛，但头痛程度不显著。值得注意的是，眩晕和头痛很少同时发生。患者被诊断为前庭性偏头痛（VM）。患者服用丙戊酸钠6个月，眩晕发作频率有所下降，但体重也增加了8公斤。就诊前6个月，患者停用丙戊酸钠，此后眩晕发作频繁，每月约7-8次。患者因寻求针灸治疗而来我院就诊。“
