# Supplementary material for: Comparative Evaluation of ChatGPT, DeepSeek, and Physician-Generated Acupuncture Treatment Protocols Across Chinese and English Language Settings: Cross-Sectional Evaluation Study
Source: JMIR Form Res. 2026 Oct 8;10:e95851. doi: 10.2196/95851 (PMC13691461; doi:10.2196/95851)
Supplement: Supplementary file 2 [file formative_v10i1e95851_app2.docx]

**Figure S1.** ChatGPT’s output for acupuncture point selection with input text of case presentation (Chinese version)
.

 **Figure S2.** Deepseek’s output for acupuncture point selection with input text of case presentation (English version).

(ChatGPT’s output in English and Deepseek’s output in Chinese are omitted.)
