# Supplementary material for: Comparative Evaluation of ChatGPT, DeepSeek, and Physician-Generated Acupuncture Treatment Protocols Across Chinese and English Language Settings: Cross-Sectional Evaluation Study
Source: JMIR Form Res. 2026 Oct 8;10:e95851. doi: 10.2196/95851 (PMC13691461; doi:10.2196/95851)
Supplement: Supplementary file 3 [file formative_v10i1e95851_app3.docx]

DeepSeek EN

**Case 1**

Of course. Based on the provided case presentation of a 23-year-old male diagnosed with Restless Legs Syndrome (RLS), here is a detailed suggestion for acupuncture point selection, following the principles of Traditional Chinese Medicine (TCM) diagnosis and modern acupuncture research.

**Important Disclaimer:** This is for educational and informational purposes only. Acupuncture is a medical practice that must be performed by a qualified and licensed practitioner. A real-world treatment would involve a comprehensive TCM diagnosis (including tongue and pulse examination) to tailor the points perfectly. This suggestion is based on the standard TCM approach to such a presentation.

---

\### TCM Pattern Differentiation

Based on the symptoms:

*	**Discomfort and pain in both lower limbs:** Indicates a problem in the channels of the legs, primarily the Liver, Spleen, and Kidney systems, as well as the Stomach and Bladder channels.

*	**Worse at night:** Nighttime is related to Yin. Worsening at night often suggests a **Yin deficiency** (e.g., Liver or Kidney Yin deficiency) where deficient Yin cannot nourish the tendons and vessels, leading to internal wind and restlessness.

*	**Relieved by moving and walking:** This is a classic sign of **Qi stagnation** or **Blood stasis**. Movement helps to move Qi and Blood, temporarily alleviating the stagnation and thus the discomfort.

*	**Long duration (2 years):** A chronic condition often involves a deficiency (of Qi, Blood, Yin, or Yang) alongside a pathogenic factor (like stasis or stagnation).

A very common TCM pattern for RLS is **Liver Blood Deficiency leading to Internal Wind**, often combined with **Qi and Blood Stasis**.

\### Suggested Acupuncture Points

The treatment strategy should aim to: **Nourish Blood and Yin, Soothe the Liver, Extinguish Wind, Promote Qi and Blood circulation, and Calm the Spirit.**

\#### 1. Core Local Points (To regulate Qi and Blood in the limbs):

*	**ST 36 (Zusanli):** The He-Sea point of the Stomach channel. **Tonifies Qi and Blood** overall, strongly benefits the lower limbs, and regulates the Stomach and Spleen to aid in the production of Qi and Blood.

*	**SP 6 (Sanyinjiao):** The meeting point of the Spleen, Liver, and Kidney channels. **Nourishes Blood and Yin**, promotes circulation in the lower abdomen and legs, and calms the spirit. *Extremely important for this condition.*

*	**LR 3 (Taichong):** The Source and Shu-Stream point of the Liver channel. **Soothes the Liver**, regulates Qi, alleviates stagnation, and helps to extinguish internal wind, which is responsible for the restless, involuntary movement.

*	**GB 34 (Yanglingquan):** The Influential point for Tendons. **Relaxes tendons and muscles** and benefits the lateral aspect of the lower limbs. Essential for any tendon-related discomfort.

\#### 2. Distal Points (To address the root cause):

*	**LI 4 (Hegu):** Great for regulating Qi and Blood throughout the body and for its analgesic (pain-relieving) effect. Often paired with LR 3.

*	**BL 17 (Geshu):** The Influential point for Blood. **Nourishes Blood** and invigorates Blood circulation. Crucial for treating Blood deficiency and stasis.

*	**BL 18 (Ganshu):** Back-Shu point for the Liver. **Nourishes Liver Blood** and soothes the Liver.

*	**BL 23 (Shenshu):** Back-Shu point for the Kidney. **Nourishes Kidney essence**, which is the foundation of Yin and Yang, and strengthens the bones and lower back.

\#### 3. Points to Calm the Spirit (Shen):

The worsening at night is also a disturbance of the spirit due to lack of nourishment.

*	**HT 7 (Shenmen):** The Source point of the Heart channel. **Calms the mind and spirit** (Shen), which is essential for the restlessness and sleep disruption.

*	**EX-HN3 (Yintang):** Located between the eyebrows. Excellent for calming the mind and relieving stress.

\#### 4. Ah Shi Points:

In addition to the core points, the practitioner should palpate for tender or knotted spots (**Ah Shi points**) along the affected muscle groups in the calves and thighs (often along the Bladder, Stomach, Spleen, and Gallbladder channels) and needle them.

\### Proposed Treatment Plan

*	**Frequency:** 2-3 times per week initially.

*	**Technique:** A combination of even supplementation and drainage technique. For tonifying points like ST 36, SP 6, and the Back-Shu points, a reinforcing method would be used. For moving points like LR 3 and GB 34, a reducing or even method would be appropriate.

*	**Stimulation:** Needles would typically be retained for 20-30 minutes.

*	**Course of Treatment:** A course of 8-12 treatments would be standard to assess efficacy, given the chronic nature (2 years) of the condition.

\### Rationale for Acupuncture in this Case

The patient's reluctance towards medication makes acupuncture an excellent first-line intervention. Modern research suggests acupuncture may help RLS by:

*	Regulating dopamine and other neurotransmitters in the central nervous system.

*	Improving peripheral blood circulation.

*	Modulating the nervous system to reduce sensory discomfort and motor restlessness.

*	Releasing endogenous opioids for pain relief.

This point prescription addresses the condition from both a TCM pattern-based root cause perspective and a modern symptomatic treatment approach.

**Case 2**

Of course. This is a complex and serious case presentation requiring a careful and strategic acupuncture approach. The diagnosis of Cervical Ossification of the Posterior Longitudinal Ligament (C-OPLL) with myelopathy (indicated by the "walking on cotton" sensation, positive Babinski's sign, and lower limb weakness) and radiculopathy (left arm pain/numbness) means the treatment must focus on both managing pain and addressing the neurological symptoms.

**Important Disclaimer:** This is for educational and informational purposes only. Acupuncture for this condition should be considered a supportive and palliative management strategy, not a cure for the underlying spinal compression. It must be performed by a highly qualified practitioner with experience in neurological conditions. All treatment should be delivered with extreme caution to avoid any risk of injury. The patient's preference to avoid surgery is respected, but the serious nature of the diagnosis must be acknowledged.

---

\### TCM Pattern Differentiation

Based on the symptoms:

*	**Neck/Shoulder Pain, Stiffness, Tenderness:** Indicates **Qi and Blood Stasis** in the neck channels (primarily Shaoyang-GB, Taiyang-SI/BL, and Yangming-LI/ST).

*	**Numbness and Pain in Left Upper Limb:** Numbness is often due to **Blood deficiency failing to nourish the limbs** or **Qi deficiency failing to propel Blood**. Pain indicates **stasis** or **channel obstruction**.

*	**Weakness in Both Lower Limbs, "Walking on Cotton":** This is a key sign of **Kidney Essence and Qi Deficiency**. The Kidney governs the bones and marrow (which includes the spinal cord), and deficiency leads to weakness and instability.

*	**Tendency to Fall, Weakness:** Further indicates **Qi deficiency** and **Spleen Qi deficiency** (as the Spleen governs the muscles).

*	**Age (79 years old):** Naturally suggests underlying **deficiency** of Qi, Blood, and Kidney Essence.

**Primary TCM Diagnosis:** Underlying **Kidney and Liver Deficiency** (weakening bones and tendons) with a secondary pathogenic factor of **Qi and Blood Stasis** causing channel obstruction.

\### Treatment Principle

The strategy must be twofold:

1. **Benefit the Brain and Marrow, Tonify the Kidneys and Strengthen the Bones** (to address the root deficiency and myelopathy).

2. **Invigorate Blood, Resolve Stasis, Unblock the Channels, and Alleviate Pain** (to address the branch presentation of pain and numbness).

\### Suggested Acupuncture Points

Treatment should be gentle, focusing on distal points and careful local needling to avoid any aggravation.

\#### 1. Core Local Points (For the Neck - Use with CAUTION):

*	***Extreme care must be taken with needling depth and technique around the cervical spine. Avoid deep perpendicular needling into the spine.***

*	**GB 20 (Fengchi):** Located below the occiput. **Expels Wind, benefits the head and brain, unblocks the channel.** Crucial for neck stiffness and headaches. Needle carefully towards the contralateral eye.

*	**GB 21 (Jianjing):** On the shoulder apex. **Relaxes the sinews, moves Qi and Blood in the neck/shoulder region.** *Contraindicated for deep needling; use oblique insertion. Can be needled on the unaffected side or bilaterally with caution.*

*	**SI 14 (Jianwaishu) / SI 15 (Jianzhongshu):** Points on the scapula that strongly affect the shoulder and neck.

*	**Ah Shi Points:** Identify and gently needle tender spots in the trapezius and paravertebral muscles. **Do not needle directly into the spine.**

\#### 2. Distal Points for Upper Limb Radiculopathy (Left side):

*	**LI 4 (Hegu):** Great for pain and numbness in the face and upper body. Analgesic point. *Contraindicated in pregnancy.*

*	**SJ 5 (Waiguan):** Connects to the Yang Linking Vessel, excellent for releasing the exterior and treating pain along the Shaoyang channel (side of the neck, arm, and shoulder).

*	**LI 11 (Quchi):** He-Sea point, regulates Qi and Blood in the entire arm, relieves pain and numbness.

*	**Ba Xie (EX-UE9):** For numbness and pain in the fingers.

\#### 3. Points for Lower Limb Weakness and Myelopathy:

*	**ST 36 (Zusanli):** He-Sea point of the Stomach channel, Master point for tonifying Qi and Blood. Strengthens the entire body and the lower limbs.

*	**GB 34 (Yanglingquan):** Influential point for Tendons. Strengthens tendons and benefits motor function.

*	**BL 60 (Kunlun):** Unblocks the Taiyang channel (which runs along the spine and back of the legs) and relieves stiffness.

*	**LV 3 (Taichong):** Source point of the Liver channel. The Liver governs the tendons. Used to soothe the body and regulate Qi.

\#### 4. Points to Benefit the Marrow and Tonify the Kidney (Root Treatment):

*	**GV 20 (Baihui):** Raises Yang Qi, benefits the brain, and clears the senses. Essential for treating the "walking on cotton" sensation and neurological deficits.

*	**GV 14 (Dazhui):** Meeting point of all Yang channels. Can help regulate Qi in the spine. **Must be needled with extreme caution.**

*	**BL 11 (Dazhu):** Influential point for Bone. Directly addresses bone pathology.

*	**BL 23 (Shenshu):** Back-Shu point of the Kidney. **Tonifies Kidney Essence** to strengthen the bones and marrow.

*	**KI 3 (Taixi):** Source point of the Kidney channel. Strongly tonifies Kidney Yin and Yang.

\### Proposed Treatment Plan & Adjunctive Therapies

*	**Frequency:** Begin with 2-3 treatments per week. Monitor progress closely.

*	**Technique:** Use a tonifying method for points like ST 36, BL 23, and KI 3. Use even method for the distal points on the arm. Use very gentle stimulation on local neck points.

*	**Electroacupuncture:** May be carefully applied to local Ah Shi points (e.g., on the shoulder) and distal points on the arm (e.g., LI 11 to SJ 5) using a low-frequency, low-intensity setting. **Avoid placing electrodes directly over the cervical spine.**

*	**Moxibustion:** **Highly recommended.** Moxa can be applied to points like ST 36, BL 23, and GV 14 (with caution) to warm the channels, move Qi and Blood, and tonify Yang Qi, which is crucial for an elderly patient with weakness.

*	**Tuina Massage:** Gentle, non-manipulative massage to the neck and shoulder muscles can help relax stiffness and improve circulation without risking further compression.

*	**Lifestyle Advice:** The patient must be counseled on neck care: using a supportive pillow, avoiding sudden movements, and avoiding activities that strain the neck (like looking down at a phone for long periods).

\### Rationale

This approach aims to:

1. **Reduce local muscle spasms** and improve blood flow to alleviate pain.

2. **Stimulate neurological function** through distal and scalp points.

3. **Address the underlying degenerative process** by tonifying the Kidneys.

4. **Improve overall strength and stability** by tonifying Qi.

While acupuncture cannot reverse the ossification, it can be a valuable tool for managing pain, improving quality of life, and potentially slowing the progression of functional decline by improving circulation and reducing inflammatory stasis. Close monitoring for any signs of neurological deterioration is essential.

**Case 3**

Of course. This is a complex case of Polymyalgia (PM), likely Polymyalgia Rheumatica, though the high Creatine Kinase (CK) level also suggests a possible overlap with Polymyositis. The patient's advanced age, chronicity of the condition, and presentation of weakness, soreness, and severe tearing pain require a sophisticated acupuncture approach focused on both the root deficiency and the branch excess.

**Important Disclaimer:** This is for educational and informational purposes only. Acupuncture is a complementary therapy and should not replace standard medical care for a condition as serious as PM. The decision to stop medication is significant and should be made in close consultation with a rheumatologist. This point prescription is based on Traditional Chinese Medicine (TCM) pattern differentiation.

---

\### TCM Pattern Differentiation

Based on the symptoms:

*	**Chronic Duration (10+ years), Advanced Age (66), Relapsing Condition:** All strongly point to an underlying **deficiency**, specifically of **Qi and Blood**, and likely **Spleen, Liver, and Kidney deficiency**. The Spleen is responsible for muscle strength, the Liver governs the tendons and stores Blood, and the Kidney governs the bones and marrow.

*	**Generalized Muscle Weakness:** Directly indicates **Spleen Qi Deficiency** (as the Spleen governs the muscles and four limbs).

*	**Muscle Soreness and Severe "Tearing" Limb Pain:** This describes **Bi Syndrome** (painful obstruction syndrome). The tearing quality is a classic sign of **Blood Stasis**. The aggravation after a cold (an external pathogen) suggests an element of **Wind, Cold, and Dampness** invading the channels and obstructing the flow of Qi and Blood.

*	**Relapse after a Cold:** A cold is an invasion of **External Wind**, which can stir up latent pathogenic factors and exacerbate an underlying deficient condition.

**TCM Diagnosis:** A combination of **Underlying Qi and Blood Deficiency** (root) with **Acute Exacerbation of Wind-Cold-Damp Obstruction with Blood Stasis** (branch). This is a classic deficiency-excess complex pattern.

\### Treatment Principle

The strategy must be dual:

1. **Tonify Qi and Blood, Strengthen the Spleen, Liver, and Kidney** (to address the root deficiency and the weakness).

2. **Expel Wind, Dispel Dampness, Warm the Channels, Invigorate Blood, and Relieve Pain** (to address the branch excess and the tearing pain).

\### Suggested Acupuncture Points

Treatment should be gentle but can be more stimulating on the limbs. Moxibustion is highly recommended.

\#### 1. Points to Tonify Qi and Blood (Root Treatment):

*	**ST 36 (Zusanli):** The He-Sea point of the Stomach channel. **Master point for tonifying Qi and Blood.** Strengthens the Spleen and Stomach to improve the generation of Qi and Blood, directly addressing weakness.

*	**SP 6 (Sanyinjiao):** The meeting point of the Spleen, Liver, and Kidney channels. **Nourishes Blood, tonifies Liver and Kidney Yin, and strengthens the Spleen.** A crucial point for this case.

*	**CV 6 (Qihai):** Sea of Qi. **Strongly tonifies and regulates Yuan (Source) Qi** throughout the body, improving energy levels and overall resistance.

*	**CV 4 (Guanyuan):** With moxibustion, this point **tonifies Kidney Yang and Yuan Qi**, and benefits essence. Important for an elderly patient.

*	**BL 20 (Pishu) & BL 23 (Shenshu):** Back-Shu points for the Spleen and Kidney. **Tonify the Spleen to benefit the muscles and tonify the Kidney to strengthen the bones.** Best used with moxa.

\#### 2. Points to Expel Pathogens and Relieve Pain (Branch Treatment):

*	**LI 4 (Hegu):** Command point of the face and mouth, great for pain. **Expels Wind, alleviates pain, and regulates Qi.** Often paired with LI 11.

*	**LI 11 (Quchi):** He-Sea point. **Clears Heat, expels Wind, and resolves Dampness.** Very effective for Bi Syndrome and arm pain.

*	**GB 34 (Yanglingquan):** Influential point for Tendons. **Relaxes tendons and muscles, alleviates pain,** and benefits the lateral side of the lower limbs.

*	**SP 9 (Yinlingquan):** He-Sea point of the Spleen channel. **Resolves and transforms Dampness,** which is a key pathogen in Bi Syndrome causing soreness and heaviness.

*	**ST 40 (Fenglong):** Luo-Connecting point. **Resolves Phlegm and Dampness,** and calms pain.

\#### 3. Points for Severe "Tearing" Pain (Blood Stasis):

*	**SP 10 (Xuehai):** Sea of Blood. **Strongly invigorates Blood and dispels Blood Stasis.** Primary point for any pain with a stabbing or tearing quality.

*	**BL 17 (Geshu):** Influential point for Blood. **Nourishes Blood and invigorates Blood circulation.**

*	**Ah Shi Points:** Identify and needle the most tender spots in the painful limb muscles. This is a "where it hurts, needle it" approach to directly move Qi and Blood in the affected area.

\#### 4. Points to Expel Wind (Due to relapse after cold):

*	**GB 20 (Fengchi):** Pool of Wind. **Expels Wind** (both internal and external) and benefits the head and neck. A key point for any condition involving Wind.

*	**GV 14 (Dazhui):** Meeting point of all Yang channels. **Expels Wind and releases the exterior,** especially useful if there's any lingering external pathogen. **Excellent with moxibustion.**

\### Proposed Treatment Plan & Adjunctive Therapies

*	**Frequency:** 2-3 times per week initially. This is a chronic condition requiring a long-term commitment.

*	**Technique:** Use a **tonifying method** on points like ST 36, SP 6, CV 6. Use an **even method** or mild reducing method on points for pain like LI 4, LI 11, GB 34, and SP 10.

*	**Moxibustion is CRITICAL:** Given the age and likely underlying Yang deficiency, moxa should be applied to points like **ST 36, SP 6, CV 4, CV 6, BL 20, BL 23, and GV 14.** Moxa warms the channels, expels Cold and Damp, moves Qi and Blood, and tonifies Yang Qi brilliantly.

*	**Electroacupuncture:** May be applied to local Ah Shi points on the limbs (e.g., on the thigh or calf muscles) to provide strong, consistent pain relief and stimulate blood flow.

*	**Cupping:** Can be used on the back along the Bladder channel (e.g., on BL 17, BL 20, BL 23) and on the limbs to help drain Dampness and move Stasis. Use a light-to-medium suction only.

\### Rationale

This comprehensive approach aims to:

1. **Boost the patient's fundamental energy** (Qi and Blood) to reduce weakness and prevent relapse.

2. **Attack the pathogenic factors** (Wind, Damp, Stasis) causing the acute, severe pain.

3. **Improve local circulation** in the muscles to alleviate soreness and promote healing.

By addressing both the root and the branch, acupuncture can potentially help manage pain, reduce inflammation, improve muscle function, and enhance the quality of life for this patient, allowing for a possible reduction in her reliance on medication. However, close monitoring of her CK levels and symptoms by a physician is still strongly advised.

**Case 4**

Of course. This is a highly complex neurological case, as evidenced by the MRI and MRA findings pointing to a brainstem stroke (likely ischemic) affecting the pons, thalami, and corticospinal tracts. The presentation includes a mix of motor weakness, sensory ataxia, and bulbar symptoms (dysphagia, dysarthria).

**Important Disclaimer:** This is for educational and informational purposes only. Acupuncture in this context must be considered a **complementary therapy** within a comprehensive multidisciplinary rehabilitation program led by neurologists and physical, occupational, and speech therapists. It is not a substitute for standard medical care. Treatment must be administered by a practitioner highly experienced in post-stroke neurological rehabilitation.

---

\### TCM Pattern Differentiation

Based on the symptoms and location:

*	**Limb Weakness, Gait Instability, Ataxia:** This indicates **Qi Deficiency** and **Spleen Qi Deficiency** (as the Spleen governs the muscles) failing to control the limbs. The ataxia and disordered proprioception specifically point to a profound **Kidney Essence Deficiency**, as the Kidney governs the marrow (which includes the brain) and is the root of balance and stability.

*	**Concealed Aspiration, Weakened Gag Reflex, Dysarthria:** These are bulbar symptoms. In TCM, the tongue is the orifice of the Heart, and speech is governed by the Heart Spirit. Swallowing involves the ascending of Spleen Qi and descending of Stomach Qi. This complex points to **Phlegm misting the orifices** and **Qi deficiency affecting the Heart and Spleen**.

*	**Bilateral Involvement, Corticospinal Tract Signs:** The bilateral weakness and hyperreflexia suggest a central pattern of **Liver Wind**, which is the primary TCM mechanism for stroke. However, in the recovery phase, the Wind has often settled, leaving behind **Qi Deficiency, Blood Stasis, and Phlegm Obstruction**.

*	**Location (Pons, Thalami, Brainstem):** This area is the core of vital functions. Its impairment suggests a deep disturbance of the **Du Mai (Governing Vessel)**, which governs the brain and spinal cord, and the **Yin Qiao and Yang Qiao Mai** (Heel Vessels), which regulate motor function and muscle tone.

**Primary TCM Diagnosis:** **Qi and Kidney Essence Deficiency with Phlegm and Blood Stasis obstructing the Du Mai and orifices.**

\### Treatment Principle

The strategy must be multi-targeted:

1. **Awaken the Brain and Open the Orifices** (to address the core neurological deficit).

2. **Benefit the Du Mai and Marrow** (to strengthen the brain and spinal cord).

3. **Tonify Qi and Strengthen the Spleen** (to improve muscle strength).

4. **Nourish the Liver and Kidneys** (to improve balance and proprioception).

5. **Resolve Phlegm and Promote Swallowing** (to address the bulbar symptoms).

6. **Invigorate Blood and Unblock the Channels** (to improve limb function).

\### Suggested Acupuncture Points

Treatment should be gentle but can incorporate stronger stimulation on the limbs. Scalp acupuncture is **extremely important** in this case.

\#### 1. Scalp Acupuncture (Crucial for Neurological Recovery):

*	**Motor Area (Middle 2/5):** Corresponds to the upper and lower limbs. Essential for treating motor weakness and improving voluntary movement. **Stimulation on the contralateral side.**

*	**Sensorimotor Area (Overlap of Motor and Sensory Areas):** Ideal for addressing both weakness and proprioceptive disorder.

*	**Balance Area (Cerebellar Brainstem Area):** Located below the external occipital protuberance. **Primary area for treating ataxia, dizziness, and balance disorders.**

*	**Second Speech Area (Temporal 3 Needle):** Located over the temporal region, superior to the pinna. Used for motor aphasia and dysarthria.

*	**Chorea and Tremor Area (Frontal 4 Needle):** Can help regulate muscle tone and coordination.

\#### 2. Body Points for Neurological and Motor Function:

*	**GV 20 (Baihui):** Raises Yang Qi, benefits the brain, and clears the senses. A master point for all neurological and mental functions.

*	**GV 26 (Shuigou / Renzhong):** Strongly awakens the brain and opens the orifices. Used for loss of consciousness and neurological shock. Critical for stimulating neurological recovery.

*	**GB 20 (Fengchi):** Expels Wind, benefits the brain and brainstem, and clears the orifices. A key point for any brainstem-related issue.

*	**HT 5 (Tongli):** Luo-Connecting point of the Heart channel. Benefits the tongue and speech. Specific for treating dysarthria and aphasia.

*	**ST 36 (Zusanli) + SP 6 (Sanyinjiao):** This combination powerfully **tonifies Qi and Blood** and strengthens the Spleen to benefit the muscles. SP 6 also nourishes the Liver and Kidneys.

*	**LI 4 (Hegu) + LI 11 (Quchi):** Command points for the face and arm. Regulate Qi and Blood in the upper limb, alleviate pain and weakness, and expel wind.

*	**GB 34 (Yanglingquan):** Influential point for Tendons. Essential for improving motor function and strength in the lower limbs.

*	**KI 3 (Taixi):** Source point of the Kidney. **Tonifies Kidney Essence** to strengthen the bones, benefit the brain, and improve balance.

\#### 3. Points for Swallowing (Dysphagia):

*	**CV 23 (Lianquan):** Located on the neck. Directly benefits the tongue and promotes swallowing.

*	**Jia Chengjiang (EX-HN17 - Located 1 cun lateral to CV 24):** Local points to stimulate the pharyngeal muscles.

*	**PC 6 (Neiguan):** Opens the chest, regulates Qi, and calms the Spirit. Often used for nausea and to regulate the descending function of Stomach Qi, which aids swallowing.

\#### 4. Points for Proprioception and Ataxia:

*	**SI 3 (Houxi) + BL 62 (Shenmai):** This pair opens and regulates the **Du Mai and Yang Qiao Mai** respectively. These extraordinary vessels are paramount for coordinating movement, balance, and posture.

*	**KI 6 (Zhaohai):** Opens the **Yin Qiao Mai**, which works with Yang Qiao to regulate motor function and muscle tone.

\### Proposed Treatment Plan

*	**Frequency:** Daily or every other day treatment is ideal, especially in the early recovery phase.

*	**Technique:** Scalp needles should be retained for 30-60 minutes and stimulated manually or with electroacupuncture (low frequency, e.g., 2-10Hz) every 10-15 minutes.

*	**Electroacupuncture:** Can be very effective on body points for limb rehabilitation (e.g., LI 11 to LI 10 for arm extension, ST 36 to GB 34 for leg movement). Use low frequency.

*	**Rehabilitation Synergy:** The acupuncture treatment should be immediately followed by physical therapy, occupational therapy, and speech therapy. The neuromodulatory effects of acupuncture can create a "window of plasticity" where the brain is more receptive to retraining.

\### Rationale

This comprehensive prescription is designed to:

1. **Directly stimulate brain repair** through scalp acupuncture targeting motor, sensory, balance, and speech centers.

2. **Address the root deficiency** (Kidney and Qi) causing the weakness and instability.

3. **Target the specific symptoms** of dysphagia and dysarthria with local and distal points.

4. **Regulate the extraordinary vessels** responsible for complex motor coordination and balance.

The goal is to support the patient's extensive rehabilitation efforts, improve neurological function, enhance quality of life, and maximize recovery potential.

**Case 5**

Of course. This is a classic case of homonymous hemianopia resulting from a posterior circulation (vertebrobasilar) stroke, likely affecting the occipital lobe (visual cortex) or the optic radiations.

**Important Disclaimer:** This is for educational and informational purposes only. Acupuncture is a complementary therapy and should be integrated into a comprehensive rehabilitation plan. Treatment must be administered by a qualified practitioner. While there is no pharmaceutical "cure," acupuncture has shown promise in neuroplasticity and visual field rehabilitation.

---

\### TCM Pattern Differentiation

Based on the symptoms and location:

*	**Sudden Onset Limb Weakness & Vision Loss:** The sudden onset is characteristic of **Internal Wind**, the primary TCM mechanism of stroke.

*	**Blurred Vision, Reduced Visual Fields (Hemianopia):** The eyes are governed by the Liver (**"Liver opens into the eyes"**). Vision is also related to the essence (**Kidney**) and the blood that nourishes the eyes (**Heart and Liver Blood**). A stroke affecting vision suggests:

*	**Liver Blood and Kidney Yin Deficiency:** Failing to nourish the eyes.

*	**Qi and Blood Stasis:** Obstructing the channels that connect to and supply the eyes, particularly the channels reaching the occiput.

*	**Posterior Circulation Infarction:** This area relates to the **Taiyang (Bladder) Channel** which traverses the occiput and entire back, and the **ShaoYang (Gallbladder) Channel** which traverses the sides of the head. Obstruction in these channels can directly affect vision.

**Primary TCM Diagnosis:** **Liver and Kidney Yin Deficiency with Blood Stasis obstructing the channels and orifices of the eyes.**

\### Treatment Principle

The strategy is to:

1. **Nourish the Liver and Kidneys** to address the root deficiency.

2. **Invigorate Blood and Resolve Stasis** to clear the obstruction.

3. **Brighten the Eyes** and specifically target the visual pathways.

4. **Regulate the Qi and Blood in the affected channels** (especially Taiyang and Shaoyang).

\### Suggested Acupuncture Points

This prescription combines local peri-ocular points, distal points on channels affecting vision, and points to promote general neurological recovery.

\#### 1. Local Points Around the Eyes (To regulate Qi and Blood locally):

*	**BL 1 (Jingming):** Where the Bladder, Small Intestine, and Stomach channels meet. **The primary point for all eye disorders.** Needled carefully along the orbit.

*	**GB 1 (Tongziliao):** On the lateral canthus. Benefits the eye and clears Shaoyang channel heat.

*	**EX-HN5 (Taiyang):** Extra point at the temple. Important for vision, headaches, and lateral eye issues.

*	**Yu Yao (EX-HN4):** In the middle of the eyebrow. Benefits the eye.

*	**Sizhukong (SJ 23):** At the lateral end of the eyebrow. Benefits the eye and lateral canthus.

\#### 2. Distal Points on Limb Channels (To nourish the root and move Qi/Blood):

*	**LR 3 (Taichong):** **Source point of the Liver channel.** Crucial for nourishing Liver Blood and subduing Liver Wind. A must-use point.

*	**KI 3 (Taixi):** **Source point of the Kidney channel.** Tonifies Kidney Yin and Essence to nourish the eyes.

*	**GB 37 (Guangming):** **Luo-Connecting point of the Gallbladder channel.** Its name means "Bright Light." It is the **key distal point specifically for treating eye disorders and visual blurriness**, especially those involving the Gallbladder channel.

*	**GB 20 (Fengchi):** Expels Wind, benefits the eyes and ears, and clears the occiput. Directly affects the posterior circulation area.

*	**BL 67 (Zhiyin):** The Jing-Well point of the Bladder channel. **Strongly activates the Taiyang channel and benefits the eyes.** Often used with moxa.

\#### 3. Scalp Acupuncture (CRITICAL for Neurological Retraining):

*	**Visual Area (Optic Vision):** This is located on the scalp, horizontally 1 cun lateral and 4 cun superior to the external occipital protuberance (Du 17 / GV 17), then extending 1 cun anteriorly and posteriorly from that point. **This is the single most important area to needle for hemianopia.** Needles are inserted and stimulated (manually or with electroacupuncture) for 30-60 minutes. The stimulation is applied on the side **contralateral to the visual field defect**.

\#### 4. Points for General Post-Stroke Recovery:

*	**GV 20 (Baihui):** Raises Yang Qi and benefits the brain. Essential for all post-stroke neurological conditions.

*	**LI 4 (Hegu) + LI 11 (Quchi):** Regulate Qi and Blood in the entire body and upper limbs, helping with the residual limb weakness.

*	**ST 36 (Zusanli) + SP 6 (Sanyinjiao):** Tonify Qi and Blood. SP 6 also nourishes the Liver, Spleen, and Kidney.

\### Proposed Treatment Plan

*	**Frequency:** Treatment should be frequent, ideally **2-3 times per week** initially. Visual field retraining requires consistent stimulation.

*	**Technique:**

*	Local eye points (BL 1, GB 1) should be needled gently with a thin needle.

*	The **Scalp Visual Area** must be needled and stimulated vigorously (by twirling or with low-frequency electroacupuncture) for at least 5-10 minutes every 15 minutes during the retention period.

*	Body points can be stimulated with even method.

*	**Retention Time:** 30-45 minutes.

*	**Adjunctive Therapy:** The patient should be encouraged to perform **visual field training exercises** (e.g., tracking objects from the non-seeing field into the seeing field) **DURING** the acupuncture session while the scalp needles are in place. This leverages the concept of "cueing" the brain during a state of heightened neuroplasticity induced by acupuncture.

\### Rationale

This approach is designed to:

1. **Stimulate the visual cortex directly** via scalp acupuncture to encourage rewiring and reactivation of damaged pathways (neuroplasticity).

2. **Increase blood flow and energy to the eyes** through local points.

3. **Address the underlying TCM pattern** (Liver/Kidney deficiency and Blood stasis) that created the vulnerability for the stroke and is hindering recovery.

4. **Integrate with modern rehabilitation principles** by combining needling with active visual field training.

The goal is to expand the functional visual field, improve quality of life, and support the patient's overall neurological recovery.

**Case 6**

Of course. This is a classic presentation of Temporomandibular Joint Disorder (TMD) of myofascial origin. The acupuncture approach will focus on relaxing the affected muscles, relieving pain, reducing inflammation, and restoring normal function to the jaw.

**Important Disclaimer:** This is for educational and informational purposes only. Acupuncture is a medical practice that must be performed by a qualified and licensed practitioner. A thorough examination is necessary to rule out other causes of TMJ pain (e.g., dental issues, joint degeneration). This prescription is based on the standard TCM approach for such a presentation.

---

\### TCM Pattern Differentiation

Based on the symptoms:

*	**Pain in the Temporomandibular Area:** This area is traversed by the **Yangming Stomach Channel** (ST), **Shaoyang Gallbladder Channel** (GB), and the **Taiyang Bladder Channel** (BL).

*	**Difficulty Opening Mouth, Pain with Eating/Talking:** Indicates **Qi and Blood Stasis** (obstruction) in the local channels, leading to stiffness and pain.

*	**Tender Temporalis, Masseter, Trapezius, and Splenius Capitis Muscles:** Confirms the diagnosis of muscular tension and myofascial pain syndrome. The involvement of the neck and shoulder muscles (trapezius, splenius) suggests the pattern has spread along the channel pathways, particularly the **Shaoyang (GB) and Taiyang (BL) channels**.

*	**Gradual Increase in Pain:** Suggests a chronic condition, often related to factors like stress (causing Qi stagnation), chronic overuse, or poor posture.

**Primary TCM Diagnosis:** **Qi and Blood Stasis in the Yangming and Shaoyang Channels.**

\### Treatment Principle

The strategy is to:

1. **Regulate Qi and Blood in the local channels.**

2. **Relax the tendons and muscles** (舒缓筋脉).

3. **Soothe the Liver** (疏肝) – as the Liver governs the tendons and emotional stress is a major contributor to TMD.

4. **Alleviate pain.**

\### Suggested Acupuncture Points

This prescription will include local Ah Shi points, distal points on the affected channels, and points to address systemic tension.

\#### 1. Local and Adjacent Points (To regulate Qi and Blood in the area):

*	**ST 7 (Xiaguan):** **The key local point for TMJ disorders.** Located in the depression between the zygomatic arch and the mandibular notch. Directly affects the TMJ, masseter muscle, and jaw opening.

*	**ST 6 (Jiache):** Located at the prominence of the masseter muscle when the teeth are clenched. Excellent for relaxing the masseter muscle and treating jaw pain.

*	**SI 19 (Tinggong):** Located anterior to the tragus of the ear, in a depression that is revealed when the mouth is open. A very important adjacent point for TMJ pain and dysfunction.

*	**GB 2 (Tinghui):** Located anterior to the intertragic notch, posterior to the condyle of the mandible. Another crucial adjacent point on the Shaoyang channel.

*	**GB 20 (Fengchi):** Located below the occiput. Expels Wind, relieves pain, and is exceptionally effective for releasing tension in the trapezius and splenius capitis muscles.

*	**Ah Shi Points:** Identify and needle the most tender spots in the **temporalis, masseter, trapezius, and splenius capitis muscles.** This is a "where it hurts, needle it" approach.

\#### 2. Distal Points (To treat the root and regulate the channels from afar):

*	**LI 4 (Hegu):** Command point of the face and mouth. **Powerful analgesic point for any pain in the head and face.** Strongly regulates Qi and Blood. *Contraindicated in pregnancy.*

*	**LV 3 (Taichong):** Source point of the Liver channel. **Soothes the Liver, relieves Qi stagnation** (often caused by stress), relaxes tendons, and alleviates pain. The combination of LI 4 and LV 3 is called the **"Four Gates"** and is superb for regulating Qi and Blood throughout the body and relieving pain.

*	**SI 3 (Houxi):** Connects to the Du Mai (Governing Vessel). Excellent for releasing tension along the entire Taiyang channel, which includes the neck and scapula (trapezius).

*	**GB 34 (Yanglingquan):** Influential point for Tendons. **Relaxes tendons and muscles** throughout the body, making it essential for any musculoskeletal disorder, including TMD.

\#### 3. Points to Address Systemic Stress (A common underlying factor):

*	**Yintang (EX-HN3):** Located between the eyebrows. Calms the mind (Shen) and reduces stress.

*	**HT 7 (Shenmen):** Source point of the Heart channel. Calms the spirit and is very effective for anxiety and stress-related tension.

\### Proposed Treatment Plan

*	**Frequency:** 1-2 times per week initially.

*	**Technique:**

*	Local points (ST 6, ST 7, Ah Shi) can be needled with a mild reducing technique to release muscle spasm.

*	Distal points (LI 4, LV 3, GB 34) can be needled with a stronger stimulation to achieve De Qi sensation and guide it upward.

*	**Electroacupuncture:** Highly recommended. Electroacupuncture can be applied to local points (e.g., one needle in ST 6 and one in ST 7) using a low-frequency, dense-disperse setting to provide strong muscle relaxation.

*	**Cupping or Gua Sha:** Can be applied to the trapezius and neck muscles to release tension and improve blood flow.

*	**Patient Education:** The practitioner should advise the patient on:

*	Soft diet to rest the jaw.

*	Applying warm compresses to the tense muscles.

*	Avoiding chewing gum, nail-biting, and clenching the jaw.

*	Stress management techniques.

\### Rationale

This point prescription works by:

1. **Releasing local muscle spasms** in the jaw and neck through local and adjacent points.

2. **Stimulating the body's endogenous pain-relief systems** through powerful distal points like LI 4.

3. **Addressing the emotional component** (stress, tension) that often underlies and exacerbates TMD.

4. **Improving overall circulation of Qi and Blood** to reduce stasis and promote healing.

This multi-faceted approach is very effective for managing the pain and dysfunction associated with TMD.

**Case 7**

Of course. This is a case of Androgenetic Alopecia (AGA) that has not responded to pharmaceutical treatment (finasteride). The Traditional Chinese Medicine (TCM) approach is holistic, aiming to address the underlying patterns of imbalance that are causing the hair loss, rather than just the symptom.

**Important Disclaimer:** This is for educational and informational purposes only. Acupuncture is a medical practice that must be performed by a qualified and licensed practitioner. Treatment should be part of a comprehensive plan that may include dietary and lifestyle advice.

---

\### TCM Pattern Differentiation

Based on the symptoms:

*	**Hair Loss for >3 years, Fine Hair:** In TCM, hair is considered an extension of the Blood ("Hair is the surplus of Blood"). The condition and growth of hair depend on the nourishment from Blood and the energy from Qi. Chronic hair loss indicates a **deficiency of Qi and Blood**.

*	**Greasy Scalp, Itchy Scalp:** A greasy scalp indicates **Dampness** or **Damp-Heat** ascending to the head. Itchiness can be related to Wind or Heat.

*	**Fatigue:** A clear sign of **Qi Deficiency**.

*	**Lack of Response to Finasteride:** Suggests the root cause is not solely hormonal in a way addressable by that drug, but rather a deeper systemic imbalance from a TCM perspective.

A very common TCM pattern for this type of hair loss is a combination of:

1. **Blood Deficiency:** Failing to nourish the hair follicles.

2. **Kidney Jing Deficiency:** The Kidneys are believed to "manifest in the hair" and control reproduction and aging. AGA is often linked to a decline in Kidney essence.

3. **Liver Blood Deficiency:** The Liver stores Blood, which nourishes the tendons, nails, and hair. Stress (a common modern issue) can deplete Liver Blood and cause Qi stagnation.

4. **Spleen Qi Deficiency:** The Spleen is the source of Qi and Blood production. A weak Spleen fails to send adequate nourishment upward to the scalp and also leads to Dampness accumulation (greasiness).

**TCM Diagnosis:** **Kidney Jing Deficiency with Spleen Qi Deficiency leading to Blood Deficiency and Damp-Heat ascending to the scalp.**

\### Treatment Principle

The strategy must be dual:

1. **Tonify the Kidneys and Supplement Jing** (to address the root of growth and aging).

2. **Strengthen the Spleen and Tonify Qi** (to generate more Blood and resolve Dampness).

3. **Nourish Blood and Promote Circulation to the Scalp** (to directly nourish the hair follicles).

4. **Clear Heat and Resolve Dampness from the Scalp** (to address the greasiness and itchiness).

\### Suggested Acupuncture Points

\#### 1. Local Points (To stimulate blood flow to the scalp):

*	**GV 20 (Baihui):** Located at the vertex of the head. A major point for raising Yang Qi and lifting Qi to the head. It directly stimulates the scalp.

*	**DU 24 (Shenting) & GV 21 (Qianding):** Points on the Governing Vessel on the anterior hairline. Used to calm the spirit and benefit the head.

*	**MS 1 (Sishencong):** The "Four Spirit" points surrounding GV 20. Excellent for calming the mind and stimulating circulation to the scalp.

*	**Ah Shi Points:** Needling around the area of most significant hair loss or thinning.

\#### 2. Distal Points (To treat the root patterns):

*	**ST 36 (Zusanli):** The He-Sea point of the Stomach channel. **Master point for tonifying Qi and Blood.** Strengthens the Spleen and Stomach to improve the production of Qi and Blood.

*	**SP 6 (Sanyinjiao):** The meeting point of the Spleen, Liver, and Kidney channels. **Extremely important for this case.** It nourishes Blood, tonifies the Spleen, and supplements the Liver and Kidneys.

*	**LV 3 (Taichong):** Source point of the Liver channel. **Soothes the Liver, nourishes Liver Blood,** and regulates Qi. The combination of LV 3 and LI 4 (the Four Gates) can help regulate overall Qi and Blood flow.

*	**KI 3 (Taixi):** Source point of the Kidney channel. **Tonifies Kidney Yin and Yang** and strengthens the Kidney essence, which is fundamental for hair health.

*	**LI 11 (Quchi):** He-Sea point. Clears Heat and Dampness from the body, helping to address the greasy and itchy scalp.

*	**SP 9 (Yinlingquan):** He-Sea point of the Spleen channel. **Resolves Dampness** from the body, targeting the greasy scalp.

\### Proposed Treatment Plan & Adjunctive Therapies

*	**Frequency:** Treatment requires consistency. 1-2 times per week is standard.

*	**Technique:** Use a **tonifying method** on points like ST 36, SP 6, KI 3. Use an **even method** on local scalp points and points like LI 11 to clear damp-heat.

*	**Moxibustion:** **Highly recommended.** Moxa can be applied to ST 36 and SP 6 to strongly tonify Qi and Blood. It is warming and helps to resolve Dampness.

*	**Plum Blossom (7-Star) Needle:** This is a **critical technique** for alopecia. The scalp is gently tapped with the plum blossom hammer until slight erythema (redness) appears. This stimulates local blood circulation and wakes up the hair follicles. It is often done before needling the local points.

*	**Cupping:** Can be used on the back along the Bladder channel (e.g., on BL 17, BL 18, BL 20, BL 23) to further tonify Blood, Spleen, and Kidneys.

*	**Lifestyle and Dietary Advice:** This is paramount. The practitioner should advise:

*	**Diet:** Eat nourishing, Blood-building foods (e.g., dark leafy greens, beets, dates, lean red meat, bone broth). Avoid greasy, sugary, and dairy-heavy foods that contribute to Dampness.

*	**Stress Management:** Encourage practices like Qi Gong, meditation, or yoga, as stress depletes Liver Blood.

*	**Sleep:** Ensure adequate rest before 11 PM, as this is the time the Liver Blood is replenished.

\### Rationale

This approach moves beyond just stimulating the scalp. It aims to:

1. **Improve the quality and quantity of Blood** (the material that hair is made from).

2. **Strengthen the digestive system** (Spleen) to ensure continuous production of high-quality Blood.

3. **Fortify the root of growth and aging** (Kidneys).

4. **Clear the pathogenic factors** (Damp-Heat) that are clogging the follicles and preventing healthy growth.

5. **Directly stimulate the follicles** through local needling and the plum blossom technique.

Given the chronic nature of the condition (3+ years), the patient should be advised that TCM treatment requires patience and consistency, often over several months, to see noticeable changes.

**Case 8**

Of course. This is another case of Androgenetic Alopecia (AGA) with a slightly different presentation from the previous one. The TCM approach will be tailored to the specific symptoms described.

**Important Disclaimer:** This is for educational and informational purposes only. Acupuncture must be performed by a qualified and licensed practitioner. This suggestion is based on TCM pattern differentiation.

---

\### TCM Pattern Differentiation

Based on the symptoms:

*	**Hair Loss for >2 years, worsening progressively:** This indicates a chronic condition with an underlying **deficiency**. The progressive nature suggests the root cause is not being addressed.

*	**Itchy Scalp:** This suggests the presence of a pathogenic factor, most commonly **Wind** or **Heat**,

at the surface level.

*	**Fatigue:** A clear sign of **Qi Deficiency**.

*	**Anorexia (loss of appetite):** This is a crucial symptom. It points directly to **Spleen Qi Deficiency**. The Spleen is responsible for transforming food and drink into Qi and Blood. Anorexia means this function is impaired, leading to inadequate production of Qi and Blood.

This combination of symptoms paints a clearer picture than the previous case:

**Primary TCM Diagnosis: Spleen Qi Deficiency with Failure to Produce Blood, leading to Blood Deficiency that fails to nourish the hair. This is combined with Wind-Heat or Damp-Heat disturbing the scalp.**

The anorexia is the key. The engine (Spleen) that makes the fuel (Qi and Blood) for the body (and hair) is weak.

\### Treatment Principle

The strategy will focus on:

1. **Strengthen the Spleen and Stomach** (to resolve anorexia and generate Qi and Blood).

2. **Tonify Qi and Nourish Blood** (to address the root deficiency causing the hair loss).

3. **Expel Wind and Clear Heat** from the scalp (to relieve the itching).

4. **Guide Qi and Blood to the head** to nourish the hair follicles.

\### Suggested Acupuncture Points

\#### 1. Core Points to Tonify Qi and Blood (Root Treatment):

*	**ST 36 (Zusanli):** **He-Sea point of the Stomach channel.** The primary point for tonifying Qi, strengthening the Spleen and Stomach, and treating fatigue. **Essential for this case due to anorexia.**

*	**SP 6 (Sanyinjiao):** Meeting point of the Spleen, Liver, and Kidney channels. **Nourishes Blood, strengthens the Spleen,** and aids digestion.

*	**CV 12 (Zhongwan):** **Front-Mu point of the Stomach.** Master point for treating all Stomach disorders and for strengthening digestion and resolving anorexia.

*	**BL 20 (Pishu) & BL 21 (Weishu):** Back-Shu points for the Spleen and Stomach. **Tonify Spleen Qi** and improve digestive function. Best used with moxibustion.

\#### 2. Points for the Scalp and Hair (Branch Treatment):

*	**GV 20 (Baihui):** Raises Yang Qi to the head. Stimulates the scalp.

*	**EX-HN1 (Sishencong):** The four points surrounding GV 20. Calm the spirit and stimulate local circulation.

*	**Ah Shi Points:** Needle around the thinning areas on the vertex.

*	**LI 11 (Quchi):** He-Sea point. **Clears Heat and Wind from the body,** helping to reduce scalp itching.

\#### 3. Points to Regulate Qi and Support Digestion:

*	**PC 6 (Neiguan):** Luo-Connecting point of the Pericardium channel. **Opens the chest, regulates Qi,**

and harmonizes the Stomach. Very useful for improving appetite and reducing stress that can affect digestion.

*	**LV 13 (Zhangmen):** Front-Mu point of the Spleen. Benefits the Spleen and alleviates abdominal distension.

\### Proposed Adjunctive Therapies

*	**Moxibustion is highly recommended.** Applying moxa to **ST 36, CV 12, and BL 20** will provide strong warming and tonifying effects to the digestive system, which is crucial for this patient.

*	**Plum Blossom (7-Star) Needling:** Tapping the scalp with a plum blossom hammer to cause mild redness is critical to stimulate blood flow to the follicles.

*	**Dietary Therapy is Paramount:** The practitioner must provide guidance:

*	Eat warm, cooked, easily digestible foods (e.g., soups, stews, congee).

*	Avoid cold, raw, and greasy foods, which weaken the Spleen.

*	Incorporate Spleen-tonifying foods like sweet potatoes, pumpkin, carrots, ginger, and lean proteins.

\### Comparison to the Previous AGA Case (38-year-old male)

| Feature | 38-Year-Old Patient | 33-Year-Old Patient (This Case) |

| :--- | :--- | :--- |

| **Key Symptom** | Greasy, flat hair | **Anorexia** (Loss of appetite) |

| **Primary TCM Pattern**| Kidney Jing Deficiency + Damp-Heat | **Spleen Qi Deficiency** (leading to Blood Deficiency) |

| **Pathogenic Factor** | Significant **Dampness/Heat** | **Wind-Heat** (itching) |

| **Treatment Focus** | Tonify Kidneys, resolve Damp-Heat | **Tonify Spleen/Stomach,** generate Qi/Blood |

| **Key Points** | KI 3, SP 9, LI 11 | **CV 12, ST 36, BL 20** |

\### Rationale

This treatment plan directly addresses this patient's core issue: a weak digestive system (Spleen Qi Deficiency) that is unable to produce the Qi and Blood necessary to grow and sustain hair. By focusing on strengthening digestion first, the body's fundamental energy is bolstered. This, combined with local scalp stimulation and clearing minor Wind-Heat, creates a comprehensive strategy to halt hair loss and encourage regrowth. The emphasis must be on dietary changes and consistent treatment to strengthen the Spleen.

**Case 9**

Of course. This is a detailed case of Patellofemoral Arthralgia (PFA) with clear MRI findings indicating degenerative changes, inflammation, and bone stress. The acupuncture approach will focus on pain relief, reducing inflammation, improving joint function, and addressing the underlying degenerative process.

**Important Disclaimer:** This is for educational and informational purposes only. Acupuncture is a complementary therapy and should be integrated into a comprehensive management plan that may include physiotherapy and lifestyle modification. Treatment must be administered by a qualified practitioner.

---

\### TCM Pattern Differentiation

Based on the symptoms and MRI findings:

*	**Chronic Knee Pain (1 year), worsened by squatting/stairs:** This is classic **Bi Syndrome** (painful obstruction syndrome). The specific aggravating activities point to **Qi and Blood Stasis** in the knee joint.

*	**Cartilage Damage, Degeneration, Bone Marrow Edema:** In TCM, this relates to **Kidney Deficiency**. The Kidney governs the bones and marrow. Deficiency leads to failure to nourish the bones and cartilage, making them susceptible to damage.

*	**Synovial Thickening, Joint Effusion, Swelling:** This indicates the presence of **Dampness** and **Phlegm** accumulation in the joint. The effusion and swelling are a form of pathological fluid.

*	**Recurrent Nature, Incomplete response to NSAIDs:** Suggests an underlying deficiency (Kidney) that is not addressed by anti-inflammatories alone.

**Primary TCM Diagnosis:** **Kidney and Liver Deficiency** (underlying root) with **Qi and Blood Stasis and Damp-Phlegm accumulation** in the knee joint (branch manifestations).

\### Treatment Principle

The strategy is threefold:

1. **Tonify the Kidneys and Strengthen the Bones** (to address the root degeneration).

2. **Resolve Dampness and Expel Phlegm** (to reduce swelling and effusion).

3. **Invigorate Blood, Remove Stasis, and Unblock the Channel** (to alleviate pain and restore mobility).

\### Suggested Acupuncture Points

\#### 1. Local and Peri-Articular Points (To regulate Qi and Blood in the knee):

*	**EX-LE4 (Neixiyan):** The medial "eye of the knee." A crucial point for any knee disorder, especially for effusion, swelling, and pain.

*	**EX-LE5 (Xiyan):** The lateral "eye of the knee." Directly targets the lateral knee pain described. Often needled together with Neixiyan.

*	**ST 34 (Liangqiu):** Xi-Cleft point of the Stomach channel. Located superior to the patella. **Excellent for acute pain and spasms** of the knee. Particularly good for patellofemoral pain.

*	**SP 10 (Xuehai):** Sea of Blood. Invigorates Blood circulation in the lower limb, crucial for treating chronic pain and stasis.

*	**SP 9 (Yinlingquan):** He-Sea point of the Spleen channel. **Resolves Dampness and reduces swelling** in the lower limb. Very important given the joint effusion.

*	**GB 34 (Yanglingquan):** Influential point for Tendons. Relaxes tendons and muscles and benefits the lateral aspect of the knee.

*	**Ah Shi Points:** Identify and needle any tender spots around the patella, patellar tendon, and lateral joint line.

\#### 2. Distal Points (To treat the root and regulate channels from afar):

*	**ST 36 (Zusanli):** He-Sea point of the Stomach channel. **Tonifies Qi and Blood** overall, strengthens the body, and benefits the knee joint.

*	**KI 3 (Taixi):** Source point of the Kidney channel. **Tonifies Kidney Essence** to strengthen the bones and marrow, addressing the degeneration.

*	**LV 8 (Ququan):** He-Sea point of the Liver channel. The Liver governs the tendons. This point nourishes Liver Blood to moisten tendons and ligaments around the joint.

*	**LI 4 (Hegu):** Command point for the face and mouth, but also a powerful analgesic point for pain anywhere in the body. Regulates Qi and Blood.

\#### 3. Points for Swelling and Effusion (To resolve Dampness):

*	**SP 9 (Yinlingquan)** and **ST 40 (Fenglong)** are the primary points to transform Phlegm and resolve Dampness systemically.

\### Proposed Treatment Plan & Adjunctive Therapies

*	**Frequency:** 1-2 times per week.

*	**Technique:**

*	Use a **tonifying method** on points like ST 36, KI 3.

*	Use an **even or reducing method** on local points like Xiyan, SP 10, and SP 9 to move stagnation and drain dampness.

*	**Moxibustion is HIGHLY recommended:** Given the chronic nature and likely underlying Yang deficiency (especially with edema), moxa is extremely beneficial.

*	**Direct Moxa:** Can be applied to **EX-LE5 (Xiyan)** and **ST 36** to warm the channel, expel cold-damp, and dramatically reduce pain.

*	**Moxa on Needle:** Attaching a moxa pole to the handle of a needle inserted at **ST 34** or **ST 36** provides deep warming therapy.

*	**Electroacupuncture:** Can be very effective for pain relief. Electroacupuncture can be applied between pairs of local points (e.g., EX-LE4 to EX-LE5, or ST 34 to SP 10) using a low-frequency (2-10 Hz) setting to stimulate endogenous opioid release.

*	**Cupping or Bleeding Cupping:** Around the knee (e.g., on SP 10) can be used to strongly invigorate Blood and remove stasis, especially if there is visible congestion or pronounced tenderness.

*	**Lifestyle Advice:** The practitioner should counsel the patient:

*	**Temporary Activity Modification:** Avoid activities that aggravate the pain (deep squats, heavy impact) while focusing on low-load strengthening (e.g., straight leg raises, clamshells) as advised by a physiotherapist.

*	**Apply Heat:** Regular use of a warm pack on the knee can help ease stiffness and pain.

*	**Diet:** Avoid foods that contribute to Dampness (dairy, greasy, sugary foods).

\### Rationale

This approach is comprehensive:

1. **Local Points:** Target the site of pathology to reduce pain and inflammation directly.

2. **Distal Points on the Stomach and Spleen Channels:** These channels dominate the anterior knee. Treating them is essential for patellofemoral issues.

3. **Kidney Tonification:** Addresses the root degenerative process shown on the MRI.

4. **Dampness Resolution:** Directly targets the joint effusion and swelling.

5. **Moxibustion:** Provides the deep warmth needed to dry dampness, move stagnation, and alleviate chronic pain, which NSAIDs could only temporarily suppress.

By combining these elements, acupuncture aims to not only manage pain but also improve joint environment and function, potentially slowing the degenerative process and improving the patient's quality of life and ability to engage in her beloved activity of climbing, albeit likely in a modified way.

**Case 10**

Of course. This is a case of Functional Ankle Instability (FAI), a common condition characterized by a feeling of the ankle "giving way" and recurrent sprains, often without significant ligamentous damage. The acupuncture approach will focus on strengthening the joint, improving proprioception, resolving any lingering Qi and Blood stasis from previous injuries, and calming the Shen (spirit) which can be affected by the fear of re-injury.

**Important Disclaimer:** This is for educational and informational purposes only. Acupuncture is a complementary therapy and should be part of a comprehensive rehabilitation plan. While the patient cannot commit to systematic rehab currently, they should be encouraged to incorporate even basic stabilizing exercises. Treatment must be administered by a qualified practitioner.

---

\### TCM Pattern Differentiation

Based on the symptoms:

*	**Recurrent Instability for 1 year:** This is a chronic condition. In TCM, the Spleen governs the muscles and the Liver governs the tendons. Chronic instability suggests **Spleen Qi Deficiency** (failing to hold and stabilize the joint) and potential **Liver Blood Deficiency** (failing to nourish the tendons and ligaments).

*	**History of likely sprains (implied by instability):** Previous trauma leads to **Qi and Blood Stasis** in the channel. If this stasis is not completely resolved, it creates weakness and malnourishment in the area, making it prone to re-injury.

*	**Lack of time for rehab:** This often relates to stress and overwork, which can contribute to **Liver Qi Stagnation** and further deplete Spleen Qi.

**Primary TCM Diagnosis:** **Spleen Qi Deficiency with Qi and Blood Stasis in the channels of the ankle.** There is also likely an underlying **Liver involvement** affecting the tendons.

\### Treatment Principle

The strategy is to:

1. **Strengthen the Spleen and Tonify Qi** to stabilize the joint (root treatment).

2. **Invigorate Blood and Remove Stasis** in the local area to address old injuries.

3. **Strengthen tendons and bones** and regulate the affected channels.

4. **Calm the Spirit** to reduce anxiety about the ankle giving way.

\### Suggested Acupuncture Points

This prescription will include local ankle points, distal points on the affected channels, and points to address the systemic weakness.

\#### 1. Local and Peri-Articular Points (To regulate Qi and Blood in the ankle):

*	**ST 41 (Jiexi):** Stream point of the Stomach channel. Located at the front of the ankle joint. A key point for ankle pain, weakness, and dysfunction.

*	**BL 60 (Kunlun):** Located in the depression between the lateral malleolus and the Achilles tendon. Benefits the ankle and reduces stiffness.

*	**KI 3 (Taixi):** Source point of the Kidney channel. Located between the medial malleolus and the Achilles tendon. **Tonifies the Kidneys to strengthen bones,** a crucial point for chronic joint weakness.

*	**GB 40 (Qiuxu):** Source point of the Gallbladder channel. Located in the depression anterior and inferior to the lateral malleolus. Important for lateral ankle stability.

*	**Ah Shi Points:** Identify and needle any tender spots around the ligaments (especially the anterior talofibular and calcaneofibular ligaments) and peroneal tendons.

\#### 2. Distal Points (To treat the root and regulate channels from afar):

*	**ST 36 (Zusanli):** He-Sea point of the Stomach channel. **Master point for tonifying Qi and Blood.** Strengthens the Spleen and Stomach to benefit the muscles and provide overall stability. **Essential for this case.**

*	**SP 6 (Sanyinjiao):** Meeting point of the Spleen, Liver, and Kidney channels. **Nourishes Blood, strengthens the Spleen,** and benefits the lower leg.

*	**SP 5 (Shangqiu):** Jing-River point of the Spleen channel. A specific distal point for **pain and weakness of the ankle joint.**

*	**LV 3 (Taichong):** Source point of the Liver channel. **Soothes the Liver, regulates Qi,** and nourishes the tendons. The combination of LV 3 and LI 4 (the Four Gates) can help regulate overall Qi flow.

\#### 3. Points for Proprioception and Neuromuscular Control:

*	**Scalp Acupuncture:** The **Motor Area (Upper 1/5)** corresponding to the lower limb can be needled on the contralateral side to improve motor control and stability.

*	**GV 20 (Baihui):** Raises Yang Qi and benefits the brain. Can help with overall coordination and sense of balance.

\#### 4. Points to Calm the Spirit (Shen):

*	**HT 7 (Shenmen):** Source point of the Heart channel. Calms the spirit and is useful for anxiety related to the fear of the ankle giving way.

*	**Yintang (EX-HN3):** Calms the mind and reduces stress.

\### Proposed Treatment Plan & Adjunctive Therapies

*	**Frequency:** 1-2 times per week.

*	**Technique:**

*	Use a **tonifying method** on points like ST 36, SP 6, KI 3.

*	Use an **even method** on local ankle points.

*	**Electroacupuncture:** Can be very beneficial. Apply to local points (e.g., ST 41 to GB 40) using a low-frequency setting to stimulate the muscles and ligaments around the ankle, improving proprioception and strength.

*	**Moxibustion:** **Highly recommended.** Applying moxa to points like **ST 36** and **KI 3** will strongly tonify Qi and strengthen the bones. Moxa on local Ah Shi points warms the channel, expels Cold, and promotes healing.

*	**Tuina Massage:** The practitioner can perform Tui Na techniques around the ankle to relax muscles, break up adhesions, and improve circulation before needling.

*	**Crucial Advice for the Patient:** While time is limited, the patient MUST be given 2-3 simple exercises to do at home to reinforce the treatment. This is non-negotiable for overcoming FAI. Examples:

*	**Single-Leg Stance:** Stand on the left foot while brushing teeth.

*	**Towel Scrunches:** Use the toes to scrunch a towel on the floor.

*	**Resisted Ankle Inversion/Eversion:** Use a resistance band for simple strength exercises.

\### Rationale

This point prescription works by:

1. **Strengthening the foundational Qi** of the body (via ST 36, SP 6) to provide systemic support for joint stability.

2. **Directly targeting the ankle joint** with local points to improve circulation, reduce residual stiffness, and promote healing.

3. **Addressing the tendon and bone element** through the Liver and Kidney channels (LV 3, KI 3).

4. **Improving the neurological control** of the ankle through scalp and distal points.

The goal is to make the ankle feel more secure and supported, reducing the frequency of instability episodes. However, the best outcomes will be achieved if the patient can incorporate even minimal home-based rehabilitation to retrain the neuromuscular system. Acupuncture can significantly reduce the time needed for rehabilitation by preparing the body to respond better to exercise.

**Case 11**

Of course. This is a critical case of Sudden Sensorineural Hearing Loss (SSNHL) with tinnitus, a condition that requires an aggressive and targeted acupuncture approach. The lack of response to high-dose corticosteroids indicates a severe presentation, but acupuncture can still be a powerful intervention, especially when focused on promoting neuro-regeneration and circulation.

**Important Disclaimer:** This is for educational and informational purposes only. SSNHL is a medical emergency. Acupuncture should be considered an adjunctive therapy, and the patient should remain under the care of an ENT specialist. Treatment must be administered by a practitioner experienced in treating neurological and otological conditions.

---

\### TCM Pattern Differentiation

Based on the symptoms and onset:

*	**Sudden Onset after Viral Infection (COVID-19):** A sudden, acute event points to **Internal Wind**. A viral infection is seen as a **Toxic Heat** pathogen that can rise and disturb the orifices.

*	**Complete Hearing Loss & Tinnitus (Cicada-like):** The ears are the orifice of the **Kidneys** ("Kidneys open into the ears"). A sudden loss suggests this pathogenic factor has blocked the clear orifices. Cicada-like tinnitus is often associated with **Liver Fire** or **Liver Yang Rising**.

*	**Lack of Response to Steroids:** Suggests a profound obstruction or deficiency that requires a strong regulatory approach.

*	**Psychological Shock:** The emotional impact can further disrupt the flow of Qi, particularly affecting the **Liver** (which regulates the smooth flow of Qi) and the **Heart** (which houses the spirit).

**Primary TCM Diagnosis:** **Liver and Gallbladder Fire Blazing upward** or **Wind-Phlegm obstructing the orifices** secondary to a viral toxin, with a likely underlying **Kidney Yin Deficiency**.

\### Treatment Principle

The strategy must be aggressive and dual:

1. **Drain Fire, Subdue Yang, and Clear the Orifices** (to address the acute, excess branch).

2. **Nourish Kidney Yin and Benefit the Ears** (to address the potential root deficiency).

3. **Regulate the Shaoyang Channel** (the pathway connecting the ear).

4. **Calm the Spirit** (to address shock and anxiety).

\### Suggested Acupuncture Points

Treatment should be frequent and stimulation strong, especially in the early stages.

\#### 1. Local Points Around the Ear (To clear the orifice):

*	**SJ 17 (Yifeng):** Located behind the earlobe. A primary point for treating disorders of the ear, it benefits the ear and expels Wind.

*	**SJ 21 (Ermen):** Directly in front of the ear. Opens the ear orifice.

*	**GB 2 (Tinghui):** Anterior to the intertragic notch. Benefits the ear and clears Shaoyang channel heat.

*	**SI 19 (Tinggong):** In front of the tragus. Benefits the ear and clears the orifice.

\#### 2. Distal Points on Limb Channels (To regulate Qi, drain fire, and nourish the root):

*	**SJ 3 (Zhongzhu):** The Shu-Stream point of the San Jiao channel. **Strongly benefits the ears** and clears heat from the Shaoyang channel.

*	**GB 43 (Xiaxi):** The Ying-Spring point of the Gallbladder channel. **Clears Liver and Gallbladder Fire** and is a key distal point for tinnitus and hearing loss.

*	**LI 4 (Hegu) + LV 3 (Taichong):** The "Four Gates." This powerful combination strongly **regulates Qi and Blood** throughout the body, subdues Yang, and calms the spirit. Critical for moving stagnation.

*	**KI 3 (Taixi):** Source point of the Kidney channel. **Tonifies Kidney Essence** to nourish the ears and address the root.

*	**PC 6 (Neiguan):** Calms the spirit, regulates the Liver, and helps manage the shock and emotional distress.

\#### 3. Scalp Acupuncture (CRUCIAL for Neurological Recovery):

*	**Temporal 3 Needle (Second Speech Area):** This area, located over the temporal region superior to the pinna, is used for auditory disorders and tinnitus. Needle on the affected side.

*	**Balance Area (Cerebellar Brainstem Area):** Located below the external occipital protuberance. Can help with vertigo and balance issues often associated with SSNHL.

\### Proposed Treatment Plan

*	**Frequency:** **Daily or every other day treatment is imperative.** The first few weeks are critical for recovery in SSNHL.

*	**Technique:**

*	Use a **strong reducing method** on points to clear heat and fire (GB 43, LI 4, LV 3).

*	Use an **even method** on local ear points.

*	Use a **tonifying method** on KI 3 to nourish the root.

*	**Electroacupuncture:** **Highly recommended.** Apply to local points (e.g., SJ 17 to SJ 21) using a low-frequency, dense-disperse wave (2/10Hz or 2/100Hz) to stimulate the auditory nerve and promote neuroplasticity.

*	**Retention Time:** 30-45 minutes.

*	**Moxibustion:** Generally **contraindicated** in this acute, heat/fire presentation. If any moxa is used, it would be only on KI 3 with a mild, tonifying technique, but caution is advised.

\### Rationale

This approach is designed to:

1. **Stimulate the auditory nerve and brain centers** directly through local and scalp acupuncture to encourage repair and rewiring (neuroplasticity).

2. **Reduce inflammation and "heat"** (the inflammatory response) from the affected area by draining the Liver and Gallbladder channels.

3. **Address the potential viral trigger** by clearing toxic heat.

4. **Provide the foundational nourishment** (via the Kidneys) needed for long-term healing of the delicate structures of the inner ear.

5. **Manage the significant emotional component** which can be a major barrier to recovery.

Given the poor prognosis from the initial medical treatment, this aggressive and targeted acupuncture protocol offers a viable and potentially effective complementary approach. The patient must be advised that consistent, frequent treatment is essential, and while improvement can occur, outcomes are variable. Management of expectations alongside strong emotional support is a key part of the therapy.

**Case 12**

Of course. This is an extremely delicate and complex palliative care case. The goal of acupuncture here is not to treat the cancer, but to provide comfort, manage the debilitating side effects of high-dose morphine, and improve the patient's quality of life in his final days.

**Extremely Important Disclaimer:** This is for educational and informational purposes only. This is an end-of-life care scenario. Acupuncture must be performed with extreme caution due to the patient's frailty, thrombocytopenia (low platelets), and compromised state. Treatment should be very gentle, non-invasive, and focus on distal points. It is a complementary therapy to be used alongside, not in place of, standard palliative medical care.

---

\### TCM Pattern Differentiation

Based on the symptoms and context:

*	**Severe Nausea & Vomiting:** Caused by morphine and the cancer itself. This is seen as **Rebellious Stomach Qi** (the normal downward flow of Stomach Qi is reversed).

*	**Weakness, Bone Marrow Suppression (low WBC/Platelets):** This represents a profound **Qi and Blood Deficiency**. The Bone Marrow is related to **Kidney Essence** in TCM; chemotherapy is seen as deeply draining to Essence.

*	**Cancer Pain:** Severe, fixed pain is a classic sign of **Blood Stasis**.

*	**Advanced Cachexia, Inability to Eat:** Represents **Spleen and Stomach Qi Deficiency** to an extreme degree; the digestive system has collapsed.

*	**Overall Condition:** A combination of **Severe Deficiency (Qi, Blood, Yin, Yang, Essence)** with a secondary **Excess (Toxic Stasis and Rebellious Qi)**.

\### Treatment Principle

The strategy is purely palliative and must be gentle:

1. **Harmonize the Stomach and Descend Rebellious Qi** (to reduce nausea and vomiting).

2. **Tonify Qi and Blood** very gently (to address weakness and support the body's minimal energy).

3. **Calm the Spirit (Shen)** and alleviate suffering (to provide comfort and ease anxiety).

\### Suggested Acupuncture Points

**Technique:** **Use very few needles. Use gentle, tonifying technique with no strong stimulation. Do not retain needles for long periods.** **Seirin**-type press needles could be an excellent option for continuous mild stimulation without the need for retention. **Absolutely avoid any points that strongly move Qi or Blood (e.g., SP 10, LI 4 strongly) due to the risk of bleeding from thrombocytopenia.**

\#### 1. Primary Points for Nausea and Vomiting:

*	**PC 6 (Neiguan):** **The single most important point.** Master point for harmonizing the Stomach, stopping nausea and vomiting, and calming the spirit. Easily accessible on the forearm.

*	**ST 36 (Zusanli):** He-Sea point of the Stomach. **Tonifies Qi** and harmonizes the Stomach to help with digestion and weakness. Needle very gently on the right side only (to avoid the affected pancreas area on the left) or bilaterally with extreme caution.

*	**CV 12 (Zhongwan):** Front-Mu point of the Stomach. Harmonizes the Stomach and descends Qi. **Do not needle deeply due to ascites/cachexia.** Use very shallow needling or non-invasive techniques like laser acupuncture or acupressure.

\#### 2. Points to Gently Tonify Qi and Blood:

*	**SP 6 (Sanyinjiao):** Meeting point of Spleen, Liver, and Kidney channels. Gently nourishes Blood and Yin. **Contraindicated in this case due to extremely low platelets and risk of bleeding.** **AVOID NEEDLING.** Acupressure only if deemed safe.

*	**LV 8 (Ququan):** He-Sea point of the Liver channel. Can be used as an alternative to SP 6 to nourish Blood gently.

*	**KI 3 (Taixi):** Source point of the Kidney. Gently tonifies Kidney Qi and Essence to address the root deficiency.

\#### 3. Points to Calm the Spirit and Alleviate Suffering:

*	**HT 7 (Shenmen):** Source point of the Heart channel. Calms the spirit, reduces anxiety and restlessness, which is crucial for a patient in distress.

*	**Yintang (EX-HN3):** Located between the eyebrows. Profoundly calms the mind and alleviates emotional suffering.

*	**Auricular (Ear) Acupuncture:** This is **highly recommended** as it is minimally invasive and effective. Use small press needles or vaccaria seeds on the **Shenmen, Stomach, Sympathetic, and Subcortex** points to help with nausea, pain, and relaxation.

\### Proposed Treatment Plan

*	**Frequency:** Daily or twice daily, as needed for symptom control.

*	**Technique:**

*	**Use 0.16x15mm or 0.12x15mm needles.**

*	**Insert needles very shallowly.**

*	**Use no or very minimal stimulation.**

*	**Retain for only 10-15 minutes** or use press needles for longer, gentle stimulation.

*	**Focus on distal points (PC 6, HT 7, ST 36) and auricular points.**

*	**Non-Invasive Alternatives (Highly Advisable):**

*	**Acupressure:** Family members can be taught to apply gentle, continuous pressure to **PC 6** (on the wrist) to help manage nausea between acupuncture sessions.

*	**Laser Acupuncture:** A cold laser can be used to stimulate points like PC 6, ST 36, and CV 12 with zero risk of infection or bleeding. This is an ideal modality for this patient.

*	**Moxibustion:** **Contraindicated.** The patient has a heat condition (cancer) and is extremely weak; moxa could be overly stimulating and is not advised.

\### Rationale

This protocol is designed for safety and comfort:

1. **PC 6** is proven to affect the vomiting center in the brainstem and is the cornerstone of anti-emetic acupuncture.

2. **Gentle tonification** with ST 36 and KI 3 aims to provide a tiny amount of energy support without draining the patient.

3. **Calming the Spirit** with HT 7 and Yintang is perhaps the most important goal, helping to ease the fear, anxiety, and restlessness that dominate the end-of-life experience.

4. **Auricular acupuncture** provides a continuous, low-level treatment that can help modulate the nervous system's response to pain and nausea.

The aim is to provide a few moments of relief, reduce the intensity of the morphine's side effects, and help the patient feel more peaceful. This is a profound act of compassion and support for both the patient and his family.

**Case 13**

Of course. This is a case series of Post-Prostatectomy Urinary Incontinence (PPUI) in elderly male patients. The acupuncture approach will focus on strengthening the sphincter, improving bladder control, and addressing the underlying deficiency that is common in this age group and after major surgery.

**Important Disclaimer:** This is for educational and informational purposes only. Acupuncture is a complementary therapy. Treatment must be administered by a qualified practitioner. Patients should be thoroughly evaluated to rule out other causes of incontinence.

---

\### TCM Pattern Differentiation

Based on the symptoms and patient history:

*	**Stress Incontinence (leakage with activity, coughing, sneezing):** This is a classic sign of **Qi Deficiency**, specifically **Middle Qi Deficiency**. The Spleen Qi is responsible for "holding" things in place; when it is weak, it fails to hold the urine.

*	**Status Post-Radical Prostatectomy:** Surgery is considered a significant trauma that can damage Qi and Blood, leading to **Qi Deficiency** and **Blood Stasis**.

*	**Advanced Age (mean ~68 years):** Naturally suggests an underlying **Kidney Qi Deficiency**. The Kidney governs the "gate" of the bladder (二便之司); its deficiency directly leads to an inability to control urination.

*	**Chronic Duration (>12 months):** Confirms a chronic condition rooted in deficiency.

**Primary TCM Diagnosis:** **Kidney Qi Deficiency and Spleen Qi Deficiency with Sinking of Middle Qi**, leading to a failure to contain and control fluids.

\### Treatment Principle

The strategy is to:

1. **Tonify and Raise Qi** (especially Spleen and Kidney Qi) to hold the urine.

2. **Strengthen the Kidney's ability to control the Bladder.**

3. **Regulate the Bladder function** locally.

\### Suggested Acupuncture Points

This prescription combines local points to strengthen the pelvic floor, distal points to tonify Qi, and points to regulate the Lower Jiao.

\#### 1. Core Points to Tonify Qi and Raise the Sinking:

*	**CV 6 (Qihai):** Sea of Qi. **Strongly tonifies Yuan (Source) Qi** throughout the body. This is a primary point for any type of Qi deficiency, including incontinence.

*	**CV 4 (Guanyuan):** Gate to the Original Qi. **Tonifies Kidney Qi and Kidney Yang** and strengthens the body's fundamental energy. It is a key point for the lower jiao and urinary function.

*	**ST 36 (Zusanli):** He-Sea point of the Stomach channel. **Master point for tonifying Qi and Blood.** Strengthens the Spleen and Stomach to produce more Qi, which is essential for addressing the root cause.

*	**SP 6 (Sanyinjiao):** Meeting point of the Spleen, Liver, and Kidney channels. **Tonifies the Spleen and Kidneys** and benefits the lower jiao.

\#### 2. Local Points for the Pelvic Floor and Bladder:

*	**CV 3 (Zhongji):** Front-Mu point of the Bladder. **Regulates the Bladder** and strengthens urinary control.

*	**BL 28 (Pangguangshu):** Back-Shu point of the Bladder. Works in combination with CV 3 (a Mu-Shu point combination) to regulate bladder function.

*	**BL 32 (Ciliao) or BL 33 (Zhongliao):** Points adjacent to the sacral foramina. These points directly influence the sacral nerves (S2-S4) that control the pelvic floor, bladder, and urethral sphincter. **Electroacupuncture on these points is particularly effective.**

*	**BL 35 (Huiyang):** Strengthens the Yang Qi of the kidney and benefits the lower jiao.

\#### 3. Points to Tonify the Kidneys (Root Treatment):

*	**KI 3 (Taixi):** Source point of the Kidney channel. **Tonifies Kidney Yin and Yang.**

*	**BL 23 (Shenshu):** Back-Shu point of the Kidney. **Tonifies Kidney Qi and Essence.**

\### Proposed Treatment Plan & Adjunctive Therapies

*	**Frequency:** 2-3 times per week. Consistency is key for chronic conditions.

*	**Technique:** Use a **tonifying method** on all points, especially CV 4, CV 6, ST 36, KI 3.

*	**Moxibustion is HIGHLY recommended:** Moxa is extremely important for treating Qi deficiency. It should be applied to **CV 4, CV 6, ST 36, and BL 23** to warm and tonify Yang Qi.

*	**Electroacupuncture:** This is **highly effective** for this condition. Apply a low-frequency (2-10 Hz) current between pairs of points to stimulate the pelvic floor muscles and nerves. Effective pairs include:

*	**CV 3 - CV 4**

*	**BL 32 - BL 33** (on one side or bilaterally)

*	**A local point (e.g., CV 3) and a distal point (e.g., SP 6)**

*	**Lifestyle Advice:** The practitioner should counsel the patients on:

*	**Kegel Exercises:** While needles are retained (especially with electroacupuncture), have the patient practice gentle pelvic floor contractions. This helps retrain the brain-muscle connection.

*	**Diet:** Avoid cold, raw foods and drinks which damage Spleen Yang. Eat warm, cooked, easily digestible foods.

*	**Avoid Straining:** Counsel on preventing constipation to avoid further downward pressure on the pelvic floor.

\### Rationale

This approach works by:

1. **Systemically Tonifying Qi:** Points like ST 36, CV 6, and CV 4 address the underlying Qi deficiency that is causing the incontinence.

2. **Strengthening the Kidneys:** Points like KI 3 and BL 23 directly improve the Kidney's ability to "gate" the bladder.

3. **Neuromuscular Stimulation:** Local points like CV 3, BL 32, and BL 33, especially with electroacupuncture, provide direct stimulation to the pudendal nerve and pelvic floor muscles, improving tone and control.

4. **Combining TCM and Modern Neurology:** This protocol integrates the TCM concept of Qi deficiency with modern understanding of innervation of the pelvic floor, making it a powerful approach for PPUI.

Given the patients' age and refusal of more invasive options, this conservative, holistic approach is well-suited to improve their quality of life by reducing the frequency and volume of urinary leakage.

**Case 14**

Of course. This is a classic and complex case of Vestibular Migraine (VM). The acupuncture approach will focus on regulating the Liver and Gallbladder, extinguishing internal Wind, resolving Phlegm, and calming the Spirit, which are the core TCM patterns underlying this condition.

**Important Disclaimer:** This is for educational and informational purposes only. Acupuncture is a complementary therapy and should be integrated into a comprehensive management plan. Treatment must be administered by a qualified practitioner.

---

\### TCM Pattern Differentiation

Based on the symptoms:

*	**Recurrent Rotational Vertigo:** Vertigo is primarily attributed to **Internal Wind** and **Phlegm-Dampness** obstructing the clear orifices of the head. The Liver is often the source of Internal Wind.

*	**Nausea:** A clear sign of **Rebellious Stomach Qi**, often caused by Phlegm-Dampness obstructing the Middle Jiao (Spleen and Stomach).

*	**Photophobia & Phonophobia:** Extreme sensitivity suggests **Liver Fire** or **Liver Yang Rising**.

*	**Migrainous Headaches (Temporal Region):** The temporal area is governed by the **Shaoyang Gallbladder Channel**. This confirms the involvement of the Liver-Gallbladder system.

*	**Vertigo and Headaches Not Simultaneous:** Suggests a shared underlying root (e.g., Liver Wind) that manifests in different ways.

*	**Weight Gain from Medication:** Sodium valproate can exacerbate **Spleen Deficiency** and **Phlegm-Dampness**, which is a key pathological factor in vertigo.

**Primary TCM Diagnosis:** **Liver Yang Rising / Wind-Phlegm Harassing the Upper Orifices,** with an underlying **Spleen Deficiency** leading to Phlegm formation.

\### Treatment Principle

The strategy is to:

1. **Pacify the Liver and Subdue Yang** (to address the root of the wind and rising energy).

2. **Extinguish Wind and Transform Phlegm** (to clear the obstruction from the head).

3. **Harmonize the Stomach and Stop Vomiting** (to alleviate nausea).

4. **Regulate the Shaoyang Channel** (the primary channel involved).

5. **Calm the Spirit (Shen)** to reduce sensory hypersensitivity.

\### Suggested Acupuncture Points

\#### 1. Points to Subdue Liver Yang and Extinguish Wind:

*	**LV 3 (Taichong):** **Source point of the Liver channel.** The primary point to pacify the Liver, subdue Yang, and extinguish Wind. Essential for both headaches and vertigo.

*	**GB 20 (Fengchi):** Expels Wind (internal and external), benefits the head and eyes, and is a master point for treating vertigo and headaches.

*	**LI 11 (Quchi):** He-Sea point. Clears Heat and subdues Yang.

\#### 2. Points to Resolve Phlegm and Harmonize the Stomach:

*	**ST 40 (Fenglong):** **Luo-Connecting point of the Stomach channel. The key point to resolve Phlegm and Dampness** throughout the body. Crucial for the phlegm component of vertigo.

*	**ST 8 (Touwei):** The meeting point of the Stomach and Gallbladder channels. Specific for treating headaches with a temporal distribution and vertigo.

*	**PC 6 (Neiguan):** Luo-Connecting point of the Pericardium. **Harmonizes the Stomach, stops nausea and vomiting,** and calms the spirit.

\#### 3. Points to Regulate the Shaoyang Channel (Gallbladder):

*	**GB 5 (Xuanlu) & GB 6 (Xuanli):** Points on the temporal region that regulate the Gallbladder channel and are specific for migrainous headaches in this area.

*	**SJ 5 (Waiguan):** Connects to the Yang Linking Vessel. A key distal point for regulating the Shaoyang channel and treating disorders of the side of the head and vertigo.

*	**GB 43 (Xiaxi):** Ying-Spring point of the Gallbladder channel. Clears heat from the channel and is effective for headaches and vertigo.

\#### 4. Points to Calm the Spirit and Benefit the Head:

*	**GV 20 (Baihui):** Raises clear Yang (if due to deficiency) but also has a strong calming effect and benefits the brain. Used to clear the senses.

*	**EX-HN3 (Yintang):** Located between the eyebrows. Calms the mind and reduces stress and anxiety, which are common triggers for VM.

*	**HT 7 (Shenmen):** Source point of the Heart channel. Calms the spirit and is useful for the hypersensitivity (photophobia, phonophobia).

\### Proposed Treatment Plan

*	**Frequency:** Begin with 2 treatments per week. The goal is to reduce frequency and severity of attacks. During an acute attack, treatment can be more frequent.

*	**Technique:**

*	Use a **reducing or even method** on points to subdue Yang and clear phlegm (LV 3, GB 20, ST 40).

*	Use an **even method** on calming points (GV 20, Yintang, HT 7).

*	**Retention Time:** 20-30 minutes.

*	**Electroacupuncture:** May be applied to points on the temporal region (e.g., GB 5 to GB 6) for headache and vertigo control, using a low-frequency setting.

*	**Lifestyle & Dietary Advice (CRUCIAL):**

*	**Diet:** Strongly advise against foods that generate Phlegm and dampness (dairy, greasy, sugary foods) and foods that promote Liver Yang (alcohol, caffeine, spicy foods).

*	**Sleep:** Encourage regular sleep patterns, as lack of sleep is a major trigger for Liver Yin deficiency and Yang rising.

*	**Stress Management:** Recommend techniques like meditation, Qi Gong, or yoga to manage stress, a primary driver of Liver Qi stagnation transforming into Yang rising.

\### Rationale

This prescription addresses VM from multiple angles:

1. **LV 3 and GB 20** form a core pair to pacify the Liver and extinguish the Internal Wind causing the vertigo.

2. **ST 40** directly addresses the Phlegm component that contributes to the feeling of fullness, dizziness, and nausea.

3. **PC 6** is specific for the nausea that accompanies the vertigo attacks.

4. **SJ 5 and local GB points** target the specific Shaoyang channel pathology responsible for the temporal headaches.

5. **Calming points** help reduce the nervous system hypersensitivity that characterizes this condition.

By targeting the root (Liver) and the branch (Phlegm, Wind, Rebellious Qi), acupuncture can be highly effective in reducing the frequency, duration, and intensity of vestibular migraine attacks, providing a valuable drug-free management strategy.

ChatGPT EN

**Case 1**

Thank you for sharing the detailed case presentation. Based on the information:

* **Patient:** 23-year-old male

* **Main complaint:** Discomfort and pain in both lower limbs for 2 years, **worse at night**, **relieved by movement/walking**

* **Diagnosis:** Restless Legs Syndrome (RLS)

* **Treatment choice:** Acupuncture (no medication)

In Traditional Chinese Medicine (TCM), RLS is often related to **Kidney Yin deficiency with empty heat**, **Liver blood deficiency with wind**, or **Qi and blood stagnation in the channels of the lower limbs**. Acupuncture point selection usually aims to **nourish Liver and Kidney, invigorate blood, calm the Shen, and unblock channels**.

\### Suggested Acupuncture Point Prescription for RLS

**Primary points:**

* **GB34 (Yanglingquan):** Benefits sinews, relieves leg discomfort.

* **ST36 (Zusanli):** Tonifies Qi and blood, strengthens lower limbs.

* **SP6 (Sanyinjiao):** Nourishes Liver, Spleen, Kidney; calms the Shen.

* **KI3 (Taixi):** Tonifies Kidney Yin and Yang, anchors Qi.

* **LR3 (Taichong):** Pacifies Liver wind, nourishes blood.

**Secondary/supportive points (based on pattern):**

* **BL23 (Shenshu):** Tonifies Kidney.

* **BL18 (Ganshu):** Regulates Liver, nourishes blood.

* **HT7 (Shenmen):** Calms the mind, improves sleep.

* **DU20 (Baihui):** Calms Shen, lifts Yang.

* **Anmian (extra point):** For insomnia and night symptoms.

**Local/trigger points (if pain is significant):**

* **Ashì points** along the calves and thighs.

* **BL57 (Chengshan)** and **BL60 (Kunlun):** For leg pain, cramping, and restlessness.

---

👉 In clinical practice, a typical protocol for RLS might include a **combination of systemic tonifying points (ST36, SP6, KI3, LR3) with local points (BL57, GB34)** and calming points (HT7, Anmian, DU20).

Would you like me to create a **sample treatment plan** (e.g., number of sessions per week, needle retention time, and needling method), tailored for this case?

**Case 2**

Thank you for the detailed case. Let’s summarize before suggesting acupuncture points:

---

\### **Case Summary**

* **Patient:** 79-year-old female

* **Symptoms:** Neck and shoulder pain, numbness/pain in left upper limb, weakness in both lower limbs, unstable gait (“walking on cotton”), tendency to fall.

* **Exam findings:** Neck stiffness, tenderness, positive neck compression test, decreased left biceps/triceps reflexes, positive Hoffmann’s and Babinski’s signs.

* **Imaging:** Calcification of posterior longitudinal ligament (C4–C6), dural sac & C6 nerve root compression, disc protrusion at C5–C6.

* **Diagnosis:** Cervical ossification of the posterior longitudinal ligament (C-OPLL).

* **Treatment choice:** Acupuncture (patient declined surgery).

---

\### **TCM Perspective**

This case reflects:

* **Bi syndrome / obstruction in the channels** due to stagnation of Qi and Blood in the cervical region.

* **Underlying Liver–Kidney deficiency** contributing to weakness in the legs and unsteady gait.

* **Phlegm and Blood stasis** blocking the meridians in the cervical spine.

Treatment principle:

* Relax tendons and collaterals, relieve pain.

* Promote Qi and Blood circulation in the cervical region.

* Nourish Liver and Kidney to strengthen lower limbs and gait.

* Support neurological function and calm the Shen.

---

\### **Suggested Acupuncture Point Prescription**

**Local / symptomatic points (cervical and shoulder region):**

* **GB20 (Fengchi):** Relieves neck stiffness, dizziness, and gait imbalance.

* **GV14 (Dazhui):** Clears channels at cervical level, strengthens Yang.

* **Jiaji points (EX-B2) at C4–C6:** Directly regulate local nerve root compression.

* **SI15 (Jianzhongshu) & SI16 (Tianchuang):** For neck–shoulder pain and channel obstruction.

* **LI15 (Jianyu) & LI11 (Quchi):** Promote circulation, benefit upper limb numbness/pain.

**Distal points for pain/numbness in upper limb:**

* **LI4 (Hegu):** Clears channels, relieves pain.

* **TE5 (Waiguan):** Opens Yang Linking Vessel, benefits neck and shoulder.

* **LU7 (Lieque):** Regulates cervical spine, benefits upper limb.

**Points for lower limb weakness and gait instability:**

* **ST36 (Zusanli):** Tonifies Qi and Blood, strengthens legs.

* **GB34 (Yanglingquan):** Benefits tendons, improves motor function.

* **SP6 (Sanyinjiao):** Tonifies Liver/Kidney, supports lower limbs.

* **KI3 (Taixi):** Tonifies Kidney to strengthen bone and marrow.

**Supporting points for neurological signs / calming Shen:**

* **DU20 (Baihui):** Improves balance, calms Shen.

* **HT7 (Shenmen):** Calms mind, reduces neurological hyperactivity.

---

\### **Key Notes**

* **Needling method:** Gentle manipulation (especially given age), mild reinforcing–reducing.

* **Needle retention:** 20–30 minutes.

* **Frequency:** 2–3 times per week, depending on tolerance.

* **Adjuncts:** Moxibustion at ST36 and DU14 may help tonify Qi–Yang in elderly patients.

---

👉 This point combination aims to **relieve neck–shoulder pain, reduce nerve root compression symptoms, improve limb strength, and stabilize gait** while addressing the patient’s age-related deficiency.

Would you like me to also suggest a **sample staged treatment protocol** (e.g., acute pain relief phase vs. functional rehabilitation phase) for this patient?

**Case 3**

Got it — thanks for sharing this case. Let’s unpack it carefully before suggesting acupuncture points.

---

\### **Case Summary**

* **Patient:** 66-year-old female

* **History:** >10 years of generalized muscle weakness & soreness.

* **Recent exacerbation:** Severe tearing pain in limbs (esp. lower limbs), triggered after catching a cold.

* **Labs:** CK \~900 U/L (supports inflammatory muscle disease).

* **Diagnosis:** Polymyositis (PM).

* **Past treatment:** Prednisone + celecoxib (partial improvement but frequent relapses).

* **Current situation:** Relapsed, ongoing weakness + limb pain; declines further drug therapy due to side-effect concerns.

* **Plan:** Acupuncture for symptom relief.

---

\### **TCM Interpretation**

* **Main patterns:**

* **Deficiency of Qi & Blood** → chronic weakness.

* **Spleen & Kidney deficiency** → muscle atrophy/weakness.

* **Wind-cold-damp invasion** after catching cold → acute worsening, limb pain.

* **Stasis in channels** → tearing pain in muscles/limbs.

**Treatment principle:**

* Tonify Qi and Blood, strengthen Spleen and Kidney.

* Expel wind-cold-damp, unblock meridians.

* Relieve muscle pain, restore strength, calm spirit.

---

\### **Suggested Acupuncture Point Prescription**

**1. Core points (tonify Qi & Blood, strengthen muscles):**

* **ST36 (Zusanli):** Tonifies Qi & Blood, strengthens body/muscles.

* **SP6 (Sanyinjiao):** Tonifies Spleen/Kidney, harmonizes Yin–Blood.

* **BL20 (Pishu):** Strengthens Spleen, improves muscle function.

* **BL23 (Shenshu):** Tonifies Kidney essence for bone/muscle support.

* **CV6 (Qihai) & CV4 (Guanyuan):** Reinforce Qi and Kidney essence.

**2. Points for muscle pain & soreness:**

* **GB34 (Yanglingquan):** Benefits sinews, relieves pain.

* **LI4 (Hegu) + LR3 (Taichong) \[Four Gates]:** Move Qi and Blood, relieve pain.

* **Ashi points** on affected muscles (local needling to relieve pain/stiffness).

**3. Points for limb weakness (esp. lower limbs):**

* **GB39 (Xuanzhong):** Nourishes marrow, strengthens tendons.

* **KI3 (Taixi):** Tonifies Kidney essence, benefits lower limbs.

* **DU14 (Dazhui):** Tonifies Yang, clears external pathogens (especially post-cold).

**4. Points for relapse prevention / calming system:**

* **DU20 (Baihui):** Lifts Yang, stabilizes gait, calms spirit.

* **HT7 (Shenmen):** Calms Shen, relieves emotional burden of chronic disease.

---

\### **Notes**

* **Needling method:**

* Gentle reinforcing at tonifying points (ST36, SP6, BL20, BL23, CV4/6).

* Reducing/dispersing at points for pain (GB34, LI4, LR3, ashi).

* **Needle retention:** 25–30 minutes.

* **Frequency:** 2–3 times per week during acute flare; 1–2 times per week for maintenance.

* **Adjuncts:** Moxibustion at ST36, CV4, BL23 in elderly with cold-induced flare.

---

✅ This protocol addresses both the **chronic deficiency (muscle weakness)** and the **acute excess (pain after cold invasion)**, while aiming for long-term prevention of relapses.

Would you like me to also sketch out a **staged treatment plan** (e.g., acute flare phase vs. maintenance phase) like I did in the cervical OPLL case?

**Case 4**

Thank you for sharing this very detailed case. Let’s carefully analyze it before suggesting acupuncture.

---

\### **Case Summary**

* **Patient:** 57-year-old man

* **Course:** 78 days of limb weakness & gait instability.

* **Neurological findings:**

* Aspiration risk (concealed aspiration, weakened gag reflex).

* Bilateral weakness (L > R).

* Ataxia, proprioceptive disturbance (esp. right limbs).

* Mild dysarthria.

* **Scores:**

* FMA: 96 (motor impairment, mild-moderate).

* BI: 80 (needs some assistance with daily living).

* MMSE: 26 (mild cognitive impairment).

* Swallow test: profile III/score 4 (choking present).

* **Imaging:**

* T2 MRI: high signal in brainstem & thalami, “Mickey Mouse ear sign” → **suggestive of brainstem infarction / ischemia**.

* MRA: severe stenosis/occlusion in vertebral & basilar arteries.

* Angiography confirmed severe BA stenosis + VA occlusion.

* **Diagnosis:** Posterior circulation ischemic stroke (brainstem & thalami) with dysphagia, limb weakness, gait ataxia, and mild cognitive impairment.

---

\### **TCM Interpretation**

* **Pattern:**

* Wind-phlegm obstructing channels (sudden onset stroke signs).

* Qi deficiency + Blood stasis → weakness, prolonged recovery.

* Phlegm-damp blocking throat → dysphagia.

* Brain and marrow deficiency (Kidney, Liver) → cognitive impairment.

**Treatment principles:**

* Open orifices, extinguish internal wind, resolve phlegm.

* Promote Qi and Blood circulation in meridians.

* Restore speech/swallowing.

* Strengthen body, support recovery.

---

\### **Suggested Acupuncture Point Prescription**

**1. Core stroke-recovery points:**

* **DU20 (Baihui):** Raises Yang, restores consciousness, cognitive benefit.

* **DU16 (Fengfu) + DU14 (Dazhui):** Opens brain orifices, clears channels.

* **LI4 (Hegu) + LI11 (Quchi):** Activate Qi/Blood, benefit motor recovery.

* **ST36 (Zusanli):** Tonifies Qi, strengthens limbs.

* **SP6 (Sanyinjiao):** Harmonizes Yin, benefits lower limbs.

**2. Dysphagia-focused points:**

* **CV23 (Lianquan):** Benefits tongue, swallowing.

* **GB20 (Fengchi):** Clears wind, improves swallowing/gag reflex.

* **Jinjin & Yuye (EX-HN12/13):** Tongue root points for dysarthria/dysphagia.

* **LI17 (Tianding) + LI18 (Futu):** Local points for throat obstruction.

**3. Motor/ataxia rehabilitation points:**

* **GB34 (Yanglingquan):** Benefits tendons, improves coordination.

* **BL40 (Weizhong):** Activates channels, supports lower limb function.

* **KI3 (Taixi):** Nourishes Kidney essence, benefits gait stability.

* **SJ5 (Waiguan):** Opens Yang Linking Vessel, supports balance & limb function.

**4. Cognitive / mental function:**

* **DU24 (Shenting):** Calms Shen, improves memory.

* **HT7 (Shenmen):** Calms mind, helps cognition and speech recovery.

---

\### **Notes for Application**

* **Needling method:**

* Gentle reinforcing on tonifying points (ST36, SP6, KI3, DU20).

* Reducing/dispersing on phlegm/wind-clearing points (GB20, LI4, LI11, LI17/18).

* **Needle retention:** 20–30 min.

* **Frequency:** Ideally 5 times/week during early rehab, tapering to 2–3 times/week.

* **Adjuncts:** Electroacupuncture (EA) at LI4–LI11, ST36–SP6 may enhance motor recovery. Scalp acupuncture (motor/speech areas) is strongly recommended in stroke rehabilitation.

---

✅ This prescription addresses **limb weakness, ataxia, dysphagia, and mild cognitive impairment** by combining **systemic, local, and scalp acupuncture strategies**.

Would you like me to also propose a **scalp acupuncture map (motor, sensory, speech areas)** specifically tailored for this brainstem infarction case?

**Case 5**

Thank you for the clear presentation. Let’s analyze carefully.

---

\### **Case Summary**

* **Patient:** 42-year-old male

* **Onset:** Sudden left limb weakness + bilateral blurred vision (Aug 2022).

* **Initial Dx:** Posterior circulation cerebral infarction.

* **Treatment:** Dipyridamole, atorvastatin, plaque stabilization, simple rehab.

* **Residual symptom (1 month later):** Persistent or worsening blurred vision + reduced fields.

* **Ophthalmology:** Homonymous hemianopia (visual field defect).

* **Final Dx:** Post-stroke hemianopia.

* **Treatment chosen:** Acupuncture rehabilitation.

---

\### **TCM Interpretation**

* **Stroke etiology in TCM:**

* Wind, phlegm, and blood stasis obstructing channels of the brain and eyes.

* Deficiency of Qi/Blood and Liver–Kidney essence following stroke → failure to nourish eyes.

**Treatment principles:**

* Promote Qi and Blood circulation in the brain and eye channels.

* Open the orifices, clear obstruction of visual pathways.

* Nourish Liver and Kidney essence for vision.

* Support neurological recovery after stroke.

---

\### **Suggested Acupuncture Point Prescription**

**1. Local/ocular points (to stimulate optic nerve & circulation):**

* **BL1 (Jingming) + BL2 (Zanzhu):** Brighten vision, regulate eye circulation.

* **GB20 (Fengchi):** Improves posterior circulation, relieves eye/brain symptoms.

* **Taiyang (EX-HN5):** For blurred vision and visual field defects.

* **Yuyao (EX-HN4):** Eye strain, blurred vision.

**2. Scalp acupuncture (very important in hemianopia):**

* **Visual area (occipital line, 1 cm lateral to external occipital protuberance, 4 cm long):** Direct stimulation of visual cortex.

* **Posterior temporal line (language/visual association area):** If visual recognition affected.

**3. Systemic stroke-recovery points:**

* **DU20 (Baihui):** Opens brain orifices, raises clear Yang.

* **DU16 (Fengfu):** Regulates brain and marrow, treats stroke-related eye symptoms.

* **LI4 (Hegu) + LR3 (Taichong) \[Four Gates]:** Moves Qi/Blood, relieves channel obstruction.

* **ST36 (Zusanli):** Tonifies Qi and Blood, aids overall stroke recovery.

* **SP6 (Sanyinjiao):** Nourishes Yin, supports Liver–Kidney.

**4. Supportive points (for circulation and posterior circulation stroke):**

* **BL18 (Ganshu):** Nourishes Liver to benefit eyes.

* **KI3 (Taixi):** Tonifies Kidney essence, supports vision.

* **GB37 (Guangming):** Classic point for vision disorders, visual field deficits.

---

\### **Notes for Application**

* **Needling method:**

* Gentle reinforcing at tonifying points (ST36, SP6, KI3, BL18).

* Reducing/mild stimulation at points for circulation & phlegm-wind (GB20, Four Gates).

* **Needle retention:** 20–30 minutes.

* **Frequency:** 3–5 times per week in early rehab phase, taper as condition stabilizes.

* **Adjuncts:** Electroacupuncture at GB20–BL1 or GB37–ST36 has shown benefit in some stroke-vision rehab studies.

---

✅ This prescription combines **local eye points, scalp acupuncture, systemic stroke-recovery points, and Liver–Kidney nourishing points** to target **hemianopia after posterior circulation infarction**.

Would you like me to also prepare a **scalp acupuncture zone map (occipital/visual cortex areas)** that could be directly applied for this case?

**Case 6**

Thanks for the case details — let’s break it down carefully.

---

\### **Case Summary**

* **Patient:** 23-year-old Japanese male

* **Complaint:** Pain in right temporomandibular (TMJ) area + difficulty opening mouth (2 months).

* **Course:** Gradual worsening; pain with mouth opening, eating, and talking.

* **Exam:** Tenderness of right temporalis & masseter, bilateral trapezius & splenius capitis.

* **Dx (likely):** Temporomandibular disorder (TMD) with myofascial involvement.

---

\### **TCM Understanding**

* **Pattern:** Local Qi and Blood stagnation in the Stomach, Gallbladder, and Triple Energizer channels (which pass through the TMJ).

* Contributing factors: stress, muscle tension, possibly Liver Qi stagnation.

**Treatment principles:**

* Relieve pain by unblocking channels.

* Relax local muscles (masseter, temporalis, neck).

* Harmonize Qi in Stomach/Gallbladder channels.

---

\### **Suggested Acupuncture Point Prescription**

**Local points (for TMJ pain & jaw opening):**

* **ST6 (Jiache):** Major point for jaw pain, masseter spasm.

* **ST7 (Xiaguan):** TMJ pain, restricted mouth opening.

* **SI18 (Quanliao):** Relieves facial pain/tension.

* **LI4 (Hegu):** Local pain via distal action, opens channels in face/jaw.

* **Ashì points** at tender spots in temporalis & masseter.

**Neck/shoulder tension (secondary pain generators):**

* **GB20 (Fengchi):** Relieves neck stiffness, balances upper body Qi.

* **GB21 (Jianjing):** For trapezius tension.

* **BL10 (Tianzhu):** Benefits splenius capitis & upper cervical region.

**Systemic regulation:**

* **ST36 (Zusanli):** Tonifies Qi & Blood, harmonizes Stomach channel (related to jaw).

* **LR3 (Taichong):** Smooths Liver Qi, addresses stress-related jaw clenching.

* **TE5 (Waiguan):** Opens Yang Linking Vessel, harmonizes lateral head/neck.

---

\### **Notes**

* **Needling method:**

* Gentle local needling at ST6/ST7, shallow insertion due to facial anatomy.

* Disperse technique on Ashì points to relax muscle knots.

* Reducing on GB21/GB20 for neck muscle relaxation.

* **Needle retention:** 20 minutes.

* **Frequency:** 2–3 times per week initially, taper as pain subsides.

* **Adjuncts:**

* Local moxibustion or warm compress may help muscle relaxation.

* Jaw relaxation exercises + posture correction for long-term benefit.

---

✅ This point set addresses both the **local TMJ pain/dysfunction** and the **associated muscle tension in the neck/shoulder**, while also regulating systemic Qi flow.

Would you like me to also propose a **specialized electroacupuncture protocol** (e.g., pairing ST6–ST7 or GB20–GB21) that is often used for TMD cases?

**Case 7**

Thank you for the case details — let’s break this one down carefully.

---

\### **Case Summary**

* **Patient:** 38-year-old man

* **Diagnosis:** Androgenetic alopecia (AGA), >3 years duration.

* **Previous treatment:** Finasteride prescribed at community hospital, not effective.

* **Symptoms:** Hair loss, fine/weak hair (flat and greasy), itchy scalp, fatigue.

---

\### **TCM Understanding**

* **Patterns involved:**

* **Kidney essence deficiency** → failure to nourish hair (Kidney stores essence, essence generates hair).

* **Spleen Qi deficiency with damp-heat** → greasy, itchy scalp.

* **Qi and Blood deficiency** → thin/fine hair.

* **Liver Qi stagnation** may also contribute if fatigue/stress is chronic.

**Treatment principles:**

* Nourish Kidney essence, tonify Qi and Blood.

* Clear damp-heat from scalp, improve circulation to hair follicles.

* Relieve scalp itching, strengthen hair growth.

---

\### **Suggested Acupuncture Point Prescription**

**Local scalp points (improve circulation to hair follicles):**

* **DU20 (Baihui):** Lifts Yang, improves scalp circulation.

* **DU21 (Qianding) & DU22 (Xinhui):** Promote hair growth.

* **GB20 (Fengchi):** Clears wind, improves scalp microcirculation.

* **BL7 (Tongtian) & EX-HN1 (Sishencong):** Classic points for alopecia.

* **Ashì points** on affected scalp regions.

**Systemic points (nourish Kidney, Spleen, Liver, Blood):**

* **KI3 (Taixi):** Tonifies Kidney essence.

* **BL23 (Shenshu):** Strengthens Kidney, benefits hair.

* **ST36 (Zusanli):** Tonifies Qi and Blood, treats fatigue.

* **SP6 (Sanyinjiao):** Harmonizes Spleen, Liver, Kidney; nourishes Yin–Blood.

* **LR3 (Taichong):** Moves Liver Qi, regulates stress.

* **BL17 (Geshu):** Influential point of Blood → nourishes hair roots.

**For greasy scalp & itching (clear damp-heat):**

* **LI11 (Quchi):** Clears heat.

* **SP10 (Xuehai):** Cools Blood, relieves itching.

* **SJ5 (Waiguan):** Dispels wind-heat affecting scalp.

---

\### **Notes for Application**

* **Needling method:**

* Gentle reinforcing on ST36, KI3, BL23, SP6, BL17.

* Reducing on LI11, SP10, GB20 for damp-heat and itching.

* Scalp acupuncture along the **vertex and hairline regions** with mild stimulation.

* **Needle retention:** 20–30 minutes.

* **Frequency:** 2–3 times per week for at least 3 months (hair growth needs long-term regulation).

* **Adjuncts:**

* Plum-blossom tapping on scalp points around thinning regions (mild stimulation, not causing bleeding).

* Moxibustion at DU20 and BL23 if Kidney deficiency predominates (esp. fatigue, weak constitution).

---

✅ This prescription combines **local scalp points** to improve circulation and **systemic points** to treat underlying deficiency and damp-heat, addressing both **root (deficiency)** and **branch (hair/scalp symptoms)** of AGA.

Would you like me to also prepare a **specialized scalp acupuncture protocol** (with exact lines/zones) that has been reported for alopecia cases?

**Case 8**

Thanks for sharing this case. Let’s carefully work it through.

---

\### **Case Summary**

* **Patient:** 33-year-old man

* **Diagnosis:** Androgenetic alopecia (AGA), duration >2 years.

* **Course:** Gradual worsening hair loss at vertex (top of head).

* **No prior treatment.**

* **Symptoms:** Hair loss, itchy scalp, fatigue, anorexia.

---

\### **TCM Interpretation**

* **Kidney deficiency** → failure to nourish hair.

* **Spleen Qi deficiency** → poor transformation/transport → fatigue, anorexia, lack of nourishment to hair.

* **Blood deficiency + damp-heat in scalp** → itching, thinning hair.

**Treatment principles:**

* Tonify Spleen and Kidney, generate Qi and Blood.

* Nourish hair roots, improve circulation on scalp.

* Clear damp-heat, relieve itching.

---

\### **Suggested Acupuncture Point Prescription**

**Local scalp points (promote blood circulation, stimulate follicles):**

* **DU20 (Baihui):** Lifts Yang, nourishes head.

* **DU21 (Qianding), DU22 (Xinhui):** Stimulate vertex hair growth.

* **EX-HN1 (Sishencong):** Four extra points around DU20 for alopecia.

* **BL7 (Tongtian):** Opens orifices, benefits scalp.

* **Ashì points** at thinning/itchy areas.

**Systemic points (treat root deficiency):**

* **ST36 (Zusanli):** Tonifies Qi, improves appetite, strengthens digestion.

* **SP6 (Sanyinjiao):** Tonifies Spleen, Kidney, and Liver; nourishes Yin-Blood.

* **BL20 (Pishu):** Strengthens Spleen to resolve fatigue, anorexia.

* **BL23 (Shenshu):** Tonifies Kidney essence, supports hair growth.

* **KI3 (Taixi):** Tonifies Kidney Yin and essence.

* **LR3 (Taichong):** Moves Liver Qi, harmonizes with Spleen for digestion.

**For scalp itching / damp-heat signs:**

* **LI11 (Quchi):** Clears heat.

* **SP10 (Xuehai):** Cools and invigorates Blood.

* **SJ5 (Waiguan):** Expels wind-heat affecting head/scalp.

---

\### **Notes**

* **Needling method:**

* Reinforcing on ST36, SP6, BL20, BL23, KI3.

* Reducing/mild dispersing on LI11, SP10, SJ5, GB20 (if itching significant).

* Scalp points: mild stimulation with 15–20 min retention.

* **Adjuncts:**

* Plum-blossom tapping or dermal needle stimulation on vertex (daily or every other day).

* Moxibustion at DU20 and BL23 if Kidney deficiency predominates (fatigue, weak digestion).

* **Treatment frequency:** 2–3 times per week; minimum 3 months for observable results.

---

✅ This prescription addresses **both local scalp stagnation** and **underlying Spleen–Kidney deficiency with damp-heat**, aiming to reduce itching, strengthen digestion/energy, and nourish hair roots.

Would you like me to also create a **comparative point set** for the two AGA cases (the 38-year-old with greasy scalp vs. the 33-year-old with anorexia/fatigue), so you can see the differences in emphasis?

**Case 9**

Thank you for the clear and detailed case. Let’s carefully analyze it.

---

\### **Case Summary**

* **Patient:** 51-year-old woman, active climber.

* **Diagnosis:** Patellofemoral arthritis (PFA, left knee).

* **Course:** 1 year of pain, mainly lateral knee & subpatellar region.

* **Aggravated by:** Squatting, stairs.

* **Exam:** Positive patellofemoral abrasion test, knee space tenderness.

* **Imaging:** Cartilage degeneration, bone marrow edema, synovial thickening, joint effusion, mild soft tissue swelling.

* **Treatment history:** NSAIDs (temporary relief, recurrent pain).

* **Impact:** Pain interfering with daily life.

---

\### **TCM Interpretation**

* **Bi syndrome (painful obstruction)** due to wind-damp invasion with underlying Qi/Blood stagnation.

* **Kidney and Liver deficiency** → weak tendons/bones, predisposed to degeneration.

* **Damp-heat and Blood stasis in local joint** → swelling, pain, effusion.

**Treatment principles:**

* Dispel wind-damp, clear heat, reduce swelling/pain.

* Promote Qi and Blood circulation to the knee.

* Tonify Liver–Kidney to strengthen tendons and bones.

* Restore mobility and function.

---

\### **Suggested Acupuncture Point Prescription**

**Local knee points (pain relief, circulation):**

* **EX-LE4 (Neixiyan) + ST35 (Dubi):** Classic points for knee pain.

* **SP10 (Xuehai):** Invigorates Blood, reduces stasis and swelling.

* **GB34 (Yanglingquan):** Benefits tendons, relieves lateral knee pain.

* **ST34 (Liangqiu):** Acute knee pain, local circulation.

* **Ashì points** around lateral patella and subpatellar region.

**Distal points for knee/arthritis:**

* **ST36 (Zusanli):** Tonifies Qi and Blood, supports healing.

* **SP9 (Yinlingquan):** Resolves dampness, reduces joint effusion.

* **KI3 (Taixi):** Tonifies Kidney essence, strengthens bones.

* **BL23 (Shenshu):** Tonifies Kidney, supports chronic joint degeneration.

* **LR8 (Ququan):** Nourishes Liver–Kidney, benefits tendons.

**For swelling & inflammation:**

* **LI11 (Quchi):** Clears heat, reduces inflammation.

* **BL40 (Weizhong):** Benefits lumbar & knee, clears local stasis.

---

\### **Notes for Practice**

* **Needling method:**

* Local points: reducing/dispersing for swelling, effusion, tenderness.

* Distal points: reinforcing at ST36, KI3, BL23 for tonification.

* **Needle retention:** 20–30 min.

* **Adjunct therapies:**

* Electroacupuncture between EX-LE4–ST35 and SP9–ST36 may improve pain relief.

* Moxibustion at ST36, BL23, and KI3 if Kidney deficiency predominates.

* **Frequency:** 2–3 times per week initially; maintenance 1–2 times per week.

---

✅ This prescription addresses **acute symptoms (pain, swelling, effusion)** and **chronic root factors (Liver–Kidney deficiency, cartilage degeneration)** to help restore function and reduce recurrence.

Would you like me to also design a **two-phase treatment protocol** (acute pain control vs. long-term joint nourishment) for this PFA case?

**Case 10**

Thanks for the case details. Let’s carefully analyze this.

---

\### **Case Summary**

* **Patient:** 25-year-old female

* **Diagnosis:** Functional ankle instability (FAI), left side, 1-year history.

* **Symptoms:** Recurrent ankle instability.

* **Treatment choice:** Acupuncture instead of full rehab program.

---

\### **TCM Interpretation**

* **Pattern:**

* Qi and Blood deficiency of the lower limb → weak tendons and ligaments.

* Local channel obstruction due to residual dampness, Qi stagnation, or blood stasis.

* Possible Liver/Kidney deficiency → weak tendon–ligament support.

**Treatment principles:**

* Strengthen Kidney and Liver to tonify tendons/ligaments.

* Promote Qi and Blood circulation in the ankle.

* Stabilize the ankle joint, relieve residual swelling or tenderness.

---

\### **Suggested Acupuncture Point Prescription**

**Local/ankle points (stability, pain, circulation):**

* **ST41 (Jiexi) & ST36 (Zusanli):** Lower limb support and overall tonification.

* **GB40 (Qiuxu):** Strengthens ligaments and stabilizes lateral ankle.

* **BL60 (Kunlun):** Benefits ankle flexibility and pain relief.

* **SP5 (Shangqiu):** Strengthens medial ankle and supports tendons.

* **Ashì points** around lateral and medial malleolus for tenderness or residual instability.

**Distal points for lower-limb tendon/ligament support:**

* **KI3 (Taixi):** Tonifies Kidney essence, strengthens bones and ligaments.

* **BL62 (Shenmai):** Opens Yang Qiao vessel, improves ankle stability.

* **LR3 (Taichong):** Moves Liver Qi, nourishes tendons.

* **ST36 (Zusanli):** Tonifies Qi & Blood, supports overall lower-limb function.

**For chronic deficiency and prevention of recurrence:**

* **BL23 (Shenshu):** Strengthens Kidney, tonifies essence for tendon/ligament health.

* **SP6 (Sanyinjiao):** Harmonizes Liver, Kidney, Spleen; nourishes blood and tendon strength.

---

\### **Notes**

* **Needling method:**

* Local points: mild to moderate manipulation, dispersing/reducing for pain or swelling.

* Distal points: reinforcing technique to tonify deficiency (KI3, ST36, BL23).

* **Needle retention:** 20–30 min.

* **Frequency:** 2–3 times per week, possibly combined with light proprioceptive exercises if tolerated.

* **Adjuncts:** Electroacupuncture between GB40–BL60 or ST41–SP5 may improve ligament activation and joint stability.

---

✅ This prescription addresses both **local joint support for stability** and **systemic tonification** to prevent recurrence, strengthen tendons, and improve lower-limb coordination.

I can also design a **progressive protocol** for FAI: an initial phase for joint stability and pain relief, followed by a strengthening/proprioception phase that complements acupuncture. Would you like me to do that?

**Case 11**

Thank you for the detailed case. Let’s analyze carefully.

---

\### **Case Summary**

* **Patient:** 28-year-old female

* **Onset:** Sudden right-sided hearing loss, day after COVID-19 positive test.

* **Accompanying symptom:** Tinnitus (cicada-like) in the right ear.

* **Previous treatment:**

* Oral methylprednisolone 9 days (30→20→10 mg/day)

* 4 tympanic dexamethasone injections

* No improvement

* **Diagnosis:** Sudden sensorineural hearing loss (SSNHL) with tinnitus

* **PTA:** Significant impairment in right ear

* **Treatment choice:** Acupuncture

---

\### **TCM Interpretation**

* **Pattern:**

* Wind-heat or wind-phlegm obstructing the ear channels

* Kidney and Liver deficiency failing to nourish the ear

* Stagnation of Qi and Blood in the auditory channels

* **Treatment principles:**

* Open orifices, remove obstruction from auditory channels

* Nourish Kidney and Liver to support cochlear function

* Promote Qi and Blood circulation to the ear

* Calm the Shen to relieve tinnitus

---

\### **Suggested Acupuncture Point Prescription**

**Local ear points (hearing and tinnitus):**

* **SJ17 (Yifeng):** Behind ear, benefits hearing, clears channels

* **GB2 (Tinghui):** Ear disorders, tinnitus, SSNHL

* **SI19 (Tinggong):** Main point for ear conditions

* **EX-HN5 (Ermen):** Treats tinnitus, hearing loss

* **Ashì points** around the tragus and auricle for tenderness or blocked sensation

**Distal points for systemic tonification and ear support:**

* **KI3 (Taixi):** Tonifies Kidney, nourishes ear essence

* **LR3 (Taichong):** Moves Liver Qi, calms Liver wind for tinnitus

* **BL23 (Shenshu):** Strengthens Kidney essence, supports auditory function

* **ST36 (Zusanli):** Tonifies Qi and Blood, supports overall recovery

* **SP6 (Sanyinjiao):** Harmonizes Kidney, Liver, Spleen; nourishes Yin-Blood

**For tinnitus and obstruction:**

* **SJ3 (Zhongzhu):** Clears channels, relieves ear ringing

* **GB43 (Xiaxi):** Reduces excessive heat in Liver/Gallbladder channels affecting the ear

* **DU20 (Baihui):** Lifts Yang, calms Shen, improves head and ear circulation

---

\### **Notes for Application**

* **Needling method:**

* Local points: gentle lifting-thrusting to stimulate auditory channels

* Reinforcing on KI3, BL23, ST36, SP6 for deficiency

* Reducing/dispersing on SJ17, GB2, SI19 for obstruction or tinnitus

* **Needle retention:** 25–30 minutes

* **Frequency:** 5 times/week during acute phase; taper according to improvement

* **Adjuncts:** Electroacupuncture (EA) at SJ17–SI19 or GB2–SI19 may enhance cochlear blood flow and nerve function

---

✅ This prescription combines **local ear points** to directly address hearing loss and tinnitus with **distal tonifying points** to nourish Kidney and Liver essence, promote Qi/Blood flow, and support auditory recovery.

I can also create a **staged acupuncture protocol** specifically for SSNHL post-COVID, including acute phase vs. recovery phase strategies, if you want.

**Case 12**

Thank you for providing the detailed case. Let’s carefully analyze it.

---

\### **Case Summary**

* **Patient:** 68-year-old male, history of hypertension

* **Diagnosis:** Advanced pancreatic adenocarcinoma with metastases (liver, peritoneum, mesentery, pelvic fascia, terminal rectum)

* **Course:** Multiple chemotherapies (albumin paclitaxel + gemcitabine), post-chemotherapy bone marrow suppression (WBC 2.96 ×10⁹/L, platelets 60 ×10⁹/L)

* **Current issues:** Cancer-related epigastric pain, nausea, vomiting, poor appetite, sleep disturbance, fatigue

* **Analgesic therapy:** Continuous IV morphine infusion, oral & subcutaneous morphine (high doses)

* **Acupuncture goal:** Symptom relief — nausea, vomiting, opioid side effects, possibly pain relief

---

\### **TCM Interpretation**

* **Pattern:**

* Stomach and Spleen deficiency → poor digestion, nausea, vomiting, fatigue

* Liver Qi stagnation → aggravates epigastric discomfort and nausea

* Damp-heat accumulation → contributes to poor appetite, epigastric fullness

* Blood and Qi deficiency due to cancer, chemotherapy, and opioids → fatigue, weakness

* **Treatment principles:**

* Tonify Spleen and Stomach, regulate middle Jiao

* Clear stagnation, relieve nausea/vomiting

* Harmonize Qi and Blood to improve appetite and fatigue

* Calm Shen, improve sleep if disturbed by pain

---

\### **Suggested Acupuncture Point Prescription**

**1. For nausea and vomiting (main focus):**

* **PC6 (Neiguan):** Classic antiemetic point, regulates Stomach Qi

* **ST36 (Zusanli):** Strengthens Spleen/Stomach, promotes digestion, relieves nausea

* **CV12 (Zhongwan):** Regulates middle Jiao, reduces epigastric fullness

* **CV13 (Shangwan):** Clears stomach heat, relieves vomiting

* **SP4 (Gongsun):** Combined with PC6 for nausea (confluent point of Chong Mai)

**2. For epigastric pain & poor appetite:**

* **CV12 (Zhongwan):** Tonifies and regulates middle Jiao

* **ST25 (Tianshu):** Regulates intestines, abdominal pain, bowel issues

* **SP6 (Sanyinjiao):** Tonifies Spleen, harmonizes Stomach, nourishes Yin and Blood

* **LR3 (Taichong):** Moves Liver Qi, relieves epigastric distension

**3. For fatigue and weakness (post-chemotherapy, post-opioid):**

* **ST36 (Zusanli):** Strengthens Qi, supports immunity

* **CV6 (Qihai):** Tonifies Qi, supports general vitality

* **BL20 (Pishu) & BL21 (Weishu):** Tonify Spleen and Stomach

**4. For opioid side effects (nausea, constipation):**

* **ST25 (Tianshu) & ST37 (Shangjuxu):** Promote bowel movement

* **LI4 (Hegu):** Relieves pain, improves circulation and GI motility

* **SP15 (Daheng):** Assists with constipation, regulates intestines

---

\### **Notes**

* **Needling method:**

* Gentle reinforcing on tonifying points (ST36, CV6, BL20, BL21)

* Reducing or mild manipulation on points for nausea/vomiting (PC6, SP4, CV12)

* **Needle retention:** 20–30 minutes

* **Frequency:** Daily or every other day for acute nausea and vomiting; 2–3 times per week for fatigue and appetite improvement

* **Adjuncts:**

* Electroacupuncture at PC6–ST36 may enhance antiemetic effect

* Moxibustion on ST36 and CV12 for deficiency-cold patterns

* **Precautions:** Avoid deep needling in the abdomen if tumor masses or surgical sites are present; monitor platelet count due to bone marrow suppression

---

✅ This prescription primarily targets **nausea and vomiting induced by cancer and opioids**, while also **supporting digestive function, tonifying Qi/Blood, and relieving epigastric discomfort**, which can improve quality of life in the late-stage patient.

I can also draft a **daily symptom management protocol**, showing which points to use for nausea vs fatigue vs opioid-related GI symptoms if needed.

**Case 13**

Thank you for providing the case summary. Let’s carefully analyze this.

---

\### **Case Summary**

* **Patients:** 5 males, mean age \~68 years

* **History:** Radical prostatectomy (11 June 2018 – 15 Jan 2021)

* **Complaint:** Persistent post-prostatectomy urinary incontinence (PPUI) — leakage during activity, laughing, sneezing, coughing, >12 months

* **Current situation:** Some improvement post-surgery, but still significant leakage

* **Treatment choice:** Non-invasive acupuncture (patients declined more invasive options due to age/comorbidities)

---

\### **TCM Interpretation**

* **Pattern:**

* **Kidney Qi deficiency / Kidney Yang deficiency** → failure to control the bladder

* **Spleen Qi deficiency** → inability to hold urine

* **Dampness accumulation or weak lower-Jiao Qi** → urinary leakage with effort or exertion

* **Treatment principles:**

* Tonify Kidney and Spleen to strengthen bladder control

* Consolidate essence, stabilize Qi

* Regulate the lower Jiao to prevent leakage

---

\### **Suggested Acupuncture Point Prescription**

**1. Core points for PPUI (bladder control):**

* **CV4 (Guanyuan):** Strengthens Kidney and lower Jiao, tonifies essence

* **CV3 (Zhongji):** Front-Mu of bladder, regulates urination

* **BL23 (Shenshu):** Tonifies Kidney, supports bladder function

* **BL28 (Pangguangshu):** Back-Shu of bladder, regulates urination

* **KI3 (Taixi):** Tonifies Kidney Qi, consolidates essence

**2. Distal/strengthening points:**

* **SP6 (Sanyinjiao):** Strengthens Spleen, Kidney, and Liver; harmonizes lower Jiao

* **ST36 (Zusanli):** Tonifies Qi and Blood, supports overall vitality

* **DU20 (Baihui):** Raises Yang, consolidates Qi in chronic deficiency

**3. Optional points for leakage during effort (stress incontinence pattern):**

* **BL32 (Ciliao) & BL33 (Zhongliao):** Sacral points, stimulate pelvic floor musculature

* **KI6 (Zhaohai):** Regulates Yin, supports lower-Jiao function

**4. Local pelvic floor stimulation (if tolerated):**

* **Ashì points along perineum or near pelvic floor muscles** — superficial needling to strengthen muscle tone

---

\### **Notes for Practice**

* **Needling method:**

* Reinforcing technique on tonifying points (KI3, BL23, CV4, ST36)

* Gentle manipulation at sacral/pelvic points (BL32, BL33) to avoid discomfort

* **Needle retention:** 20–25 minutes

* **Frequency:** 2–3 times per week; 10–15 sessions per course, repeated based on improvement

* **Adjuncts:**

* Moxibustion on CV4 and CV3 if Kidney Yang deficiency predominates

* Kegel or pelvic floor exercises can be combined with acupuncture for better long-term outcomes

---

✅ This point combination focuses on **tonifying Kidney/Spleen, regulating lower Jiao, and supporting bladder control**, which addresses the root deficiency and improves functional urinary continence in post-prostatectomy patients.

I can also create a **progressive protocol**, starting with strengthening points and then adding sacral/pelvic stimulation for maximal improvement in stress-induced leakage.

**Case 14**

Thank you for providing the detailed case. Let’s carefully analyze it.

---

\### **Case Summary**

* **Patient:** 38-year-old female

* **Course:** 6 years of recurrent rotational vertigo

* **Symptoms:**

* Vertigo lasting several hours

* Nausea, photophobia, phonophobia

* Migrainous headaches in right temporal region (mild, rarely concurrent with vertigo)

* **Diagnosis:** Vestibular migraine (VM)

* **Previous treatment:** Sodium valproate 6 months (reduced vertigo frequency, but 8 kg weight gain)

* **Current situation:** Frequent vertigo episodes (7–8/month) after stopping valproate

* **Treatment choice:** Acupuncture

---

\### **TCM Interpretation**

* **Patterns:**

* Liver Yang rising → dizziness, vertigo, headache

* Liver and Kidney Yin deficiency → insufficient nourishment of the brain/marrow, chronic vertigo

* Phlegm-damp or wind-phlegm obstructing head → rotational vertigo, nausea

* **Treatment principles:**

* Subdue Liver Yang, nourish Liver and Kidney Yin

* Resolve phlegm-damp and wind to calm the head

* Regulate Qi and Blood, relieve nausea and headache

---

\### **Suggested Acupuncture Point Prescription**

**1. Vertigo and nausea (rotational vertigo, phlegm-damp obstruction):**

* **GB20 (Fengchi):** Clears wind, subdues Liver Yang, relieves vertigo

* **PC6 (Neiguan):** Relieves nausea and vomiting

* **ST36 (Zusanli):** Tonifies Spleen, resolves phlegm-damp, supports Qi and Blood

* **SP6 (Sanyinjiao):** Harmonizes Liver, Kidney, and Spleen; calms the head

**2. Headache (right temporal region, Liver Yang rising):**

* **GB8 (Shuaigu):** Alleviates temporal headache

* **GB41 (Zulinqi):** Subdues Liver Yang, relieves temporal/migrainous pain

* **LR3 (Taichong):** Pacifies Liver Yang, smooths Liver Qi

* **DU20 (Baihui):** Calms the Shen, raises clear Yang, improves dizziness

**3. Chronic pattern support (Liver/Kidney Yin deficiency):**

* **KI3 (Taixi):** Nourishes Kidney Yin and essence, supports brain and ear

* **BL18 (Ganshu):** Nourishes Liver Yin

* **BL23 (Shenshu):** Supports Kidney essence, tonifies lower back/brain connection

**4. Optional points for severe nausea/photophobia:**

* **EX-HN5 (Taiyang):** For temporal headache

* **Yintang (EX-HN3):** Calms Shen, relieves dizziness, photophobia

* **SJ5 (Waiguan):** Opens channels, relieves lateral head tension

---

\### **Notes**

* **Needling method:**

* Gentle reinforcing on KI3, BL18, BL23, ST36 for Yin and Qi tonification

* Mild dispersing on GB20, GB8, GB41, LR3 for Liver Yang/subduing wind-phlegm

* PC6 for nausea with lifting-thrusting or rotation

* **Needle retention:** 25–30 minutes

* **Frequency:** 2–3 times per week during active phase, 1–2 times per week for maintenance

* **Adjuncts:**

* Electroacupuncture at GB20–GB8 may enhance anti-vertigo effect

* Moxibustion on BL18/BL23 for chronic Yin deficiency

---

✅ This point combination addresses **rotational vertigo, nausea, temporal headache, and chronic Liver/Kidney Yin deficiency**, aiming to reduce the frequency and severity of VM attacks while also addressing the root pattern.

I can also prepare a **staged treatment plan** for VM, separating acute vertigo episodes from prophylactic maintenance sessions.

ChatGPT CN

案例一

根据您提供的病例描述（23岁男性，双下肢不适疼痛 2 年，夜间加重，活动后缓解，诊断为 **不宁腿综合征**），针灸治疗的选穴思路主要基于：

1. **辨证分析**

* 夜间加重、寐时难安，多与阴血不足、肝肾亏虚、筋脉失养相关。

* 活动后缓解，提示气血运行后得舒展。

* 故治法以 **滋补肝肾、养血安神、调气活络** 为主。

2. **常用针灸穴位推荐**

**主穴**：

* 足三里（ST36）：调理脾胃，益气血。

* 三阴交（SP6）：滋阴养血，调肝脾肾。

* 太溪（KI3）：补肾益精，养阴安神。

* 太冲（LR3）：疏肝理气，养血安神。

* 百会（DU20）：升阳安神，宁心定志。

* 神门（HT7）：宁心安神，改善夜间症状。

**配穴（可根据症状加减）**：

* 下肢酸胀疼痛明显：阳陵泉（GB34）、承山（BL57）。

* 伴失眠多梦：安眠穴、印堂（EX-HN3）。

* 伴腰膝酸软：关元（CV4）、肾俞（BL23）。

3. **针刺方法**

* 取穴双侧为主，以补法或平补平泻手法为宜。

* 留针 20–30 分钟，每周 2–3 次。

* 可配合艾灸足三里、三阴交、肾俞以温养肝肾。

4. **推拿/康复建议**

* 睡前温热泡脚，配合足底按摩。

* 避免咖啡因、酒精等诱发因素。

* 适度运动，但避免睡前过度剧烈活动。

---

要不要我帮您把这份针灸穴位建议整理成一个 **规范的“针灸处方格式”**（包括主穴、配穴、治法、操作方法），方便临床直接使用？

案例二

您提供的病例为 **79 岁女性，颈椎后纵韧带骨化症（C4–C6），伴有颈肩痛、上肢麻木、步态不稳及神经根/脊髓受压体征**。此类病机多为 **经络瘀阻、气血不畅，兼肝肾不足**，治疗目标为 **舒筋活络、通经止痛、益气养血、改善神经功能**。

---

\## 针灸选穴建议

**一、主穴（局部及远端相结合）**

* 颈夹脊穴（C4–C6）——疏通局部经络，缓解神经根压迫症状。

* 大椎（DU14）、风池（GB20）——舒筋活络、通经止痛。

* 合谷（LI4）、曲池（LI11）——上肢麻木疼痛。

* 外关（TE5）、落枕（EX-B2）——调和经络，改善颈项活动度。

**二、配穴（根据症状加减）**

* 上肢麻木明显：肩髃（LI15）、手三里（LI10）。

* 步态不稳、下肢无力：足三里（ST36）、阳陵泉（GB34）、悬钟（GB39）。

* 颈肩僵硬明显：肩井（GB21）、天柱（BL10）。

* 若伴失眠、焦虑：神门（HT7）、安眠穴。

**三、治法与操作**

* **治法**：舒筋活络，益气养血，健脑安神。

* **针刺方法**：局部夹脊穴以透刺为主（如C4透C5），刺激度适中；远端穴位以捻转补泻结合，行气活血为主。

* **留针时间**：20–30 分钟，每周 2–3 次。

* 可配合 **温针灸**（如足三里、阳陵泉）或 **电针**（颈夹脊配合合谷、曲池），以增强止痛、改善神经功能。

**四、康复与护理建议**

* 避免过度低头或长时间保持一个姿势。

* 睡眠时选用合适的颈椎枕。

* 适度康复训练（如颈部轻柔活动、步行训练），防止进一步功能退化。

* 定期复查影像学与神经功能，以便及时评估病情进展。

---

是否需要我帮您把这些内容整理成一个 **“规范的针灸处方格式（主穴、配穴、治法、操作方法）”**，像临床病案记录里那样？

案例三

根据您提供的病例（66 岁女性，多发性骨髓瘤，十余年全身肌肉无力酸痛，近 3 个月剧烈撕裂痛，因药物副作用担忧而选择针灸缓解症状），针灸治疗目标应定位在：

* **缓解肌肉酸痛与撕裂痛感**

* **改善肌肉无力与活动功能**

* **调节气血、增强体质、提升生活质量**

---

\## 针灸穴位建议

\### 一、主穴（调和气血、缓解肌痛）

* 足三里（ST36）：健脾益气，补虚止痛。

* 三阴交（SP6）：调和肝脾肾，缓解筋脉失养。

* 合谷（LI4）：止痛要穴，调和气血。

* 太冲（LR3）：疏肝解郁，缓解全身疼痛。

* 百会（DU20）：升阳固脱，改善乏力。

\### 二、配穴（随症加减）

* **四肢剧烈撕裂痛**：阿是穴（痛点局部）、阳陵泉（GB34）、承山（BL57）。

* **下肢无力、步行困难**：悬钟（GB39）、肾俞（BL23）。

* **全身肌肉酸痛**：肩井（GB21）、大椎（DU14）。

* **伴感冒诱发或体质虚弱**：气海（CV6）、关元（CV4）。

\### 三、治法与操作

* **治法**：补益气血，调和肝脾肾，舒筋活络，缓解疼痛。

* **针刺手法**：以平补平泻或轻补法为主，因患者年老体虚，不宜过度泻法。

* **留针时间**：20–25 分钟。

* **治疗频率**：每周 2–3 次，4 周为一疗程。

* 可配合 **艾灸（足三里、关元、气海）**，温阳益气，改善体质。

\### 四、康复与调养建议

* 保持温暖，避免寒湿侵袭。

* 进行适度轻缓运动，如散步、太极，以防关节僵硬。

* 饮食清淡，重在益气养血，避免过度劳累。

---

⚠️ 温馨提示：多发性骨髓瘤为 **血液系统恶性肿瘤**，针灸在此病例中 **仅为辅助治疗，目标是改善症状与生活质量**，并不能替代肿瘤专科规范治疗。

要不要我帮您把这份建议整理成一个 **“针灸处方格式（主穴、配穴、治法、操作方法）”**，方便直接用于临床书写？

案例四

感谢您提供的详细病例资料。此患者 **57 岁男性，椎-基底动脉系统缺血性病变（双侧 VA 闭塞+BA 重度狭窄），MRI 出现“米老鼠耳征”**，临床表现为 **肢体无力、共济失调、吞咽障碍、构音障碍、步态不稳、本体感觉障碍**，符合 **脑干-小脑-丘脑受损的椎-基底动脉系统卒中**。

针灸目标：

1. **醒脑开窍、益气活血、改善脑供血**

2. **促进神经功能恢复**（肢体运动、吞咽、言语）

3. **改善生活质量**

---

\## 针灸穴位建议

\### 一、主穴（基础调治）

* 百会（DU20）：醒脑开窍，改善脑供血。

* 风府（DU16）、风池（GB20）：通络醒脑，改善眩晕、步态不稳。

* 内关（PC6）、合谷（LI4）：调和气血，改善吞咽、言语功能。

* 足三里（ST36）：健脾益气，增强体力。

* 三阴交（SP6）：调和肝脾肾，促进康复。

\### 二、症状加减

* **肢体无力 / 共济失调**：曲池（LI11）、阳陵泉（GB34）、悬钟（GB39）。

* **吞咽障碍（呛咳、误吸）**：廉泉（CV23）、金津玉液（EX-HN12、13）、颊车（ST6）。

* **构音障碍**：舌下金津玉液、通里（HT5）。

* **步态不稳**：秩边（BL54）、承山（BL57）、昆仑（BL60）。

* **本体感觉障碍**：大椎（DU14）、肾俞（BL23）。

\### 三、治法与操作

* **治法**：益气活血，开窍通络，调和阴阳。

* **针刺方法**：以平补平泻为主，轻度捻转；年长或体虚者宜温和刺激。

* **留针时间**：20–30 分钟。

* **治疗频率**：建议每周 3–5 次，持续 4–6 周为一疗程。

* 可配合 **电针**（如百会-风池、内关-合谷、曲池-阳陵泉）促进功能恢复。

* 吞咽障碍可结合 **针刺+吞咽康复训练**（如空吞、冰刺激）。

\### 四、康复与护理建议

* 联合现代康复训练（步态训练、平衡训练、吞咽训练）。

* 饮食以软质、温热为主，防止呛咳、误吸。

* 严密监测血压、血脂、血糖，控制卒中危险因素。

* 定期影像复查，评估血管狭窄情况。

---

⚠️ 提醒：本患者为 **椎-基底动脉系统严重狭窄/闭塞**，有再次卒中风险。针灸仅为 **辅助康复手段**，仍需配合神经内科的 **药物治疗（抗血小板/调脂/稳定斑块）及二级预防措施**。

要不要我帮您把这份建议整理成一个 **规范的针灸处方（主穴、配穴、治法、操作方法、疗程安排）**，方便直接作为临床病案书写？

案例五

根据您提供的病例（42 岁男性，2022 年 8 月突发后循环脑梗死，经药物与康复治疗后，遗留 **同向性偏盲**，诊断为“中风后偏盲”），针灸干预目标应为：

1. **改善脑供血、促进神经功能重建**

2. **刺激视中枢及视路相关经络**

3. **提高患者视觉功能及生活质量**

---

\## 针灸穴位建议

\### 一、主穴（调脑醒神，改善视觉）

* 百会（DU20）：醒脑开窍，促进大脑功能恢复。

* 风池（GB20）：通经活络，改善视路供血。

* 印堂（EX-HN3）：宁神明目。

* 光明（GB37）：八会穴“络穴”，有“明目要穴”之称。

* 合谷（LI4）：调和气血，通经活络。

* 足三里（ST36）：健脾益气，补益正气。

\### 二、配穴（根据症状加减）

* **同向性偏盲、视野缩小**：睛明（BL1）、攒竹（BL2）、太阳（EX-HN5）、球后（EX-HN7）（慎操作，需有经验者）。

* **伴头晕头胀**：太冲（LR3）、悬钟（GB39）。

* **伴肢体乏力**：曲池（LI11）、阳陵泉（GB34）。

* **伴焦虑、失眠**：神门（HT7）、安眠穴。

\### 三、治法与操作

* **治法**：益气活血，醒脑明目，调和经络。

* **针刺方法**：头面部穴位以轻刺激为主；百会、风池可适度提插捻转；肢体穴位以平补平泻。

* **留针时间**：20–30 分钟。

* **治疗频率**：建议每周 3–5 次，4–6 周为一疗程。

* 可配合 **电针**（如百会-风池、光明-合谷）以增强疗效。

* 必要时结合 **眼功能康复训练**（如视野补偿训练、眼球运动训练）。

\### 四、康复与护理建议

* 进行系统的 **视功能康复训练**（追踪训练、视觉扫描训练）。

* 训练过程中注意 **安全环境**，避免跌倒。

* 调整生活习惯：规律作息、清淡饮食、避免血压血脂波动。

* 定期复查神经影像及眼科功能。

---

⚠️ 提醒：中风后偏盲属于 **中枢性视觉损伤**，恢复受限。针灸主要用于 **改善局部血流、促进神经可塑性、减轻症状**，需与 **康复训练** 联合进行。

要不要我帮您把这份建议整理成一个 **规范的针灸处方（主穴、配穴、治法、操作方法、疗程安排）**，像病案书写那样可直接应用？

案例六

好的，我来根据您提供的病例做一个中医针灸处方思路：

**病例要点**

* 23岁男性，日本患者

* 主诉：右侧颞下颌区疼痛 2 个月，张口困难，进食、说话时加重

* 查体：右侧颞肌、咬肌压痛，两侧斜方肌、头夹肌触痛

* 诊断考虑：颞下颌关节紊乱（颞颌关节功能障碍，TMD），伴肌源性疼痛

---

\## 针灸穴位建议

\### 一、主穴（局部止痛、调节颞颌功能）

* 颊车（ST6）：局部止痛，改善张口困难。

* 下关（ST7）：颞颌关节要穴，缓解关节痛。

* 太阳（EX-HN5）：止痛，疏经活络。

* 合谷（LI4）：止痛要穴，通络活血。

\### 二、配穴（兼顾颈项肌群紧张）

* 风池（GB20）、天柱（BL10）：缓解颈项僵硬及头夹肌痛。

* 肩井（GB21）：解除斜方肌紧张。

* 颞颌区阿是穴：触痛点局部取穴，活血止痛。

\### 三、治法与操作

* **治法**：活血通络，舒筋止痛，调和经气。

* **针刺方法**：局部穴位以透刺或平补平泻手法为主；颈项肩部可用捻转泻法，缓解肌肉紧张。

* **留针时间**：20–25 分钟。

* **治疗频率**：2–3 次/周，2–4 周为一疗程。

* 可配合 **温针灸**（风池、肩井）以放松颈肩肌群；或 **电针**（颊车-下关配合），增强止痛效果。

\### 四、日常调养与康复

* 避免过度咀嚼（如口香糖、坚硬食物）。

* 热敷颞颌关节区，每日 1–2 次。

* 进行颞颌关节 **张口训练与放松训练**（在专业指导下）。

* 注意保持颈肩放松，避免久坐低头。

---

⚠️ 说明：针灸对 **颞颌关节功能紊乱（TMD）** 的疗效主要体现在 **缓解局部疼痛、改善张口受限、放松颞颌及颈肩肌群**，需配合 **口腔颌面科/康复训练** 效果更佳。

要不要我帮您把这份建议整理成一个 **“针灸处方格式”**（主穴、配穴、治法、操作方法），像临床病历那样可直接使用？

案例七

根据您提供的病例信息：

**患者基本情况**

* 38岁男性

* 雄激素性脱发，病程3年+

* 既往服用非那雄胺，效果不佳

* 现症：脱发、发质细软油腻、头皮瘙痒、伴疲劳

* 中医辨证多属 **肾精不足、气血亏虚，兼痰湿阻络、血虚生风**

---

\## 针灸穴位建议

\### 一、主穴（补肾养血、固发生发）

* 百会（DU20）：升阳固脱，促进局部血液循环。

* 风池（GB20）：疏风清头，改善头皮供血。

* 肾俞（BL23）、足三里（ST36）、三阴交（SP6）：补益肝肾、健脾益气，调和气血。

* 太溪（KI3）：滋肾填精，益阴养发。

\### 二、配穴（针对症状加减）

* **头皮瘙痒、油腻**：曲池（LI11）、合谷（LI4）、太冲（LR3） → 清热疏风、理气解郁。

* **发质细软、脱发明显**：血海（SP10）、膈俞（BL17） → 活血养血，助毛发生长。

* **疲劳、体虚**：气海（CV6）、关元（CV4） → 补气固本。

* **局部刺激**：头皮针（顶区、枕区相关穴区），或头维（ST8）、四神聪（EX-HN1）。

\### 三、治法与操作

* **治法**：补益肝肾，养血生发，调和气血，疏风清热。

* **针刺手法**：头皮穴轻刺激为主；补益类穴位多用平补平泻，虚证以补法为主。

* **留针时间**：20–30 分钟。

* **治疗频率**：2–3 次/周，4–6 周为一疗程。

* 可配合 **梅花针轻叩头皮脱发区**（注意避免损伤皮肤）以促进毛囊血流。

* **艾灸**（肾俞、足三里、关元）改善体虚、疲劳。

\### 四、生活与调养

* 饮食宜清淡，多食黑芝麻、何首乌、核桃、枸杞等益肝肾、养血食物。

* 避免熬夜、过度劳累、情绪紧张。

* 少食油腻辛辣，减少头皮皮脂分泌。

* 坚持头皮按摩（每日 5–10 分钟），促进局部循环。

---

⚠️ 温馨提示：雄激素性脱发属于 **进行性脱发**，针灸治疗主要是 **改善头皮微循环、缓解头皮症状、辅助延缓脱发**，建议 **结合中西医综合治疗（如米诺地尔外用、调肝肾气血中药等）** 效果更佳。

要不要我帮您把这份内容整理成一个 **标准针灸处方格式（主穴、配穴、治法、操作方法）**，方便临床直接书写？

案例八

根据您提供的病例信息：

**患者基本情况**

* 33岁男性

* 雄激素性脱发，病程 2 年+，头顶脱发明显

* 未接受过系统治疗

* 现症：脱发、头皮瘙痒、伴疲劳、厌食

* 中医辨证：多属 **肝肾不足、气血两虚，兼有脾胃虚弱、湿热上扰**

---

\## 针灸穴位建议

\### 一、主穴（补益肝肾、养血生发）

* 百会（DU20）：升阳固脱，改善头皮供血。

* 四神聪（EX-HN1）：促进局部毛囊营养。

* 肾俞（BL23）、足三里（ST36）：补肾健脾，培元益气。

* 三阴交（SP6）：调肝脾肾，滋养气血。

* 太溪（KI3）：益肾填精，促进发根坚固。

\### 二、配穴（随症加减）

* **头皮瘙痒**：曲池（LI11）、合谷（LI4）、风池（GB20） → 清热祛风止痒。

* **疲劳、乏力**：气海（CV6）、关元（CV4） → 补气固本。

* **厌食、纳差**：中脘（CV12）、脾俞（BL20）、胃俞（BL21） → 健脾和胃，助气血化源。

* **脱发加重**：血海（SP10）、膈俞（BL17） → 活血养血，促进毛发生长。

\### 三、治法与操作

* **治法**：补益肝肾，健脾养血，疏风清热，固发生发。

* **针刺方法**：头皮穴以轻刺激为主；补益类穴位多用补法，虚证宜配合艾灸。

* **留针时间**：20–30 分钟。

* **治疗频率**：2–3 次/周，4–6 周为一疗程。

* 可辅以 **梅花针叩刺头皮脱发区**，以促毛囊血液循环。

\### 四、生活与调养

* 调整饮食：清淡为主，多食富含铁、锌、蛋白质的食物；适量黑芝麻、核桃、枸杞。

* 避免熬夜，保持情绪舒畅。

* 保持头皮清洁，减少过度油腻和辛辣食物。

* 配合适度有氧运动，改善气血运行。

---

⚠️ 提示：雄激素性脱发属于 **进行性脱发**，针灸在此主要作用是 **改善头皮循环、减轻症状、延缓进展**。若想获得更明显的生发效果，建议与 **药物（如米诺地尔外用）、中药调理** 联合应用。

要不要我帮您把这份方案整理成一个 **“针灸处方格式”（主穴、配穴、治法、操作方法）**，方便直接用于临床书写？

案例九

感谢提供完整病例。此患者 **51 岁女性，髌股关节综合征（PFA）**，以 **膝前/外侧疼痛、活动受限、下蹲上下楼加重** 为主，MRI 示软骨退变、骨髓水肿、滑膜炎、关节积液。属 **本虚标实：肝肾不足，气血亏虚；兼风湿痹阻、瘀血阻络** 之候。针灸治疗目标为：

1. **活血通络，祛风散寒，止痛消肿**

2. **补益肝肾，强筋健骨**

3. **改善关节活动度、延缓退变**

---

\## 针灸穴位建议

\### 一、主穴（膝关节局部止痛）

* 犊鼻（ST35）：膝关节局部要穴。

* 内膝眼（EX-LE4）、外膝眼（EX-LE5）：局部止痛消肿。

* 梁丘（ST34）：膝关节急性疼痛要穴，调理髌股关节。

* 阳陵泉（GB34）：筋会穴，舒筋活络，缓解外侧膝痛。

* 足三里（ST36）：健脾益气，改善下肢功能。

\### 二、配穴（随症加减）

* **膝关节肿胀明显**：阴陵泉（SP9）、血海（SP10）。

* **髌骨下疼痛、骨质退变**：鹤顶（EX-LE2）、阿是穴（局部压痛点）。

* **关节冷痛**：肾俞（BL23）、命门（DU4）配合艾灸温阳散寒。

* **久痛不愈、兼瘀阻**：膈俞（BL17）、委中（BL40） → 活血化瘀止痛。

\### 三、治法与操作

* **治法**：补益肝肾，祛风除湿，舒筋活络，消肿止痛。

* **针刺方法**：

* 膝关节周围取穴透刺或平补平泻；

* 足三里、阳陵泉以补法为主，配合艾灸；

* 阿是穴可行电针（低频）以增强止痛消炎作用。

* **留针时间**：20–30 分钟。

* **治疗频率**：每周 2–3 次，4 周为一疗程。

\### 四、康复与日常调养

* 避免频繁下蹲、爬山、上下楼过度，减少膝关节负荷。

* 可适度进行 **股四头肌训练（直腿抬高）** 增强膝关节稳定性。

* 温热理疗、泡脚、艾灸有助于缓解关节疼痛。

* 控制体重，避免膝关节额外负担。

---

⚠️ 温馨提示：此类患者软骨损伤和骨髓水肿提示 **退变性病变**，针灸主要在于 **止痛、改善功能和生活质量**，应与 **康复训练、理疗** 联合，必要时在骨科随访。

要不要我帮您把这份建议整理成一个 **“针灸处方格式”（主穴、配穴、治法、操作方法）**，方便临床直接使用？

案例十

根据您提供的病例：

**患者基本情况**

* 25 岁女性

* 主诉：左踝关节反复不稳 1 年

* 诊断：功能性踝关节不稳（FAI，functional ankle instability）

* 既往：未进行系统康复，现选择针灸治疗

**病机分析**

FAI 属中医“筋伤”“痹证”范畴，多因扭伤后经脉损伤，气血运行不畅，筋脉失养，关节稳定性下降。治疗目标为 **疏经活络、强筋壮骨、活血止痛、改善踝关节稳定性**。

---

\## 针灸穴位建议

\### 一、主穴（踝关节局部）

* 昆仑（BL60）：踝部要穴，舒筋活络，止痛消肿。

* 解溪（ST41）：踝关节要穴，改善关节灵活性。

* 申脉（BL62）：调理踝关节外侧韧带损伤。

* 丘墟（GB40）：踝关节主穴，善治踝扭伤、不稳。

* 太溪（KI3）：内踝要穴，强筋壮骨。

\### 二、配穴（随症加减）

* **踝关节酸痛明显**：阿是穴（压痛点）、阳陵泉（GB34）。

* **关节活动受限**：足三里（ST36）、悬钟（GB39）。

* **伴下肢乏力**：脾俞（BL20）、肾俞（BL23）。

\### 三、治法与操作

* **治法**：疏经通络，活血止痛，强筋健骨。

* **针刺方法**：

* 踝关节周围穴位取平补平泻法，阿是穴可配合电针。

* 下肢补益穴位以补法为主，改善整体稳定性。

* **留针时间**：20–30 分钟。

* **治疗频率**：每周 2–3 次，4 周为一疗程。

* 可配合 **温针或艾灸（昆仑、太溪）**，温经散寒，促进恢复。

\### 四、康复与日常调养

* 避免再次扭伤，运动时佩戴护踝。

* 结合 **平衡功能训练、踝关节稳定性训练（如单脚站立、弹力带抗阻训练）**，增强疗效。

* 注意休息，避免长期负重或反复剧烈运动。

---

⚠️ 提示：FAI 患者针灸可改善疼痛与不稳感，但 **康复训练是恢复踝关节稳定性的关键**。若条件允许，建议针灸与康复训练结合。

要不要我帮您把这份方案整理成一个 **“针灸处方格式”（主穴、配穴、治法、操作方法、疗程安排）**，像临床病案书写那样可直接使用？

案例十一

感谢您提供详细病例。此患者为 **28 岁女性，新冠感染后急性突发性耳聋（右耳完全性听力丧失伴耳鸣），经糖皮质激素系统及鼓膜注射治疗效果不佳**。

中医辨证多属 **风邪侵袭、痰浊阻滞或气血不足，清窍失养**。针灸治疗目标为：

1. **疏通经络、活血通窍**

2. **改善内耳供血，促进听觉神经功能恢复**

3. **缓解耳鸣，提高生活质量**

---

\## 针灸穴位建议

\### 一、主穴（耳周局部）

* 翳风（TE17）：耳后要穴，通络止痛，改善耳部循环。

* 听会（SI19）：耳前穴，直达听觉通路。

* 听宫（TE21）：疏通经络，改善听觉。

* 听息（GB2）：协助通窍止鸣。

（以上 4 穴可交替使用，局部配合阿是穴按压敏感点）

\### 二、远端配穴

* 合谷（LI4）、外关（TE5）：疏风清热，通络止鸣。

* 足三里（ST36）、三阴交（SP6）：益气养血，调和气机。

* 太冲（LR3）：平肝熄风，改善耳鸣烦躁。

* 率谷（GB8）、风池（GB20）：疏风通窍，改善耳部气血运行。

\### 三、治法与操作

* **治法**：疏风通络，活血通窍，益气养血。

* **针刺方法**：

* 耳周穴位以平补平泻法，轻度捻转提插；

* 远端穴位以补法为主，虚证可配合艾灸足三里、三阴交；

* 可结合电针（翳风–听会、合谷–太冲），促进耳部血供。

* **留针时间**：20–30 分钟。

* **治疗频率**：建议 3–5 次/周，持续 4–6 周为一疗程。

\### 四、康复与调养

* 避免劳累、情绪波动及强噪音环境。

* 保持作息规律，清淡饮食。

* 配合医生建议，定期复查听力。

---

⚠️ 提示：突发性耳聋治疗越早越好，患者已接受激素治疗但疗效欠佳，针灸在此主要作用是 **改善耳周微循环、促进残余功能恢复、缓解耳鸣**，但恢复程度因病程与损伤程度而异。

要不要我帮您把这份建议整理成一个 **“针灸处方格式”（主穴、配穴、治法、操作方法、疗程安排）**，便于直接用于临床病案书写？

案例十二

您提供的病例是一位 **68 岁男性胰腺低分化腺癌患者**，已出现 **多脏器转移、晚期癌痛并长期使用吗啡镇痛**。患者及家属希望借助针灸缓解 **吗啡相关副作用（恶心、呕吐、便秘等）**，同时可改善 **乏力、睡眠障碍**。

中医辨证属 **脾胃虚弱、气机失调，痰浊内阻**；针灸治疗目标主要是：

1. **缓解化疗、靶向及阿片类药物引起的消化道反应（恶心、呕吐、便秘）**

2. **调畅气机、改善睡眠与乏力**

3. **辅助镇痛，提高晚期生活质量**

---

\## 针灸穴位建议

\### 一、主穴（缓解恶心呕吐、调和胃气）

* 内关（PC6）：宁心安神，和胃降逆，为止呕要穴

* 足三里（ST36）：健脾和胃，增强体力，缓解乏力

* 中脘（CV12）：和胃降逆，缓解上腹不适

* 合谷（LI4）：调和气机，止痛镇静

\### 二、配穴（针对吗啡副作用及癌痛）

* **便秘**：天枢（ST25）、支沟（TE6）

* **恶心呕吐顽固**：膻中（CV17）、丰隆（ST40）

* **癌痛缓解**：阿是穴（腹部压痛点）、三阴交（SP6）、太冲（LR3）

* **失眠烦躁**：神门（HT7）、安眠（耳穴或经穴）

\### 三、治法与操作

* **治法**：和胃降逆，调气止痛，安神助眠

* **针刺方法**：

* 以平补平泻为主，轻柔进针，防止患者体虚不耐刺激

* 内关、足三里、中脘可配电针以增强止呕作用

* 三阴交、神门宜用补法，助眠安神

* **留针时间**：20–25 分钟

* **治疗频率**：每日 1 次或隔日 1 次，根据患者耐受情况灵活调整

\### 四、注意事项

* 患者晚期体质虚弱，操作需轻柔，避免强刺激

* 治疗目标以 **舒缓症状、提高生活质量** 为主，而非抗肿瘤

* 应与西医镇痛、抗肿瘤治疗并行，针灸仅作支持疗法

---

📌 总结：针灸可选择 **内关、足三里、中脘、合谷** 为核心穴，配合 **天枢、支沟** 缓解便秘，**三阴交、神门** 改善睡眠，**太冲、阿是穴** 协助镇痛。治疗需以舒适、安全为前提，重点帮助患者 **减轻吗啡副作用与癌痛相关不适**。

要不要我帮您把这份方案整理成一个 **“临床针灸处方格式”**（主穴、配穴、治法、操作、疗程），方便直接用于病案书写？

案例十三

您提供的病例为 **前列腺切除术后尿失禁（PPI, Post-Prostatectomy Incontinence）患者**，均为高龄男性，症状为 **活动、大笑、咳嗽、喷嚏时不自主漏尿**（典型应激性尿失禁），持续超过 12 个月。此类患者 **拒绝侵入性手术**，希望通过针灸改善症状。

中医辨证多属 **肾气亏虚、膀胱失约、气化不固**。针灸治疗目标为：

1. **补肾固摄**，增强膀胱控制力；

2. **调理下焦气机**，改善尿道括约肌功能；

3. **辅助盆底康复**，减轻漏尿症状。

---

\## 针灸穴位建议

\### 一、主穴（固摄小便、调理下焦）

* 中极（CV3）：膀胱募穴，调理下焦，固摄小便

* 膀胱俞（BL28）：膀胱经腧穴，益肾利尿，改善控尿功能

* 肾俞（BL23）：补肾气，助膀胱气化

* 会阴（CV1）：调任督二脉，固摄二便

\### 二、配穴（辨证加减）

* **肾气不足**：太溪（KI3）、关元（CV4）

* **脾气下陷**（伴劳累后漏尿明显）：气海（CV6）、百会（GV20）

* **兼有夜尿频繁**：三阴交（SP6）、阴陵泉（SP9）

* **焦虑失眠影响控尿**：神门（HT7）、安眠（EX-HN22）

\### 三、治法与操作

* **治法**：补肾固摄，调理膀胱气化，升提中气

* **针刺方法**：

* 中极、膀胱俞、肾俞取平补平泻，留针 20–30 分钟

* 会阴可透刺至百会–督脉方向，或配合低频电针以加强固摄效果

* 百会取提插补法，升举清阳，固摄下元

* **艾灸**：关元、气海、肾俞可配合温灸，温补下元，增强疗效

\### 四、疗程安排

* 治疗频率：每周 2–3 次

* 疗程：4 周为一疗程，连续 2–3 个疗程评估疗效

* 配合盆底肌锻炼（如凯格尔训练），协同增强控尿能力

---

📌 **总结**：

针灸治疗前列腺切除术后尿失禁推荐 **中极、膀胱俞、肾俞、会阴** 为核心穴，配合 **关元、太溪、百会** 等，根据体质和伴随症状加减。治疗宜 **补肾固摄、升提中气**，并结合康复训练，以改善生活质量。

要不要我帮您整理成一个 **标准化针灸处方（主穴+配穴+治法+疗程）**，便于直接用于病案或科研写作？

案例十四

您提供的病例是一位 **38 岁女性，前庭性偏头痛（Vestibular Migraine, VM）**，主要表现为 **反复发作旋转性眩晕**（伴恶心、畏光、畏声），伴随 **右颞区偏头痛**，但两症少同时发生。既往丙戊酸钠部分控制症状，但因体重增加停药后，眩晕复发频繁。

中医辨证多属 **肝风内动、痰浊中阻或气血不足、清窍失养**。针灸治疗目标：

1. **平肝熄风、安神止晕**

2. **化痰祛浊、开窍醒神**

3. **活血通络，改善脑部供血，缓解偏头痛**

---

\## 针灸穴位建议

\### 一、主穴（调节平衡、缓解眩晕头痛）

* 风池（GB20）：平肝熄风，通络止晕，为治眩晕头痛要穴

* 百会（GV20）：升清开窍，改善平衡感

* 太冲（LR3）：平肝理气，熄风止痛

* 合谷（LI4）：调和气血，止痛安神

\### 二、配穴（辨证加减）

* **眩晕明显、伴恶心**：内关（PC6）、丰隆（ST40）

* **偏头痛（颞区痛）**：太阳（EX-HN5）、率谷（GB8）

* **伴畏光畏声**：印堂（EX-HN3）、四神聪（EX-HN1）

* **体虚气血不足**：足三里（ST36）、三阴交（SP6）

\### 三、治法与操作

* **治法**：平肝熄风，化痰通络，益气养血

* **针刺方法**：

* 风池、百会用平补平泻法，捻转提插以醒神止晕

* 内关、丰隆配合电针，改善前庭功能、止恶心

* 太冲、合谷取泻法，缓解偏头痛

* 足三里、三阴交补法，调和气血

* **留针时间**：25–30 分钟

* **治疗频率**：每周 2–3 次，4–6 周为一疗程

\### 四、康复与调养

* 保持规律作息，避免过度劳累与情绪波动

* 饮食宜清淡，避免咖啡因、酒精、过咸食物

* 适度前庭康复训练（如眼-头协调训练）辅助改善眩晕

---

📌 **总结**：针灸治疗前庭性偏头痛可取 **风池、百会、太冲、合谷** 为核心穴，配合 **内关、丰隆、太阳、率谷** 等，根据症状灵活加减。以 **平肝熄风、化痰通络、益气养血** 为治则，辅以康复与生活调养，可帮助降低发作频率与症状强度。

要不要我帮您整理成一个 **“标准针灸处方表格”（主穴+配穴+治法+疗程）**，方便直接用于病案书写或科研？

DeepSeek CN

案例一

根据您提供的病例信息（23岁男性，诊断为不宁腿综合征，双下肢不适疼痛2年，夜间加重、活动后缓解，拒绝药物治疗），结合中医辨证和针灸治疗原则，建议以下针灸治疗方案：

---

\### **一、辨证分析**

不宁腿综合征（RLS）属中医“痹证”“痉证”范畴，多与**肝肾亏虚、气血不足、筋脉失养**或**瘀血阻络**相关。患者夜间症状加重（阴血耗损时明显），活动后缓解（气血得通），提示本虚标实之证，以肝肾阴虚、血不荣筋为主要病机。

---

\### **二、针灸取穴建议**

\#### **1. 核心穴位（主穴）**

- **足三里（ST36）**：健脾益气、调和气血，为强壮要穴，改善下肢气血运行。

- **三阴交（SP6）**：健脾养血、调补肝肾，滋阴缓解夜间症状。

- **阳陵泉（GB34）**：筋会穴，舒筋活络，缓解下肢拘急疼痛。

- **太冲（LR3）**：疏肝理气、调畅气机，改善气血瘀滞。

- **血海（SP10）**：活血化瘀，清血分郁热，缓解肢体烦扰不适。

- **委中（BL40）**（或**承山 BL57**）：膀胱经穴，舒筋通络，针对下肢后侧疼痛。

\#### **2. 配穴（随证加减）**

- **夜间加重明显**：加**照海（KI6）**（滋阴安神）、**神门（HT7）**（宁心安神）。

- **肝肾亏虚**：加**肾俞（BL23）**、**肝俞（BL18）**（背俞穴培补肝肾）。

- **瘀血阻络**：加**合谷（LI4）**、**太冲（LR3）**（开四关行气活血）。

- **烦躁不安**：加**内关（PC6）**（宽胸理气，安神定志）。

\#### **3. 特殊刺法建议**

- **电针**：于足三里-阳陵泉、血海-三阴交等配对穴位接电针仪，连续波低频刺激20分钟，增强舒筋通络效果。

- **夜间发作时应急按压**：指导患者睡前自行按压**三阴交、太冲、涌泉（KI1）**。

---

\### **三、治疗计划**

- **疗程**：每周3次，10次为1疗程，持续2-3疗程后评估疗效。

- **操作**：平补平泻法（虚实夹杂），留针30分钟，配合TDP神灯照射下肢以温通经脉。

---

\### **四、辅助建议**

1. **耳穴贴压**：神门、交感、肝、肾、皮质下，睡前按压增强疗效。

2. **生活方式调整**：避免睡前饮茶/咖啡，适度拉伸下肢（如睡前勾脚尖动作）。

3. **中药熏洗**：桂枝、红花、牛膝等活血通络药物煎汤睡前泡脚。

---

\### **五、注意事项**

- 需排除贫血、周围神经病变等继发性RLS，必要时结合实验室检查。

- 若针灸4周无效或症状加重，需重新评估诊断或联合其他疗法。

以上建议基于中医辨证及临床常见方案，具体操作需由执业医师根据患者实际反应调整。

案例二

根据您提供的病例信息（79岁女性，诊断为**颈椎后纵韧带骨化症（OPLL）**，伴颈肩疼痛、左上肢麻木、步态不稳、下肢无力及病理征阳性），患者因高龄且拒绝手术，选择针灸治疗。针灸治疗需以**缓解疼痛、疏通经络、调和气血**为目标，同时需谨慎操作，避免颈部不当操作。

---

\### **一、辨证分析**

本病属中医“**痹证**”“**痿证**”范畴，多因**肝肾亏虚、气血不足、筋骨失养**，加之**风寒湿邪侵袭**或**跌扑劳损**导致**经络瘀阻**。患者年近八旬，肝肾亏虚为本，经络瘀阻为标，属本虚标实之证。

---

\### **二、针灸取穴建议**

\#### **1. 核心穴位（主穴）**

- **颈夹脊（局部取穴）**：

- **C4-C6夹脊穴**（旁开0.5寸）：疏通局部气血，缓解神经压迫症状（**注意：浅刺或斜刺，避免深刺伤及脊髓**）。

- **远部取穴（循经取穴）**：

- **后溪（SI3）**：八脉交会穴，通督脉，主治颈项强痛、上肢麻木。

- **外关（TE5）**：三焦经络穴，通阳维脉，疏经活络，治上肢痹痛。

- **合谷（LI4）**：手阳明原穴，“面口合谷收”，通络止痛。

- **足三里（ST36）**：补益气血、强壮筋骨，改善下肢无力。

- **阳陵泉（GB34）**：筋会穴，舒筋通络，缓解肢体拘急。

- **悬钟（GB39）**：髓会穴，益髓充骨，改善步态不稳（“走在棉花上”）。

\#### **2. 配穴（随证加减）**

- **上肢麻木疼痛**：

- **曲池（LI11）**、**手三里（LI10）**（手阳明经穴，通经活络）。

- **八邪（EX-UE9）**（指间放血或针刺，缓解末梢麻木）。

- **下肢无力、步态不稳**：

- **环跳（GB30）**、**委中（BL40）**（疏通下肢经络）。

- **三阴交（SP6）**（健脾益气，滋肝肾）。

- **肝肾亏虚**：

- **肝俞（BL18）**、**肾俞（BL23）**（背俞穴，培补肝肾，**建议轻刺激或灸法**）。

- **颈部僵硬**：

- **风池（GB20）**、**天柱（BL10）**（疏风通络，**注意角度与深度**）。

\#### **3. 特殊刺法建议**

- **电针**：于颈夹脊（C4-C6）、曲池-外关、足三里-阳陵泉等接电针，疏密波（2/100Hz）刺激20分钟，增强舒筋通络效果。

- **温针灸**：于足三里、肾俞等穴行温针灸（尤其适合虚寒证），温通经脉（**注意患者感觉，避免烫伤**）。

- **梅花针叩刺**：轻叩颈部膀胱经、督脉（至潮红），改善局部气血。

---

\### **三、治疗计划**

- **疗程**：每周3次，10次为1疗程，持续2-3疗程后评估疗效。

- **操作**：

- 颈部穴位于**浅刺、斜刺**（避免直刺深刺），行平补平泻法。

- 四肢穴位可常规针刺，补法为主（因患者年老体虚）。

- 配合TDP神灯照射颈肩部以温通经脉。

---

\### **四、辅助建议**

1. **耳穴贴压**：颈椎、神门、肝、肾、交感，每日按压3次。

2. **灸法**：百会、大椎、肾俞（隔姜灸），升阳益气、滋补肾精。

3. **康复训练**：

- 颈部**适度仰头、侧屈**（避免剧烈旋转）。

- 下肢肌力训练（如坐位抬腿）、平衡训练（扶墙站立）。

4. **中药调理**：建议配合补肝肾、强筋骨中药（如独活寄生汤加减）。

---

\### **五、注意事项**

- **严禁暴力手法**：避免颈部推拿、扳法等，防止脊髓损伤。

- 密切观察病情变化，若出现症状加重（如肢体无力进展、大小便障碍），需及时影像学复查并再次评估手术必要性。

- 针灸治疗需长期坚持，以改善症状、延缓进展为目标。

以上建议基于中医辨证及针灸治疗原则，具体操作需由经验医师执行，并根据患者反应调整方案。

案例三

好的，这是一个非常复杂且严重的临床病例。针灸在此类患者的管理中主要扮演**辅助角色**，目标是**缓解疼痛、改善无力感、提高生活质量**，而非治疗原发病。治疗方案必须极其谨慎，以安全为首要原则。

以下是根据您提供的病例信息给出的针灸穴位建议：

---

\### **一、核心治疗原则**

1. **扶正固本，调和气血**：患者年高体弱，病程日久，正气严重亏虚，这是病本。

2. **通络止痛**：针对“剧烈撕裂痛”这一标急症状。

3. **健脾益肾，强健肌肉**：针对“全身肌肉无力”，脾主肌肉，肾主骨生髓。

4. **安全第一**：避免在血小板低下或有出血风险的部位进行强刺激或放血。针刺宜轻浅，多用补法，少用泻法。

---

\### **二、针灸取穴建议**

\#### **1. 核心穴位（主穴）**

-	**足三里（ST36）**：**首选要穴**。健脾益气、调和气血、扶正培元，能有效增强体力，改善肌肉无力状态。研究表明其有免疫调节作用。

-	**三阴交（SP6）**：健脾、补肝、益肾。一穴调三经，滋阴养血，对于慢性虚损性疾病至关重要。

-	**关元（CV4）**、**气海（CV6）**：**大力艾灸**。温补元阳，益气补虚，是恢复身体根本能量的关键穴位。

-	**合谷（LI4）** + **太冲（LR3）**：合称为“**开四关**”。擅长调和全身气血，行气活血，通络止痛，针对全身性疼痛有良好效果。

\#### **2. 配穴（随证加减）**

-	**针对剧烈撕裂痛（尤其下肢）**：

-	**委中（BL40）**：膀胱经合穴，“腰背委中求”，擅治下肢后侧疼痛。

-	**阳陵泉（GB34）**：筋会穴，舒筋通络，缓解筋挛和疼痛。

-	**悬钟（GB39）**：髓会穴，益髓充骨，针对骨髓病变相关的症状。

-	（*注意：肢体疼痛部位宜远端取穴为主，局部取穴需谨慎，避免刺伤肌肉或引起血肿*）

-	**针对全身肌肉无力、酸痛**：

-	**中脘（CV12）**：胃募穴，腑会，健运中焦以生气血。

-	**脾俞（BL20）**、**胃俞（BL21）**：背俞穴，艾灸最佳，强健脾胃功能。

-	**肾俞（BL23）**：补肾填精，强健筋骨。

-	**针对感冒后复发（扶正祛邪）**：

-	**大椎（GV14）**：艾灸可温阳散寒，提升阳气，增强抵抗力。

-	**肺俞（BL13）**：调补肺气，固表御邪。

---

\### **三、治疗方案与操作细节**

-	**刺法**：均采用**补法**或**平补平泻法**。进针宜轻、浅，得气即可，避免强刺激。

-	**艾灸**：**本案例艾灸至关重要**。尤其对于关元、气海、足三里、背俞穴，应采用温和灸或隔姜灸，以温通经脉，补益虚损。每次每穴灸15-20分钟。

-	**留针时间**：20-30分钟为宜，不宜过长。

-	**治疗频率**：初始阶段每周2-3次，症状稳定后改为每周1-2次。

-	**体位**：因患者虚弱且疼痛，首选**舒适卧位**，以免晕针。

---

\### **四、重要注意事项与说明**

1. ** multidisciplinary Approach（多学科协作）**：必须明确告知患者及家属，针灸是**辅助治疗**，不能替代肿瘤科的专业治疗（如化疗、靶向治疗等）。应与主管医生保持沟通。

2. **出血风险**：多发性骨髓瘤患者常伴有血小板减少，**严禁**使用三棱针放血、皮肤针叩刺等可能引起出血的操作。

3. **感染风险**：患者免疫力低下，所有操作必须严格无菌，防止感染。对于皮肤有破损、红肿或有感染迹象的部位绝对禁针。

4. **预期管理**：针灸对疼痛和无力感的缓解可能是一个渐进的过程，需设定合理预期。

5. **禁忌**：避免在骨损部位或剧烈压痛点直接针刺。

---

\### **五、非针刺疗法建议（非常适用）**

-	**艾灸**：如前所述，是此病例的首选方法。可重点灸关元、气海、足三里。

-	**耳穴贴压**：取穴：神门、皮质下、交感、肝、肾、脾、内分泌及相应疼痛部位（如膝、踝）。方便患者自行按压，持续刺激。

-	**推拿按摩**：极轻柔的肢体按摩，以舒缓肌肉紧张和疼痛，**绝对禁止暴力或深部按摩**。

**总结**：针对此患者，针灸方案应以 **“重灸轻针，远端取穴，补益为主”** 为核心原则。通过健脾益肾、补益气血来治本，通过调和气血、通络止痛来治标，在确保绝对安全的前提下，努力提高患者的生存质量。

案例四

好的，这是一个非常典型且严重的神经系统病例，诊断为**基底动脉和椎动脉狭窄/闭塞导致的脑干缺血性卒中**。针灸治疗在此类后遗症康复中扮演着重要的辅助角色，其核心目标是**疏通经络、调和气血、醒脑开窍、改善功能**。

以下是根据您提供的病例信息给出的详细针灸穴位建议：

---

\### **一、辨证分析**

患者症状集中于**肢体无力、共济失调、吞咽障碍、构音障碍**，MRI病灶在脑干、丘脑。中医辨证属“**中风-中经络**”，核心病机为：

1. **髓海空虚，脑窍瘀阻**：脑为髓海，瘀血阻滞经络，清窍失养，故见共济失调、感觉障碍。

2. **气虚血瘀，经脉失养**：气血运行无力，不能濡养筋脉，故见肢体痿软无力。

3. **风痰阻络，舌窍不利**：痰瘀阻滞舌根、咽喉部经络，故见吞咽困难、构音障碍。

本病为本虚标实之证，以**肝肾亏虚、气血不足**为本，**风、火、痰、瘀**阻络为标。

---

\### **二、针灸取穴建议**

治疗将遵循“**醒脑开窍**”为主，结合“**疏通经络、补益气血**”的原则。

\#### **1. 核心穴位（主穴）- “醒脑开窍”针法为基础**

-	**内关（PC6）**：宁心安神、疏通心脉。

-	**人中（GV26）**：醒脑开窍、升阳救逆、通督脉。

-	**三阴交（SP6）**：滋补肝肾、健脾益气生血。

\#### **2. 针对肢体无力、共济失调（治痿独取阳明）**

-	**上肢无力**：

-	**肩髃（LI15）**、**曲池（LI11）**、**手三里（LI10）**、**外关（TE5）**、**合谷（LI4）**（阳明经多气多血，主宗筋）。

-	**下肢无力、步态不稳**：

-	**环跳（GB30）**、**伏兔（ST32）**、**足三里（ST36）**、**阳陵泉（GB34）**（筋会）、**悬钟（GB39）**（髓会）、**解溪（ST41）**、**太冲（LR3）**。

-	**本体感觉障碍**：

-	**百会（GV20）**：升阳举陷、益髓充脑。

-	**大椎（GV14）**：通督脉，振奋阳气。

-	** sensory stimulation**：可于指尖、趾尖井穴（如少商、商阳、厉兑、至阴）点刺放血少许，以唤醒感觉。

\#### **3. 针对吞咽障碍、构音障碍（利咽开窍）**

-	**廉泉（CV23）**：**利咽开窍的要穴**，针刺时向舌根方向深刺。

-	**风池（GB20）**、**完骨（GB12）**、**天柱（BL10）**：针刺朝向喉结方向，深刺，可有效改善吞咽功能。

-	**金津、玉液（EX-HN12, EX-HN13）**：点刺出血，显著改善舌体灵活度。

-	**咽后壁点刺**：用压舌板压下舌体，快速点刺咽后壁3-5下，出血为度（需专业操作）。

\#### **4. 针对整体调理与气血虚弱**

-	**气海（CV6）**、**关元（CV4）**：**大力艾灸**，以大补元气，温通经脉。

-	**足三里（ST36）**：**针灸并用**，健脾益气，强壮身体，为保健要穴。

-	**脾俞（BL20）**、**肾俞（BL23）**：背俞穴，艾灸或温针灸，补益先天与后天之本。

---

\### **三、治疗方案与操作细节**

-	**针刺手法**：内关、人中用泻法；三阴交、肢体穴位用补法或平补平泻法。头面部及利咽穴位要求强刺激，以出现酸、麻、胀感为佳。

-	**电针**：适用于肢体瘫痪肌群。例如：

-	上肢：**肩髃-曲池**

-	下肢：**伏兔-足三里**、**阳陵泉-悬钟**

-	采用疏密波，强度以患者能耐受且看到肌肉规律收缩为度。

-	**艾灸**：**至关重要**。尤其对于气海、关元、足三里、背俞穴，应每次艾灸15-20分钟，以温阳益气，推动气血运行。

-	**治疗频率**：每日1次或每周5次，10次为一疗程，疗程间休息2天，连续治疗3-4个疗程后评估效果。

---

\### **四、重要注意事项**

1. **安全第一**：患者有**隐匿性误吸和呛咳**，治疗时**必须采取半卧位或坐位**，绝对禁止仰卧位针刺，防止唾液误吸。针刺廉泉、风池等穴后，应让患者休息片刻再离开。

2. **循序渐进**：患者肌力较差（左侧5-，右侧4+），初期治疗以诱发肌肉收缩、建立正确运动模式为主，避免过度疲劳。

3. **综合康复**：针灸必须与**物理治疗（PT）、作业治疗（OT）、言语和吞咽治疗（ST）** 紧密结合，才能达到最佳康复效果。

4. **情绪支持**：患者MMSE26分，认知尚可，可能因病情产生焦虑情绪。可加针**神门（HT7）**、**百会（GV20）** 以安神定志。

5. **密切观察**：治疗过程中需密切观察患者血压、心率及一般情况，防止晕针或其他不适。

**总结**：本例患者的针灸方案应是一个**系统、综合、长期**的过程。核心在于 **“醒神开窍”治其脑，“疏通阳明”治其痿，“利咽通络”治其咽**，并始终贯穿 **“温阳补气”** 的治本原则。通过积极干预，有望在改善吞咽安全、增强肌力、提高平衡能力和生活自理能力方面取得良好效果。

案例五

好的，这是一个非常典型的脑卒中后视觉通路损伤病例（同向性偏盲）。针灸治疗此病的思路是 **“通窍明目”** ，核心在于**疏通眼部及头部的经络气血，尤其是肝胆经和膀胱经，同时结合整体调理和神经康复**。

以下是根据您提供的病例信息给出的详细针灸穴位建议：

---

\### **一、辨证分析**

患者为“中风后偏盲”，病位在脑，涉及目系。中医认为“**肝开窍于目**”、“**五脏六腑之精气皆上注于目**”。后循环梗死导致瘀血阻滞于脑内视放射或枕叶视皮层，使得精血不能上荣于目，目窍失养而致盲。其核心病机为**肝肾亏虚，气血不足，瘀血阻络**。

---

\### **二、针灸取穴建议**

治疗将采取 **“局部取穴+远端取穴+辨证取穴”** 相结合的原则。

\#### **1. 核心穴位（主穴）- 通窍明目**

-	**睛明（BL1）**：**要穴**。足太阳膀胱经起始穴，直接调节眼部气血。需缓慢垂直进针，不提插捻转，出针后按压针孔。

-	**球后（EX-HN7）**：奇穴，位于眶下缘，深刺可刺激视神经，是治疗视野缺损的要穴。操作需谨慎。

-	**承泣（ST1）**：足阳明经穴，多气多血之经，濡养眼周。

-	**太阳（EX-HN5）**：奇穴，疏通局部气血。

-	**风池（GB20）**：足少阳胆经穴，针刺朝向对侧眼球方向，是治疗眼病和脑病的重要远端穴，能引气血上行。

-	**光明（GB37）**：足少阳胆经的“络穴”，**专治眼疾的要穴**，有开窍明目的作用。

\#### **2. 远端取穴 - 疏导经气，上行于目**

-	**合谷（LI4）**：手阳明经原穴，“面口合谷收”，可引导气血上注头面。

-	**太冲（LR3）**：足厥阴肝经原穴，“开四关”之一，与合谷配用可疏肝理气、调和气血、通络明目。

-	**足三里（ST36）**：足阳明经合穴，健脾益气，补益气血，使气血生化有源。

-	**三阴交（SP6）**：足三阴经交会穴，健脾、补肝、益肾，滋水涵木以养目。

\#### **3. 头针 - 直接刺激皮质功能区**

-	**枕区（视区）**：根据焦氏头针体系，取**枕上正中线（督脉）**及**枕上旁线（膀胱经）**。这是刺激视觉皮质的直接区域，对同向性偏盲有特异性疗效。

-	**运用区**：可根据患者肢体情况配用，促进整体康复。

---

\### **三、治疗方案与操作细节**

-	**针刺手法**：眼周穴位（睛明、球后、承泣）操作需极其谨慎，缓慢进针，不捻转，留针期间禁止患者睁眼或转动眼球。肢体及头部穴位可行平补平泻法。

-	**电针**：可于**风池-光明**、**合谷-太冲**等配对穴位接电针仪，采用连续波，低频率低强度刺激，以疏通经气。

-	**治疗频率**：建议每周治疗3-5次，10次为一个疗程，至少连续治疗3-4个疗程方能评估疗效。

-	**留针时间**：25-30分钟。

---

\### **四、重要注意事项与辅助建议**

1. **安全第一**：眼周血管丰富，易出血形成血肿，操作必须由经验丰富的医师执行，出针后务必充分按压。

2. **结合视觉康复训练**：告知患者针灸必须与**主动的视觉扫描训练**相结合才能取得最佳效果。例如：

-	嘱患者每日有意识地向盲侧转动眼球、转动头部，搜索盲侧物体。

-	进行阅读训练，用尺子引导视线，防止串行。

3. **管理预期**：向患者说明，视觉恢复是一个缓慢的过程，需要极大的耐心和坚持。治疗目标是扩大视野感知范围，提高生活质量，而非完全恢复正常。

4. **整体调理**：鼓励患者保持健康的生活方式，控制血压、血脂，这是防止中风再发的根本。

5. **情绪支持**：视力障碍易导致焦虑、抑郁，可加针**神门（HT7）**、**百会（GV20）** 以安神定志。

**总结**：针对此患者，针灸方案应以 **“眼周局部穴+胆经穴+头针视区”** 为核心，通过刺激局部和远端经络，调和全身气血，并直接作用于大脑视觉功能区，以期促进神经功能的代偿和修复。**坚持治疗并结合科学的视觉训练是取得疗效的关键。**

案例六

好的，这是一个非常典型的颞下颌关节紊乱病（Temporomandibular Disorders, TMD）的病例。针灸治疗此病效果显著，其核心原则是**舒筋活络、解痉止痛、调和气血**。

以下是根据您提供的病例信息给出的详细针灸穴位建议：

---

\### **一、辨证分析**

患者主诉为单侧颞下颌关节区疼痛、张口困难，且咀嚼肌群（颞肌、咬肌）和颈肩部肌群（斜方肌、头夹肌）有明显压痛。中医将其归为“**颔痛**”、“**口噤**”范畴。病机主要为：

1. **经筋劳损，气滞血瘀**：由于咀嚼、说话等过度使用，导致局部经筋（肌肉、韧带）受损，气血运行不畅，不通则痛。

2. **风寒湿邪侵袭**：可能因受凉等因素，外邪客于面部少阳、阳明经络，导致筋脉拘急，开口不利。

3. **肝郁气滞**（可能相关）：压力、情绪紧张是TMD的常见诱因，肝主筋，肝气不舒可导致筋脉拘挛。

本病病位主要在**手少阳三焦经**、**足少阳胆经**和**足阳明胃经**。

---

\### **二、针灸取穴建议**

治疗将采取 **“局部取穴 + 远端循经取穴 + 触发点（阿是穴）针刺”** 相结合的原则。

\#### **1. 局部取穴与经筋刺法（核心）**

-	**下关（ST7）**：**首选要穴**。足阳明胃经穴，位于颧弓下缘，下颌骨髁突前方，直接作用于颞下颌关节和咬肌。可直刺或向前、向后斜刺，以刺激不同部位的经筋。

-	**颊车（ST6）**：足阳明胃经穴，位于咬肌隆起处。针刺可放松咬肌，缓解痉挛。

-	**翳风（TE17）**：手少阳三焦经穴，位于耳垂后方，下颌角与乳突之间凹陷中。深层为关节后区，是治疗TMD的关键穴位。

-	**太阳（EX-HN5）**：奇穴，放松颞肌。

-	**阿是穴**：在右侧颞肌、咬肌以及两侧斜方肌和头夹肌的**最明显压痛点（触发点）** 进行针刺，采用“**经筋刺法**”（即在这些硬结或条索状物上多次浅刺或围刺），能有效松解肌肉、缓解疼痛。

\#### **2. 远端循经取穴（疏导经气）**

-	**合谷（LI4）**：手阳明大肠经原穴，“面口合谷收”，是治疗头面部所有疾病的远端要穴，有强大的镇痛效果。

-	**足三里（ST36）**：足阳明胃经合穴，引导胃经气血上行，濡养面部经筋。

-	**阳陵泉（GB34）**：足少阳胆经合穴，“筋会”，舒筋活络，缓解全身肌肉痉挛，对颈肩部斜方肌的紧张尤其有效。

-	**外关（TE5）**：手少阳三焦经络穴，通阳维脉，疏通三焦经气，针对耳周和侧头部病变。

\#### **3. 对症配穴**

-	**颈肩部压痛明显**：加**风池（GB20）**、**肩井（GB21）**（**注意：肩井穴不宜深刺**）、**天柱（BL10）**。

---

\### **三、治疗方案与操作细节**

-	**针刺手法**：

-	局部穴位（如下关、颊车）和触发点，进针后可行轻柔的捻转泻法，以出现强烈的酸胀感（“得气”）为度，从而放松肌肉。

-	远端穴位（如合谷、足三里）可行平补平泻或泻法。

-	**电针**：**强烈推荐使用**。可将一对电极连接于同侧的**下关-颊车**，另一对连接于颈肩部的压痛点上（如**风池-肩井**）。选用疏密波（D-D波），低强度刺激，时间20-25分钟。电针能产生持续的肌肉节律性收缩和放松，有效解除肌肉痉挛。

-	**温灸**：治疗结束后，可用艾条温和灸局部穴位（如下关、颊车）10-15分钟，以温通气血，加强止痛效果。

-	**治疗频率**：建议每周治疗2-3次。急性疼痛缓解后，可减至每周1次以巩固疗效。

---

\### **四、重要注意事项与患者教育**

1. **避免诱发因素**：告知患者治疗期间需：

-	进食软食，避免咀嚼硬物（如坚果、牛肉干）。

-	避免大张口（如打哈欠时用手托住下巴）。

-	注意放松，避免紧张情绪，不要紧咬牙关。

-	注意面部和颈肩部保暖，避免受凉。

2. **自我按摩**：教导患者每日轻柔按摩颞肌、咬肌和颈肩部肌肉。

3. **纠正不良习惯**：纠正单侧咀嚼、托腮等不良姿势。

4. **综合治疗**：如果患者情绪紧张是主因，可建议其结合放松训练或心理咨询。

**总结**：针对此患者，针灸方案应以 **“下关、颊车、翳风”等局部穴为主，配合“合谷、阳陵泉”等远端穴，并结合电针刺激压痛点和温灸**。通过舒筋活络、解痉镇痛，通常能取得非常显著的疗效。

案例七

好的，这是一个典型的雄激素性脱发（Androgenetic Alopecia, AGA）病例，伴有明显的血虚、肾虚和湿热证候。针灸治疗此病的思路是**补肾填精、养血生发、清热利湿**，通过整体调理来改善头皮环境，促进毛发生长。

以下是根据您提供的病例信息给出的详细针灸穴位建议：

---

\### **一、辨证分析**

患者主诉脱发、头发细软油腻、头皮发痒、疲劳。中医认为“**发为血之余**”、“**肾其华在发**”。脱发的核心病机与**肝肾不足、气血亏虚**导致发失所养相关。同时，头发油腻、头皮瘙痒是**湿热上蒸**的表现。疲劳则进一步印证了**脾肾气虚**的本质。

因此，本病是**本虚标实**之证：**肝肾亏虚、气血不足为本；湿热上泛为标**。

---

\### **二、针灸取穴建议**

治疗将采取 **“局部取穴 + 远端取穴”** 相结合的原则，标本兼治。

\#### **1. 局部取穴（重中之重）- 改善头皮气血循环**

-	**百会（GV20）**：位于头顶，督脉要穴，是诸阳之会，可升举阳气，通达头部气血。

-	**四神聪（EX-HN1）**：围绕百会的四个奇穴，加强刺激，安神益智，促进头部血液循环。

-	**头维（ST8）**：足阳明胃经与胆经交会穴，位于额角，是治疗头面部疾病的要穴。

-	**风池（GB20）**：足少阳胆经穴，疏通头部经络，引导气血上行，兼可祛风止痒。

-	**阿是穴（局部围刺）**：在脱发最明显的区域（如头顶、前额），用多根针在其周围进行**围刺**或**斜刺**（针尖朝向脱发区中心），直接刺激毛囊，激发经气。

\#### **2. 远端取穴 - 补肾、养血、清热、健脾**

-	**足三里（ST36）**：**足阳明胃经合穴**。健脾益气，补益气血，为强壮保健要穴。气血充足，头发方能得养。

-	**三阴交（SP6）**：**足太阴脾经穴**，足三阴经（肝、脾、肾）交会穴。一穴通三经，能同时调理肝、脾、肾，是养血、滋阴、生发的核心远端穴。

-	**太溪（KI3）**：**足少阴肾经原穴**。补肾填精，从根本滋养头发。

-	**血海（SP10）**：**足太阴脾经穴**。专治血分病，活血养血，“治风先治血，血行风自灭”，对头皮瘙痒有效。

-	**合谷（LI4）** + **太冲（LR3）**：合称为“**开四关**”。合谷属多气多血之阳明经，太冲为肝经原穴。两穴合用可疏肝理气，清热祛湿，调和全身气血。

---

\### **三、治疗方案与操作细节**

-	**针刺手法**：

-	头部穴位宜**浅刺、斜刺**，得气即可，不行强刺激。

-	远端肢体穴位（如足三里、三阴交）可用**补法**，强壮补虚。

-	清热利湿的穴位（如血海、太冲）可用**平补平泻法**。

-	**梅花针叩刺**：**非常重要的辅助疗法**。用梅花针轻叩脱发区域至微微潮红或少量出血，然后拔罐（用小火罐）2-3分钟，吸出少量瘀血。此法能**活血化瘀、刺激毛囊、改善局部血供**，每周1-2次。

-	**艾灸**：对于肾虚疲劳症状，可温和灸**足三里**、**肾俞（BL23）**，以温阳益气。

-	**治疗频率**：每周治疗2-3次。10次为一疗程，至少需要坚持3-4个疗程才能观察到初步效果（如脱发减少、长出细小绒毛）。生发是一个缓慢的过程，贵在坚持。

---

\### **四、重要注意事项与生活调养**

1. **管理预期**：明确告知患者，针灸治疗脱发起效较慢，需有耐心和信心，坚持治疗是成功的关键。目标是控制脱发、改善发质、促进新生，而非完全恢复到发病前状态。

2. **生活饮食调整（至关重要）**：

-	**饮食**：忌食油腻、辛辣、甜食及生冷食物，以免助湿生热。多吃黑芝麻、核桃、黑豆、桑葚等补肾养血之品。

-	**作息**：避免熬夜，保证充足睡眠。夜间11点前入睡尤为重要，因这是养血生精的关键时间。

-	**情绪**：保持心情舒畅，避免焦虑压力，肝郁气滞会加重脱发。

-	**洗护**：选择温和的洗发水，保持头皮清洁但不宜过度频繁洗头。避免使用刺激性强的化学产品。

3. **综合治疗**：建议患者继续在皮肤科医生指导下进行规范治疗（如外用米诺地尔），针灸可作为一项非常有效的辅助和增效手段。

**总结**：针对此患者，针灸方案应以 **“头部局部围刺 + 梅花针叩刺”** 为核心，直接刺激病灶，同时远端选取 **“足三里、三阴交、太溪”** 健脾补肾养血治其本，选取 **“血海、合谷、太冲”** 清热祛湿止痒治其标。**结合严格的生活饮食调养，多管齐下，方能取得最佳疗效。**

案例八

好的，这是一个典型的雄激素性脱发（AGA）病例，结合中医辨证，患者除了脱发，还伴有**头皮瘙痒、疲劳、厌食**等关键症状。这表明病机不仅是常见的**肝肾不足**，更兼有明显的**脾胃虚弱、气血生化无源**以及**血虚风燥**或**湿热上蒸**。

针灸治疗需围绕 **“健脾益气、养血生发、祛风止痒”** 的核心原则展开。

---

\### **一、辨证分析**

-	**发为血之余，肾其华在发**：脱发的根本在于**肝肾亏虚，精血不足**，无法濡养毛发。

-	**脾为气血生化之源**：患者“**厌食、疲劳**”，这是**脾胃虚弱**的明确指征。脾胃无法有效运化水谷，导致气血生化不足，进一步加剧了头发失养。

-	**血虚生风，或湿热上泛**：“**头皮瘙痒**”可能由两种原因导致：

1. 血虚不能濡养头皮，化燥生风（**血虚风燥**）。

2. 脾虚运化水湿无力，湿气郁结头部，久而化热（**湿热上蒸**）。

-	**病机总结**：**脾胃虚弱为本，肝肾不足为根，兼夹血虚风燥或湿热上泛**。治疗需健脾开路，补益在后，兼以祛邪。

---

\### **二、针灸取穴建议**

治疗方案需**强健脾胃以开源，滋补肝肾以固本，兼以疏通头部气血、祛风止痒**。

\#### **1. 核心远端取穴（治本之策）**

-	**足三里（ST36）**：**首选要穴**。足阳明胃经合穴，强力健运脾胃，补益后天之本，是解决“厌食、疲劳”的关键，为生发提供气血来源。

-	**三阴交（SP6）**：足太阴脾经穴，肝、脾、肾三经交会。一穴调三脏，既能健脾助运化，又能滋补肝肾阴血，是治疗脱发的核心穴位。

-	**中脘（CV12）**：胃之募穴，腑会。直接调理胃腑，促进消化吸收，改善厌食。

-	**血海（SP10）**：足太阴脾经穴。专治血分病，既可补血养血以润发，又可活血祛风以止痒。

-	**太溪（KI3）**：足少阴肾经原穴。补肾填精，从先天根本滋养头发。

\#### **2. 局部取穴（治标之道）**

-	**百会（GV20）**：位于头顶，属督脉，为诸阳之会。可升举阳气，通达头部气血，引气血上荣于发。

-	**四神聪（EX-HN1）**：环绕百会，加强刺激，安神益智，改善局部循环。

-	**风池（GB20）**：足少阳胆经穴。疏通头部经络，祛风清热，是止痒要穴。

-	**阿是穴（局部围刺）**：在脱发区域（头顶）周围进行**多针围刺**，针尖斜向中心，直接刺激毛囊，激发经气。

\#### **3. 随证配穴**

-	**头皮瘙痒明显**：加**合谷（LI4）**、**曲池（LI11）** 以清热祛风。

-	**若头油较多（湿热证）**：加**丰隆（ST40）** 化痰祛湿，**内庭（ST44）** 清泻胃热。

---

\### **三、治疗方案与操作细节**

-	**针刺手法**：

-	**足三里、中脘、三阴交**用**补法**，强健脾胃，补益气血。

-	头部穴位宜**浅刺、斜刺**，平补平泻。

-	止痒穴位（曲池、合谷）可用泻法。

-	**梅花针叩刺**：**强烈推荐**。用梅花针轻叩脱发区域和整个头皮，至微微潮红为度。此法能活血化瘀、刺激毛囊、止痒生发，效果显著。每周1-2次。

-	**艾灸**：对于脾胃虚寒（厌食、疲劳）表现，非常适合艾灸。

-	可温针灸**足三里**，或艾条温和灸**中脘**、**气海（CV6）**，每次15分钟，以温阳健脾，助气血化生。

-	**治疗频率**：每周2-3次。10次为一疗程，至少坚持3个疗程以上才能观察到改善（如脱发减少、瘙痒减轻、精神好转）。

---

\### **四、重要注意事项与生活调养（至关重要）**

1. **饮食调整**：这是治疗成功的一半。必须嘱患者：

-	**规律进食**，少吃多餐，食用易消化的食物（如小米粥、山药）。

-	**绝对避免**生冷、油腻、辛辣甜腻及不易消化的食物，这些会严重加重脾胃负担和湿热。

-	适当食用黑芝麻、核桃、红枣等平补气血肾精的食物。

2. **作息管理**：严禁熬夜，保证充足睡眠。夜间11点前入睡有利于养血藏精。

3. **情绪疏导**：保持心情愉悦，避免焦虑，肝气条达有助于脾胃运化。

4. **头部护理**：使用温和的洗发水，避免抓挠头皮。

**总结**：针对此患者，针灸策略应 **“以健脾开胃为先导”** ，重点刺激**足三里、中脘**解决其厌食乏力问题，为生发打下基础；同时用**三阴交、太溪**滋补根本；并用**梅花针叩刺头部**配合**风池**等穴直接治疗脱发和瘙痒。**唯有脾胃功能恢复，气血得以化生，后续的补益才能奏效。** 患者从未接受治疗，首次介入针灸，坚持综合调理，预期可取得较好效果。

案例九

好的，这是一个非常典型的髌股关节疼痛综合征（PFP）病例，并伴有明确的影像学软骨损伤和炎症表现。针灸治疗此病效果显著，其核心原则是 **“舒筋活络、活血化瘀、消肿止痛”** ，通过改善局部血液循环、缓解肌肉紧张、促进炎症吸收来缓解症状。

以下是根据您提供的病例信息给出的详细针灸穴位建议：

---

\### **一、辨证分析**

患者主诉左膝外侧及髌骨下疼痛，活动后加重，伴有积液和肿胀。中医称之为“**痹证**”或“**膝痛**”，病机主要为：

1. **劳损过度，筋脉受损**：长期爬山导致膝关节过度劳损，局部气滞血瘀，不通则痛。

2. **肝肾亏虚，筋骨失养**：年过五旬，肝肾渐虚，肝主筋，肾主骨，精血不足则筋骨萎弱，易受损伤。

3. **湿热下注，留滞关节**（根据积液和肿胀）：局部气血运行不畅，水湿停聚，化热而成湿热，导致关节肿胀、积液。

本病属**本虚标实**之证，**肝肾不足为本，气滞血瘀、湿热蕴结为标**。

---

\### **二、针灸取穴建议**

治疗将采取 **“局部取穴 + 远端循经取穴 + 辨证取穴”** 相结合的原则。

\#### **1. 局部取穴（阿是穴与经穴结合）- 疏通膝部气血**

-	**膝眼（EX-LE5）**：包括内膝眼和外膝眼。针刺膝眼可直达病所，疏通关节气机，是治疗膝关节炎性疼痛、积液的**要穴**。

-	**鹤顶（EX-LE2）**：经外奇穴，位于髌骨上缘中点凹陷处。利关节，通经络。

-	**梁丘（ST34）**：足阳明胃经郄穴，位于髌骨外上缘上2寸。郄主治急，善于治疗急性膝关节疼痛。

-	**血海（SP10）**：足太阴脾经穴，位于髌骨内上缘上2寸。活血化瘀，清血分之热，兼能健脾利湿。

-	**阳陵泉（GB34）**：足少阳胆经合穴，“**筋会**”。凡筋病皆可取其治疗，能舒筋活络，缓解膝关节周围软组织紧张，**尤其针对外侧疼痛**。

-	**足三里（ST36）**：足阳明胃经合穴，强壮保健要穴。健脾益气，补益气血，扶正固本，同时疏通下肢经络。

-	**阿是穴**：在髌骨周围、外侧副韧带等**最明显的压痛点**进行针刺，采用“**关刺**”或“**恢刺**”法（在肌腱、韧带附着点进行多向透刺），以松解粘连、缓解痉挛。

\#### **2. 远端循经取穴（上下配穴法）- 疏导经气**

-	**曲池（LI11）** + **手三里（LI10）**：手阳明大肠经穴。根据“上病下取，下病上取”及“同经相应”理论，取上肢阳明经穴可引导气血，疏通下肢阳明经气，对膝关节病有特效。

-	**悬钟（GB39）**：足少阳胆经穴，“**髓会**”。补肾健骨，舒筋通络。

\#### **3. 辨证配穴**

-	**关节肿胀、积液（湿热下注）**：加**阴陵泉（SP9）**（健脾利湿）、**三阴交（SP6）**（滋阴清热利湿）。

-	**肝肾亏虚**：加**太溪（KI3）**（补肾填精）、**肝俞（BL18）**/**肾俞（BL23）**（背俞穴，培补肝肾，可艾灸）。

---

\### **三、治疗方案与操作细节**

-	**针刺手法**：

-	局部穴位宜中等刺激，平补平泻法，以得气为度。

-	对于疼痛剧烈的阿是穴，可采用泻法。

-	远端穴位如足三里、太溪可用补法。

-	**电针**：**强烈推荐使用**。可将电极连接于一对局部穴位上，如：

-	**内膝眼-外膝眼**

-	**梁丘-血海**

-	选用疏密波（D-D波），低强度刺激，时间20-30分钟。能有效消炎镇痛、放松肌肉、促进积液吸收。

-	**温针灸**：对于寒湿或虚证明显的患者（即便有积液，若局部喜暖），可在针柄上插艾条段温灸**足三里、血海、膝眼**等穴，温通经脉、散寒除湿效果极佳。

-	**刺络拔罐**：若局部肿胀、瘀血明显，可在膝周压痛最甚处用梅花针叩刺或三棱针点刺后拔罐，放出少量瘀血，以活血化瘀、消肿止痛。每周1次。

-	**治疗频率**：建议每周治疗2-3次。10次为一疗程。

---

\### **四、重要注意事项与康复建议**

1. **休息与活动平衡**：**急性期**（疼痛肿胀明显时）应以休息为主，避免爬山、下蹲、上下楼梯等加重膝关节负荷的活动。**缓解期**可进行股四头肌等长收缩（如直腿抬高）等康复训练，以增强膝关节稳定性。

2. **保暖**：膝关节注意保暖，避免受风寒湿邪侵袭。

3. **控制体重**：减轻体重可显著降低膝关节压力。

4. **综合治疗**：针灸可与非甾体抗炎药协同使用，在针灸起效后，可能减少药物用量和依赖。也可结合物理治疗（如超短波）以增强消炎效果。

**总结**：针对此患者，针灸方案应以 **“膝眼、梁丘、血海、阳陵泉”等局部穴为主，配合“曲池、足三里”等远端穴，并灵活运用电针、温针灸等法**。通过舒筋活络、活血利湿，能有效缓解疼痛、减少积液、改善功能，帮助患者恢复日常生活。

案例十

好的，这是一个非常典型的因踝关节旧伤导致的**功能性踝关节不稳（Functional Ankle Instability, FAI）** 病例。针灸治疗的核心目标是 **“舒筋活络、强筋壮骨、调和气血、恢复稳定”** ，通过刺激相关肌肉和神经，改善本体感觉，增强关节周围的稳定性。

以下是根据您提供的病例信息给出的详细针灸穴位建议：

---

\### **一、辨证分析**

患者主诉左踝关节反复不稳定1年。中医称之为“**筋伤**”或“**踝部劳损**”。病机主要为：

1. **旧伤未愈，气血瘀滞**：初次扭伤后，气血运行不畅，瘀血留滞于关节筋络，导致恢复不彻底。

2. **肝肾亏虚，筋失所养**：肝主筋，肾主骨。长期关节不稳，活动受限，加之可能的基础不足，导致肝肾亏虚，筋骨失于濡养，故痿软无力。

3. **经筋劳损，维系失职**：踝关节周围的韧带、肌腱（经筋）因反复损伤而松弛，无法履行约束关节、维持稳定的职能。

本病属**本虚标实**之证，**肝肾不足、气血亏虚为本，气滞血瘀、经筋弛缓为标**。

---

\### **二、针灸取穴建议**

治疗将采取 **“局部取穴 + 远端循经取穴 + 平衡取穴”** 相结合的原则。

\#### **1. 局部取穴（阿是穴与经穴结合）- 疏通踝部气血，强健经筋**

-	**解溪（ST41）**：足阳明胃经经穴，位于踝关节前面中央。通经活络，主治踝关节病。

-	**丘墟（GB40）**：足少阳胆经原穴，位于外踝前下方凹陷。是治疗踝关节扭伤和外侧不稳的**核心要穴**。

-	**申脉（BL62）**：足太阳膀胱经穴，八脉交会穴（通阳跷脉），位于外踝直下凹陷。主治踝关节屈伸不利。

-	**照海（KI6）**：足少阴肾经穴，八脉交会穴（通阴跷脉），位于内踝直下凹陷。与申脉配对使用，调节阴阳跷脉，平衡内外侧肌张力，对于恢复稳定性至关重要。

-	**太溪（KI3）**：足少阴肾经原穴，位于内踝与跟腱之间凹陷。补肾强骨，滋阴养筋。

-	**昆仑（BL60）**：足太阳膀胱经经穴，位于外踝与跟腱之间凹陷。舒筋活络，常与太溪配对使用。

-	**阿是穴**：在踝关节周围**最明显的压痛点**、韧带附着点进行针刺，采用“**关刺**”或“**恢刺**”法，以松解粘连、刺激韧带修复。

\#### **2. 远端循经取穴（上下配穴法）- 疏导经气，上行下达**

-	**阳陵泉（GB34）**：足少阳胆经合穴，“**筋会**”。凡是筋病皆可取其治疗，能强健全身筋骨，尤其是下肢，是治疗关节不稳的**远端要穴**。

-	**悬钟（GB39）**：足少阳胆经穴，“**髓会**”。补肾健骨，舒筋通络。

-	**足三里（ST36）**：足阳明胃经合穴。健脾益气，补益气血，使气血充盈，筋脉得养。

-	**三阴交（SP6）**：足太阴脾经穴。健脾、补肝、益肾，一穴调三经，养血柔筋。

\#### **3. 平衡取穴与经验穴**

-	**对侧相应取穴**：可取**右侧手腕部的相应点**（如阳池TE4、阳溪LI5）进行针刺，左右交叉取穴， often能迅速缓解患侧疼痛和不适感。

-	**手臂灵骨穴、大白穴（董氏奇穴）**：重子、重仙穴附近，擅长治疗踝关节疼痛和扭伤，有奇效。

---

\### **三、治疗方案与操作细节**

-	**针刺手法**：

-	局部穴位宜中等刺激，平补平泻法，以得气为度。

-	远端穴位如足三里、三阴交可用补法；阳陵泉、悬钟用平补平泻法。

-	**电针**：**推荐使用**。可将电极连接于一对穴位上，如：

-	**丘墟-照海**（调节内外侧平衡）

-	**阳陵泉-悬钟**

-	选用疏密波（D-D波），低强度刺激，使肌肉产生节律性收缩，从而增强肌力、改善本体感觉。时间20-30分钟。

-	**温针灸**：对于虚寒明显、局部无力感的患者，可在针柄上插艾条段温灸**足三里、阳陵泉、解溪**等穴，以温阳益气、强筋健骨。

-	**治疗频率**：建议每周治疗2-3次。10次为一疗程。因患者无时间系统康复，针灸治疗需保持一定频率才能见效。

---

\### **四、重要注意事项与康复教育**

1. **必须结合功能训练**：必须向患者强调，**针灸不能替代必要的康复训练**。效果巩固的关键在于恢复神经肌肉控制（本体感觉）和增强肌力。即使时间再少，也应指导患者进行最基础的训练：

-	**本体感觉训练**：单腿站立（患肢），从睁眼到闭眼，每次30秒，每日数次。

-	**肌力训练**：弹力带抗阻踝关节外翻、内翻、背屈、跖屈。

2. **日常保护**：建议在运动或行走不平路面时，使用护踝或贴扎给予外部支持。

3. **避免再次扭伤**：告知患者注意活动环境，提高警惕性。

**总结**：针对此患者，针灸方案应以 **“丘墟、照海、解溪”等局部穴为主，配合“阳陵泉、足三里”等远端强壮穴**，并灵活运用**电针**来模拟肌肉训练。通过疏通经络、调和气血、强筋壮骨，能有效改善踝关节的稳定性，**但务必叮嘱患者克服困难，配合最低限度的康复训练，方能达到最佳和持久的效果。**

案例十一

好的，这是一个非常典型且紧急的病例（新冠病毒感染后诱发的突发性耳聋）。患者对激素治疗不敏感，听力恢复预后被告知不佳，此时针灸作为重要的替代或辅助疗法介入，思路应以 **“疏通耳窍、清肝泻火、滋肾填精、宁神开窍”** 为核心，力争抓住恢复窗口期。

以下是根据您提供的病例信息给出的详细针灸穴位建议：

---

\### **一、辨证分析**

患者为青年女性，于外感（新冠病毒）后急性起病，突发耳聋、蝉鸣。中医称之为“**暴聋**”、“**耳鸣**”。病机分析如下：

1. **外邪侵袭，窍络闭阻**：新冠病毒作为一种“疫毒”外邪，侵袭人体，上犯清窍，导致耳部经络气血壅滞，窍络闭塞，发为暴聋。

2. **肝火上炎，壅塞耳窍**：外感热毒或情志震惊（对预后失望），可引动肝火，肝火上扰，壅塞清窍。蝉鸣常为肝火或肾虚的典型表现。

3. **肾精亏虚，耳窍失养**：“肾开窍于耳”。大病之后，耗伤肾精，或患者本有肾精不足，导致耳窍失于濡养而功能失用。

本病为**本虚标实**之证，急性期以**邪气壅塞（火、瘀）** 为主，后期兼有**肾精亏虚**。

---

\### **二、针灸取穴建议**

治疗需**局部取穴与远端取穴强力结合，泻实补虚**。

\#### **1. 核心穴位（主穴）- 疏通耳窍**

-	**耳门（TE21）**、**听宫（SI19）**、**听会（GB2）**：此为治疗耳聋耳鸣的**局部三大要穴**。可交替使用，针刺时要求针感向耳深部放射，是“开窍”的关键。

-	**翳风（TE17）**：手少阳三焦经穴，位于耳垂后凹陷。疏通耳部气机，是治疗耳病的重要穴位。

-	**风池（GB20）**：足少阳胆经穴，针刺朝向对侧鼻尖或眼球方向，可疏通头部气血，引经气上行至耳。

\#### **2. 远端循经取穴 - 清泻肝胆，滋水涵木**

-	**中渚（TE3）**：手少阳三焦经输穴，“输主体重节痛”，善于治疗少阳经气不通所致的耳聋、耳鸣，是远端取穴的**首选**。

-	**外关（TE5）**：手少阳三焦经络穴，通阳维脉，疏利少阳经气。

-	**太冲（LR3）**：足厥阴肝经原穴。清泻肝火，平肝潜阳，引火下行。对于因火邪或情志因素导致的耳聋耳鸣至关重要。

-	**丘墟（GB40）**：足少阳胆经原穴，清泻胆火，疏通少阳经气。

-	**太溪（KI3）**：足少阴肾经原穴。补肾填精，滋阴潜阳，治耳聋耳鸣之根本。

\#### **3. 辨证与对症配穴**

-	**因于外感/热毒**：加**合谷（LI4）**、**曲池（LI11）** 以清热解表。

-	**震惊、焦虑、失眠**：加**神门（HT7）**、**百会（GV20）** 以宁心安神。

-	**头晕、头胀**：加**百会（GV20）**、**太阳（EX-HN5）**。

---

\### **三、治疗方案与操作细节（**非常关键**）**

-	**针刺手法**：

-	局部穴位（耳门、听宫、听会）针刺不宜过深，但要求强烈的“得气”感，**行捻转泻法**，以强力开通耳窍。

-	远端穴位如中渚、外关、太冲用**泻法**，清泻肝胆三焦之火。

-	太溪穴用**补法**，补肾填精。

-	**电针**：**强烈推荐使用**。这是治疗本病的关键技术。

-	将一对电极连接于同侧的**翳风-中渚**，或**耳门-听会**。

-	选用**疏密波（D-D波）**，强度以患者能耐受为度（可见面部肌肉轻微抽动），刺激20-30分钟。电针能持续刺激听神经和局部经络，效果远优于单纯手针。

-	**头针**：

-	选取焦氏头针的**晕听区**（从耳尖直上1.5厘米处，向前及向后各引2厘米的水平线），快速捻转刺激，对耳鸣耳聋有特效。

-	**治疗频率与疗程**：

-	**必须高频次、高强度治疗**。建议**每日治疗1次**，每周5-6次。

-	10次为一个疗程，连续治疗2-3个疗程后评估效果。本病治疗贵在坚持，即使初期效果不明显，也不应放弃。

---

\### **四、重要注意事项与患者沟通**

1. **建立信心，管理预期**：患者已被告知预后不佳，情绪极度焦虑，而“惊”又伤肾，不利于恢复。医者必须给予积极正面的鼓励，强调针灸在治疗顽固性耳聋方面的独特优势，建立医患同盟，共同坚持治疗。

2. **绝对休息，避免噪音**：告知患者必须保证充足睡眠，避免劳累，远离嘈杂环境，让听觉系统得到充分休息和修复。

3. **饮食调理**：忌食辛辣、油腻、上火之物（如辣椒、羊肉、咖啡），以免助长肝火。宜清淡饮食。

4. **综合治疗建议**：可建议患者结合**高压氧舱治疗**（如果条件允许），与针灸有协同增效作用。

**总结**：针对此患者，针灸方案应以 **“耳周三穴（耳门、听宫、听会）+ 翳风”** 局部开窍， combined with **“中渚、外关”** 疏通少阳经气，并重用 **“太冲”** 泻肝火、**“太溪”** 补肾精，同时积极采用 **“电针”** 和 **“头针”** 进行强刺激。通过多法联用，持之以恒，完全有可能创造听力恢复的奇迹。

案例十二

好的，这是一个非常严峻的晚期肿瘤姑息治疗病例。此时针灸介入的目标非常明确且务实：**不再以治疗癌症本身为目的，而是全力进行姑息支持治疗，旨在减轻极度痛苦的症状，提高患者生命最后阶段的质量**。核心任务是**缓解阿片类药物带来的严重副作用（如恶心、呕吐、便秘），并辅助镇痛、恢复部分食欲**。

以下是根据您提供的病例信息给出的详细针灸穴位建议：

---

\### **一、治疗原则**

1. **扶正固本，补益气血**：患者历经多次化疗、靶向治疗，出现严重骨髓抑制（白细胞、血小板极低），气血大亏，正气衰败。治疗需以补益为根本。

2. **降逆止呕，和胃止吐**：针对吗啡引起的恶心、呕吐。

3. **理气通腑，缓解便秘**：针对吗啡引起的肠麻痹和便秘。

4. **辅助镇痛，安神助眠**：在吗啡镇痛基础上，加强镇静安神效果，改善睡眠。

**重要提示**：操作务必轻柔，采用浅刺、补法或平补平泻法，避免强刺激。患者极度虚弱，易发生晕针。

---

\### **二、针灸取穴建议**

\#### **1. 核心穴位（主穴）- 降逆止呕、和胃止吐**

-	**内关（PC6）**：**首选要穴**。手厥阴心包经络穴，八脉交会穴（通阴维脉）。是公认的、有明确证据支持的**止呕特效穴**，能有效抑制恶心感。

-	**足三里（ST36）**：足阳明胃经合穴，强壮保健要穴。健脾和胃、补中益气、降逆止呕。能调节胃肠道功能，改善食欲。

-	**中脘（CV12）**：胃之募穴，腑会。直接调理胃腑，和胃降逆。

-	**公孙（SP4）**：足太阴脾经络穴，八脉交会穴（通冲脉）。与内关配用，能协同增强止呕、调理脾胃的功效。

\#### **2. 配穴（随证加减）**

-	**针对便秘（吗啡副作用）**：

-	**天枢（ST25）**：大肠募穴，调理肠腑，通便导滞。

-	**上巨虚（ST37）**：大肠之下合穴，“合治内腑”，专治大肠腑病。

-	**针对腹胀、上腹部不适**：

-	**章门（LR13）**：脾之募穴，脏会。调理肝脾，缓解腹胀。

-	**针对极度乏力、气血亏虚**：

-	**气海（CV6）**、**关元（CV4）**：**大力艾灸**。大补元气，温阳固脱，回救垂危之正气。这是本病例中**极其重要**的扶正方法。

-	**脾俞（BL20）**、**胃俞（BL21）**、**膈俞（BL17）**（血会）：背俞穴，可轻刺激或艾灸，补益气血。

-	**针对焦虑、失眠**：

-	**神门（HT7）**：心经原穴，宁心安神。

-	**百会（GV20）**：升举阳气，安神定志。

---

\### **三、治疗方案与操作细节**

-	**针刺手法**：全部采用**补法**或**轻柔的平补平泻法**。得气即可，不留针或短时间留针（10-15分钟）。

-	**艾灸**：**本案例的首选和核心方法**。患者阳气虚衰，艾灸的温补作用远胜针刺。

-	重点艾灸：**气海、关元、足三里**。

-	方法：采用**温和灸**，每穴灸15-20分钟，以局部皮肤红润、患者感到温暖舒适为度。艾灸的温煦作用能显著改善乏力、畏寒症状，温阳通便。

-	**按压法**：如果患者过于虚弱无法接受针刺，可由家属学习按压**内关、足三里**等主要穴位，每日多次轻柔按压，亦能起到一定效果。

-	**治疗频率**：可根据患者耐受情况，每日1次或每日2次。

---

\### **四、重要注意事项与沟通**

1. **预期管理**：必须与家属清晰沟通，针灸在此阶段的目标是**减轻痛苦、提高舒适度**，而非逆转病情。这是一个充满人文关怀的舒缓治疗。

2. **绝对安全**：患者血小板极低（60×10⁹/L），**严禁**使用三棱针点刺、梅花针叩刺等任何可能引起出血的操作。起针后要充分按压针孔。

3. **体位舒适**：患者虚弱，治疗一律采用**舒适卧位**，防止晕针和其他意外。

4. **协同治疗**：针灸是西医强效镇痛和姑息支持治疗的**辅助手段**，不应停用任何必要的西医药物。

5. **情感支持**：治疗过程也是给予患者和家属心理慰藉的过程，医者的关怀同样是一剂良药。

**总结**：针对此患者，针灸方案应 **“重灸轻针”** ，以 **“内关、足三里”** 为止呕核心，以 **“气海、关元”** 为扶正核心，通过温和的艾灸和轻柔的针刺，尽最大努力缓解患者的痛苦，让他在生命的最后旅程中能有更多的安宁和尊严。

案例十三

好的，这是一个非常典型的针灸优势病种——**前列腺术后尿失禁（Post-Prostatectomy Incontinence, PPI）**。患者为高龄男性，术后超过1年，症状持续，且拒绝有创治疗，正是针灸介入的良好时机。

针灸治疗此病的核心原则是：**“补益脾肾、固摄下元、升提固脱”**。中医认为，小便的固摄有赖于肾气的封藏和脾气的升举，手术后损伤脉络，加之年高肾气虚衰，中气下陷，导致膀胱失约，故发为尿失禁。

以下是根据病例信息给出的详细针灸穴位建议：

---

\### **一、核心针灸取穴建议**

治疗将采取 **“腹部取穴 + 腰骶部取穴 + 下肢远端取穴”** 相结合的策略，重在补益和固摄。

\#### **1. 腹部取穴 - 固摄下元，调理任脉**

-	**中极（CV3）**：膀胱募穴。直接作用于膀胱，调节膀胱气机，是治疗泌尿系疾病的**要穴**。

-	**关元（CV4）**：小肠募穴。大补元气，温肾固精，是强壮保健、固摄下元的**核心穴位**。

-	**气海（CV6）**：育之原穴。益气固脱，升阳举陷。

-	**归来（ST29）**：足阳明胃经穴，可调理下焦，增强盆底肌收缩力。

**操作要点**：针刺前务必排空膀胱。针刺方向朝向会阴部，使针感向下传导。针后加艾灸（温针灸或艾条灸）效果更佳，能温阳益气。

\#### **2. 腰骶部取穴 - 强腰健肾，振奋膀胱经气**

-	**次髎（BL32）**：**治疗尿失禁的特效穴**。足太阳膀胱经穴，位于第二骶后孔中。深刺可直接刺激盆神经和阴部神经，对恢复膀胱括约肌功能至关重要。

-	**肾俞（BL23）**：肾的背俞穴。补肾填精，培元固本。

-	**膀胱俞（BL28）**：膀胱的背俞穴。调理膀胱气机。

**操作要点**：次髎穴需深刺，使针感向前放散至小腹或会阴部。

\#### **3. 下肢远端取穴 - 补益脾肾，升提固脱**

-	**三阴交（SP6）**：足三阴经（肝、脾、肾）交会穴。一穴通三经，健脾益气、调补肝肾，是治疗下焦虚损的**关键穴**。

-	**足三里（ST36）**：足阳明胃经合穴。健脾益气，强壮全身，以助固摄。

-	**太溪（KI3）**：足少阴肾经原穴。补肾气，滋肾阴，固摄下元。

---

\### **二、治疗方案与操作细节**

-	**针刺手法**：以**补法**为主。进针得气后，行捻转补法或提插补法，刺激量中等，以患者感到舒适为度。

-	**电针**：**强烈推荐使用**。这是提高疗效的关键。

-	将一对电极连接于同侧的**次髎穴**。

-	另一对电极可连接于**中极-关元**（需使用导联线）或双侧**三阴交**。

-	**波形选择**：选用**疏密波（D-D波）**。

-	**强度**：以患者感到肌肉明显收缩但无疼痛感为度（可见腹部或会阴部肌肉节律性收缩）。每次治疗20-30分钟。

-	**艾灸**：**至关重要**。患者年高肾虚，艾灸的温补作用不可替代。

-	重点艾灸：**关元、气海、肾俞**。

-	方法：温针灸或艾条温和灸，每穴15-20分钟，使局部皮肤红润，热力深透。

-	**治疗频率与疗程**：建议每周治疗3次。10次为一个疗程，至少连续治疗3-4个疗程。本病恢复较慢，需持之以恒。

---

\### **三、重要注意事项与康复指导**

1. **盆底肌训练（Kegel运动）**：**必须叮嘱患者配合进行**。针灸的同时进行盆底肌收缩，是恢复自主控制能力的根本。方法：吸气时收缩肛门和会阴（如忍大便状），持续5-10秒，呼气时放松，重复10-15次为一组，每日进行多组。

2. **行为管理**：

-	避免一次性大量饮水，应少量多次。

-	养成定时排尿的习惯，无论有无尿意，每2小时去一次厕所，逐步延长间隔时间。

-	打喷嚏、咳嗽、大笑前，主动收缩盆底肌，以对抗腹压增高。

3. **预期管理**：向患者说明，经过系统针灸治疗，多数患者的漏尿次数和量会显著减少，生活质量明显提高，但完全治愈需要时间和耐心。

4. **体位**：治疗时采用俯卧位（针腰骶部）和仰卧位（针腹部、腿部），务必让患者舒适。

**总结**：针对此类患者，针灸方案应以 **“次髎、中极、关元”** 为核心，配合 **“三阴交、足三里”** 补益后天之本，并**积极采用电针和艾灸**进行强化刺激。通过补益脾肾、提升固脱、刺激神经，能有效改善膀胱括约肌功能，增强控尿能力。**治疗的关键在于医患配合，坚持长期规范治疗，并主动进行盆底康复训练。**

案例十四

好的，这是一个非常典型的前庭性偏头痛（Vestibular Migraine, VM）病例。针灸治疗此病具有独特优势，其核心原则是：**“平肝熄风、化痰祛湿、补益肝肾、安神定眩”**。

患者服用丙戊酸钠后体重增加8公斤，提示药物导致了痰湿内生的副作用，这也为中医辨证提供了重要线索。停用后眩晕复发，频率高达每月7-8次，说明内部风、痰、虚的病理基础依然存在。

以下是根据您提供的病例信息给出的详细针灸穴位建议：

---

\### **一、辨证分析**

患者主诉反复旋转性眩晕、恶心，伴有畏光畏声，兼有偏头痛。中医属“**眩晕**”、“**头风**”范畴。其病机如下：

1. **肝风内动**：眩晕突发突止，呈旋转性，此为“风”象，核心病机为肝阳上亢化风，或阴虚风动，上扰清窍。

2. **痰浊中阻**：伴有恶心、且服用西药后体重显著增加，此为“痰湿”内盛的表现。痰浊蒙蔽清阳，则眩晕加重。

3. **肝肾亏虚**：病程长达6年，“久病必虚”。肝藏血，肾藏精，精血亏虚，脑窍失养，是为病根。

4. **少阳经气不利**：头痛位于右颞区（少阳经循行区域），提示少阳经气郁滞。

本病为**本虚标实**之证，**肝肾不足为本，肝风、痰浊为标**。

---

\### **二、针灸取穴建议**

治疗需 **“急则治其标，缓则治其本”** 。发作期以熄风化痰、定眩止呕为主；缓解期以滋补肝肾、健脾祛湿为主。

\#### **1. 核心穴位（主穴）- 熄风定眩**

-	**风池（GB20）**：**足少阳胆经要穴**。针刺朝向对侧眼球或鼻尖，可疏通头部气血，平肝熄风，是治疗眩晕和头痛的**第一要穴**。

-	**百会（GV20）**：位于巅顶，属督脉，为诸阳之会。可升清降浊、安神定志、通络止痛。

-	**太阳（EX-HN5）**：奇穴，疏通局部气血，快速缓解颞部头痛。

-	**太冲（LR3）**：**足厥阴肝经原穴**。平肝潜阳，熄风止痉，引上逆之肝气下行，是治“风”之要穴。

-	**丰隆（ST40）**：**足阳明胃经络穴**。化痰祛湿、和胃降逆的要穴，针对痰湿和恶心症状效果显著。

\#### **2. 配穴（随证加减）**

-	**眩晕剧烈**：加**印堂（EX-HN3）** 至**鼻针**的肝点，安神定眩。

-	**恶心、呕吐**：加**内关（PC6）**（手厥阴心包经络穴，宽胸理气，降逆止呕）、**中脘（CV12）**（胃募穴，和胃降逆）。

-	**畏光、畏声**：加**合谷（LI4）**（镇静止痛）、**神门（HT7）**（宁心安神）。

-	**肝肾亏虚（缓解期）**：加**太溪（KI3）**（肾经原穴，滋阴补肾）、**三阴交（SP6）**（健脾、补肝、益肾）。

-	**少阳头痛（右颞区）**：加**率谷（GB8）**（位于耳尖直上入发际1.5寸）、**外关（TE5）**（手少阳三焦经络穴，通阳维脉）。

---

\### **三、治疗方案与操作细节**

-	**针刺手法**：

-	风池、太冲、丰隆等穴用**泻法**，以平肝、化痰、熄风。

-	太溪、三阴交等穴用**补法**，以滋补肝肾。

-	头部穴位用平补平泻法。

-	**电针**：**推荐使用**。可将一对电极连接于双侧**风池穴**，或同侧**太阳-率谷**。选用疏密波，低强度刺激，时间20-30分钟，能有效镇静、止痛。

-	**耳穴贴压**：**非常好的辅助疗法**。取穴：神门、皮质下、交感、肝、肾、枕、内耳。嘱患者每日自行按压3-5次，尤其在感觉不适时按压，以巩固疗效。

-	**治疗频率与疗程**：

-	**急性期**：发作频繁时，建议每周治疗3-5次。

-	**缓解期**：症状控制后，可逐渐减少至每周1-2次，以巩固疗效，预防复发。

-	10次为一疗程，一般需要2-3个疗程可见显著效果。

---

\### **四、重要注意事项与生活调养**

1. **识别并避免诱因**：告知患者记录眩晕日记，帮助识别并避免常见诱因，如：睡眠不足、情绪压力、特定食物（巧克力、奶酪、红酒、咖啡因）、强光闪烁、天气变化等。

2. **饮食调理**：鉴于其痰湿体质，饮食需**清淡**，忌食肥甘厚腻、生冷食物，以免助湿生痰。可适量食用薏米、山药、冬瓜等健脾利湿之品。

3. **规律作息**：保证充足的睡眠，避免熬夜和过度劳累，保持情绪稳定。

4. **循序渐进**：治疗起效后，眩晕发作频率和强度会逐渐下降，需鼓励患者建立信心，坚持治疗。

**总结**：针对此患者，针灸方案应以 **“风池、百会”** 镇静止眩为基础，重用 **“太冲”** 平肝熄风、**“丰隆”** 化痰祛湿，并根据其恶心、头痛症状配用 **“内关、太阳”** 等穴。通过标本兼治，有望显著减少眩晕发作频率、减轻发作程度，最终帮助患者摆脱对药物的依赖，且无体重增加等副作用。
