# Supplementary material for: Comparative Evaluation of ChatGPT, DeepSeek, and Physician-Generated Acupuncture Treatment Protocols Across Chinese and English Language Settings: Cross-Sectional Evaluation Study
Source: JMIR Form Res. 2026 Oct 8;10:e95851. doi: 10.2196/95851 (PMC13691461; doi:10.2196/95851)
Supplement: Supplementary file 4 [file formative_v10i1e95851_app4.docx]

**Full Statistical Report Table**

**Table S1.** Pairwise Comparison among Five Groups Based on Local Point Selection

| (I) Group | (J) Group | Mean Difference (I-J) | Std. Error | Sig.^b^ | 95% Confidence Interval for Difference^b^ | |
| --- | --- | --- | --- | --- | --- | --- |
|  |  |  |  |  | Lower Bound | Upper Bound |
| 1 | 2 | .014 | .116 | .905 | -.248 | .277 |
|  | 3 | -.157^*^ | .056 | .021 | -.284 | -.030 |
|  | 4 | .200^*^ | .072 | .022 | .037 | .363 |
|  | 5 | .157 | .100 | .150 | -.069 | .383 |
| 2 | 1 | -.014 | .116 | .905 | -.277 | .248 |
|  | 3 | -.171 | .107 | .144 | -.414 | .071 |
|  | 4 | .186 | .117 | .148 | -.079 | .451 |
|  | 5 | .143 | .115 | .244 | -.117 | .402 |
| 3 | 1 | .157^*^ | .056 | .021 | .030 | .284 |
|  | 2 | .171 | .107 | .144 | -.071 | .414 |
|  | 4 | .357^*^ | .066 | <.001 | .209 | .506 |
|  | 5 | .314^*^ | .099 | .012 | .089 | .539 |
| 4 | 1 | -.200^*^ | .072 | .022 | -.363 | -.037 |
|  | 2 | -.186 | .117 | .148 | -.451 | .079 |
|  | 3 | -.357^*^ | .066 | <.001 | -.506 | -.209 |
|  | 5 | -.043 | .100 | .678 | -.269 | .183 |
| 5 | 1 | -.157 | .100 | .150 | -.383 | .069 |
|  | 2 | -.143 | .115 | .244 | -.402 | .117 |
|  | 3 | -.314^*^ | .099 | .012 | -.539 | -.089 |
|  | 4 | .043 | .100 | .678 | -.183 | .269 |

Based on estimated marginal means
*. The mean difference is significant at the .05 level.
b. Adjustment for multiple comparisons: Least Significant Difference (equivalent to no adjustments).

**Table S2.** Pairwise Comparisons among Five Groups Based on Syndrome-based Point Selection Principle

| (I) Group | (J) Group | Mean Difference (I-J) | Std. Error | Sig.^b^ | 95% Confidence Interval for Difference^b^ | |
| --- | --- | --- | --- | --- | --- | --- |
|  |  |  |  |  | Lower Bound | Upper Bound |
| 1 | 2 | -.093 | .095 | .355 | -.308 | .123 |
|  | 3 | -.114 | .121 | .368 | -.387 | .158 |
|  | 4 | -.214 | .098 | .057 | -.436 | .008 |
|  | 5 | .200 | .143 | .195 | -.123 | .523 |
| 2 | 1 | .093 | .095 | .355 | -.123 | .308 |
|  | 3 | -.021 | .043 | .627 | -.118 | .075 |
|  | 4 | -.121 | .060 | .075 | -.258 | .015 |
|  | 5 | .293^*^ | .124 | .043 | .011 | .574 |
| 3 | 1 | .114 | .121 | .368 | -.158 | .387 |
|  | 2 | .021 | .043 | .627 | -.075 | .118 |
|  | 4 | -.100 | .070 | .187 | -.258 | .058 |
|  | 5 | .314^*^ | .118 | .026 | .047 | .582 |
| 4 | 1 | .214 | .098 | .057 | -.008 | .436 |
|  | 2 | .121 | .060 | .075 | -.015 | .258 |
|  | 3 | .100 | .070 | .187 | -.058 | .258 |
|  | 5 | .414^*^ | .123 | .008 | .136 | .693 |
| 5 | 1 | -.200 | .143 | .195 | -.523 | .123 |
|  | 2 | -.293^*^ | .124 | .043 | -.574 | -.011 |
|  | 3 | -.314^*^ | .118 | .026 | -.582 | -.047 |
|  | 4 | -.414^*^ | .123 | .008 | -.693 | -.136 |
| Based on estimated marginal means  *. The mean difference is significant at the .05 level.  b. Adjustment for multiple comparisons: Least Significant Difference (equivalent to no adjustments).  **Table S3.** Pairwise Comparisons among Five Groups Based on Neuroanatomical Point Selection Principle | | | | | | |
| (I) Group | (J) Group | Mean Difference (I-J) | Std. Error | Sig.^b^ | 95% Confidence Interval for Difference^b^ | |
|  |  |  |  |  | Lower Bound | Upper Bound |
| 1 | 2 | -.229^*^ | 0.042 | <.001 | -0.324 | -0.133 |
|  | 3 | -.121 | 0.06 | 0.075 | -0.258 | 0.015 |
|  | 4 | -.057 | 0.062 | 0.38 | -0.197 | 0.083 |
|  | 5 | .107 | 0.075 | 0.189 | -0.064 | 0.278 |
| 2 | 1 | .229^*^ | 0.042 | <.001 | 0.133 | 0.324 |
|  | 3 | .107^*^ | 0.044 | 0.038 | 0.007 | 0.207 |
|  | 4 | .171^*^ | 0.039 | 0.002 | 0.084 | 0.259 |
|  | 5 | .336^*^ | 0.078 | 0.002 | 0.16 | 0.511 |
| 3 | 1 | .121 | 0.06 | 0.075 | -0.015 | 0.258 |
|  | 2 | -.107^*^ | 0.044 | 0.038 | -0.207 | -0.007 |
|  | 4 | .064 | 0.052 | 0.244 | -0.052 | 0.181 |
|  | 5 | .229 | 0.112 | 0.071 | -0.024 | 0.481 |
| 4 | 1 | .057 | 0.062 | 0.38 | -0.083 | 0.197 |
|  | 2 | -.171^*^ | 0.039 | 0.002 | -0.259 | -0.084 |
|  | 3 | -.064 | 0.052 | 0.244 | -0.181 | 0.052 |
|  | 5 | .164 | 0.092 | 0.107 | -0.043 | 0.372 |
| 5 | 1 | -.107 | 0.075 | 0.189 | -0.278 | 0.064 |
|  | 2 | -.336^*^ | 0.078 | 0.002 | -0.511 | -0.16 |
|  | 3 | -.229 | 0.112 | 0.071 | -0.481 | 0.024 |
|  | 4 | -.164 | 0.092 | 0.107 | -0.372 | 0.043 |
| Based on estimated marginal means *. The mean difference is significant at the .05 level. b. Adjustment for multiple comparisons: Bonferroni.  **Table S4.** Pairwise Comparisons among Five Groups Based on Core Points Selection Principle | | | | | | |
| (I) Group | (J) Group | Mean Difference (I-J) | Std. Error | Sig.^b^ | 95% Confidence Interval for Difference^b^ | |
|  |  |  |  |  | Lower Bound | Upper Bound |
| 1 | 2 | -.114 | .104 | .302 | -.351 | .122 |
|  | 3 | -.021 | .092 | .820 | -.229 | .186 |
|  | 4 | .050 | .109 | .656 | -.196 | .296 |
|  | 5 | .186^*^ | .065 | .019 | .039 | .333 |
| 2 | 1 | .114 | .104 | .302 | -.122 | .351 |
|  | 3 | .093 | .059 | .152 | -.041 | .227 |
|  | 4 | .164 | .111 | .172 | -.086 | .415 |
|  | 5 | .300^*^ | .080 | .005 | .118 | .482 |
| 3 | 1 | .021 | .092 | .820 | -.186 | .229 |
|  | 2 | -.093 | .059 | .152 | -.227 | .041 |
|  | 4 | .071 | .105 | .515 | -.167 | .310 |
|  | 5 | .207^*^ | .079 | .028 | .028 | .387 |
| 4 | 1 | -.050 | .109 | .656 | -.296 | .196 |
|  | 2 | -.164 | .111 | .172 | -.415 | .086 |
|  | 3 | -.071 | .105 | .515 | -.310 | .167 |
|  | 5 | .136 | .087 | .152 | -.061 | .332 |
| 5 | 1 | -.186^*^ | .065 | .019 | -.333 | -.039 |
|  | 2 | -.300^*^ | .080 | .005 | -.482 | -.118 |
|  | 3 | -.207^*^ | .079 | .028 | -.387 | -.028 |
|  | 4 | -.136 | .087 | .152 | -.332 | .061 |

Based on estimated marginal means
*. The mean difference is significant at the .05 level. b. Adjustment for multiple comparisons: Least Significant Difference (equivalent to no adjustments).
